# Restoring Oat Nanoparticles Mediated Brain Memory Function of Mice Fed Alcohol by Sorting Inflammatory Dectin-1 Complex Into Microglial Exosomes

**DOI:** 10.1002/smll.202105385

**Published:** 2021-12-13

**Authors:** Fangyi Xu, Jingyao Mu, Yun Teng, Xiangcheng Zhang, Kumaran Sundaram, Mukesh K. Sriwastva, Anil Kumar, Chao Lei, Lifeng Zhang, Qiaohong M. Liu, Jun Yan, Craig J. McClain, Michael L. Merchant, Huang-Ge Zhang

**Affiliations:** James Graham Brown Cancer Center, Department of Microbiology & Immunology, University of Louisville, Louisville, KY 40202, USA; James Graham Brown Cancer Center, Department of Microbiology & Immunology, University of Louisville, Louisville, KY 40202, USA; James Graham Brown Cancer Center, Department of Microbiology & Immunology, University of Louisville, Louisville, KY 40202, USA; James Graham Brown Cancer Center, Department of Microbiology & Immunology, University of Louisville, Louisville, KY 40202, USA; Department of ICU, the Affiliated Huaian NO.1 People’s Hospital of Nanjing Medical University, Huaian, Jiangsu 223300, China; James Graham Brown Cancer Center, Department of Microbiology & Immunology, University of Louisville, Louisville, KY 40202, USA; James Graham Brown Cancer Center, Department of Microbiology & Immunology, University of Louisville, Louisville, KY 40202, USA; James Graham Brown Cancer Center, Department of Microbiology & Immunology, University of Louisville, Louisville, KY 40202, USA; James Graham Brown Cancer Center, Department of Microbiology & Immunology, University of Louisville, Louisville, KY 40202, USA; James Graham Brown Cancer Center, Department of Microbiology & Immunology, University of Louisville, Louisville, KY 40202, USA; Peak Neuromonitoring Associates–Kentucky, Louisville, KY 40202, USA; James Graham Brown Cancer Center, Department of Microbiology & Immunology, University of Louisville, Louisville, KY 40202, USA; Department of Medicine, Division of Gastroenterology, Hepatology and Nutrition, University of Louisville, Louisville, KY 40202, USA; Kidney Disease Program and Clinical Proteomics Center, University of Louisville, Louisville, KY 40202, USA; James Graham Brown Cancer Center, Department of Microbiology & Immunology, University of Louisville, Louisville, KY 40202, USA; Robley Rex Veterans Affairs Medical Center, Louisville, KY 40206, USA

**Keywords:** alcohol, brain inflammation, dectin-1, exosomes, food-nanoparticles, *β*-glucan, memory loss, microglial Rab11a

## Abstract

Microglia modulate pro-inflammatory and neurotoxic activities. Edible plant-derived factors improve brain function. Current knowledge of the molecular interactions between edible plant-derived factors and the microglial cell is limited. Here an alcohol-induced chronic brain inflammation model is used to identify that the microglial cell is the novel target of oat nanoparticles (oatN). Oral administration of oatN inhibits brain inflammation and improves brain memory function of mice that are fed alcohol. Mechanistically, ethanol activates dectin-1 mediated inflammatory pathway. OatN is taken up by microglial cells via *β*-glucan mediated binding to microglial hippocalcin (HPCA) whereas oatN digalactosyldiacylglycerol (DGDG) prevents assess of oatN *β*-glucan to dectin-1. Subsequently endocytosed *β*-glucan/HPCA is recruited in an endosomal recycling compartment (ERC) via interaction with Rab11a. This complex then sequesters the dectin-1 in the ERC in an oatN *β*-glucan dependent manner and alters the location of dectin-1 from Golgi to early endosomes and lysosomes and increases exportation of dectin-1 into exosomes in an Rab11a dependent manner. Collectively, these cascading actions lead to preventing the activation of the alcoholic induced brain inflammation signing pathway(s). This coordinated assembling of the HPCA/Rab11a/dectin-1 complex by oral administration of oatN may contribute to the prevention of brain inflammation.

## 1. Introduction

Compelling evidence shows that healthy edible plants including oats have important physiological roles for normal brain function and prevent neuroinflammatory processes.^[1–6]^ However, mechanistic studies in the brain have primarily focused on a single factor from edible plants, and therefore have not taken into consideration the interaction of multiple factors from edible plants that could have a direct effect on brain function.

Circulating nanoparticles such as exosomes can cross the blood-brain barrier (BBB) in both directions.^[7,8]^ However, whether oral administration of exosome like nanoparticles (ELNs) can traffic to the brain and have a direct beneficial effect on the brain is not clear. Inflammation has been strongly implicated in the pathogenesis of many brain diseases including alcohol use disorder (AUD).^[9,10]^ Microglia in the brain become chronically activated by numerous factors.^[11–13]^ Considerable research in the last couple of decades has increased the knowledge of the scientific community about the paramount importance of brain inflammation in the pathogenesis of many of the systemic manifestations of alcoholism. Therefore, alcoholism can be viewed as an inflammatory condition, a concept which opens the possibility of using new therapeutic weapons to treat some of the complications of this frequent disease.

Dectin-1 signaling has been implicated in multiple neuroimmune contexts and dynamic upregulation of Dectin-1 in microglia under pathogenic conditions has become apparent.^[14]^ Dectin-1 possesses an immunoreceptor tyrosine-based activation motif (ITAM)-like motif (also called a “hemi-ITAM”) in its cytoplasmic moiety and activates spleen tyrosine kinase (Syk), eventually activating NF-*κ*B to induce the expression of proinflammatory molecules such as TNF*α* and IL-1*β*. Dectin-1 specifically recognizes fungi *β*-glucans found in fungal cell walls and is one of the primary sensors of pathogenic and commensal fungi in humans.^[15,16]^ Functional dectin-1 signaling requires the *β*-glucan to be particulate, and the structure of the *β*-glucan particulate is also key to dictating the magnitude of the inflammatory immune response. Besides dectin-1, fungi *β*-glucan can also interact with complement receptor (CR3) and TLR-2/6 and trigger an inflammatory response.^[17]^ Whether nano sized oat ELNs can modulate dectin-1 signaling via oatN *β*-glucan in the oatN recipient cells is not known.

HPCA or hippocalcin, is a member of the neuronal calcium sensor (NCS) protein family.^[18–20]^ The myristoyl-containing hydrophobic N-terminus region allows HPCA localize to membranes where it can interact with downstream targets.^[21–25]^ In addition to its role in the control of apoptosis, HPCA has been shown to be involved in neuronal excitability, regulation of neurite outgrowth and gene transcription, and long-term depression.^[26]^

In this study, as proof-of-concept, we demonstrate that oat ELN (oatN) prevents brain inflammation induced by alcohol in a mouse AUD model via the oatN *β*-glucan mediated HPCA pathway (graphical abstract). We provide cellular and molecular insights as to how oatN modulates neuroimmune function via the HPCA/Rab11a/dectin-1 complex to prevent brain inflammation that is induced by consuming alcohol. We found that oatN *β*-glucan is required for oatN to be taken up by microglial cells via HPCA/*β*-glucan interaction. Subsequently to this action it is recruited by Rab11A to the HPCA/*β*-glucan complex which is a critical step for bringing Golgi dectin-1 to the recycling endosome and exosome pathways. Collectively, these results suggest that oral taking the edible exosomes-like nanoparticles such as oatN has anti-inflammatory effects on the brain and can be utilized for the prevention/treatment of brain inflammation related disease.

## 2. Result

### 2.1. Oral Administration of Oat Nanoparticles Trafficking to the Brain Is Preferentially Taken up by Microglial Cells

Circulating nanoparticles, such as exosomes can migrate into the brain.^[27]^ Recently edible plant ELNs have been identified.^[28,29]^ Whether orally given ELNs can traffic to the brain and have biological effects on brain function are not known. Oat extracts have anti-inflammatory effects that prevent brain damage.^[30]^ However, the molecular mechanism underlying the anti-inflammatory effect has not been fully explored. Therefore, we began to characterize oat ELNs using a previously described method.^[28]^ Optiprep gradient purified oat ELNs were examined using electron microscopy (Figure S1A,B, Supporting Information) and size distribution and quantity of oat ELNs were analyzed using a Nanosight NS300 (Figure S1B, Supporting Information). Interestingly, unlike ELNs we have previously analyzed,^[31]^ oat ELNs do not have detectable RNA (Figure S1C, Supporting Information), but had detectable proteins, lipids, and polysaccharides (Figure S1D–H, Tables S1–S3, Supporting Information). The oat nanoparticles are noted as oatN. The yield of oatN was also calculated and expressed as g/kg of oat flour (Figure S1A, Supporting Information).

**Figure 1.**
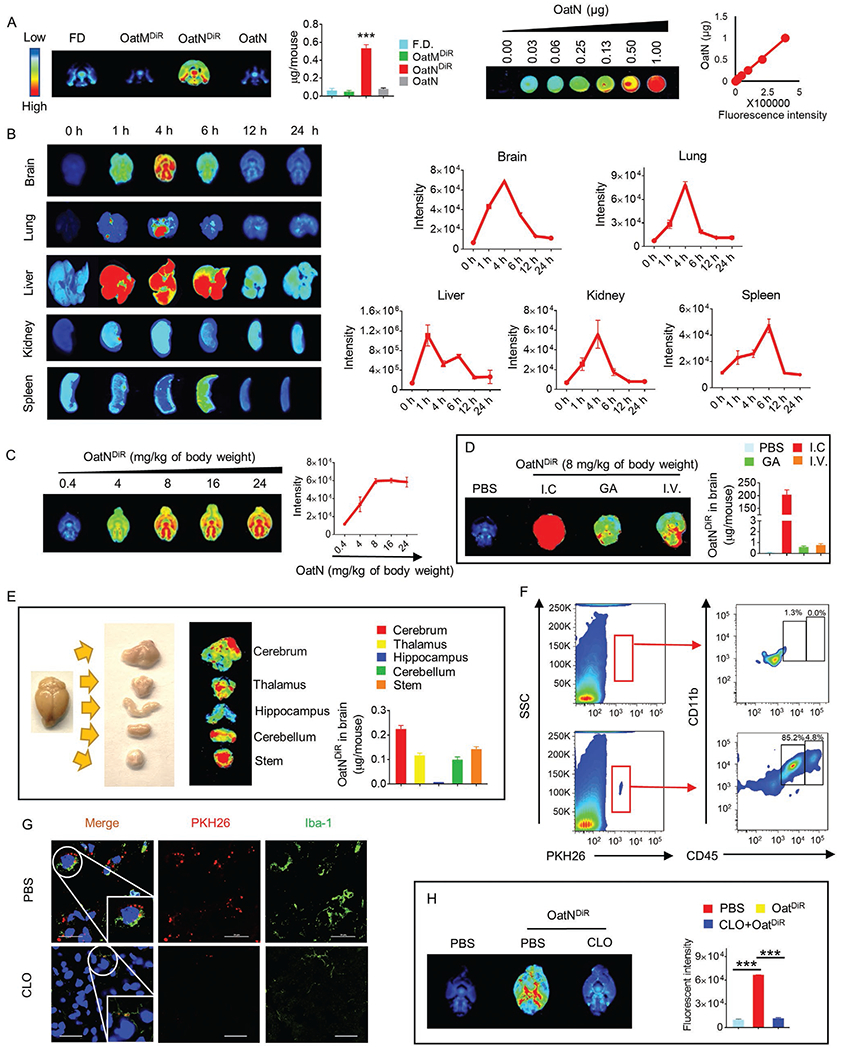
Oral administration of oat nanoparticles traffic to the brain and are preferentially taken up by microglial cells. A) DiR labeled oatM or oatN was orally administrated to C57BL/6 mice (8 mg kg^−1^ of body weight). 4 h after the gavage, whole brains were excised and scanned using an Odyssey imager. DiR free dye (FD) and oatN without labeling were used as controls. Imaging results represent 1 out of 3 independent experiments (left panel, *n* = 3 mice per group). Amount of free dye, oatM, or oatN in the brain was calculated based on a standard curve made from DiR labeled oatM or oatN (Figure S1A, Supporting Information, and Figure 1A, right panel). B) Ex vivo images of the lung, liver, kidney, spleen, and brain from mice after receiving a gavage of DiR dye labeled oatN at different time points (*n* = 3 mice per group). C) Dose-dependent fluorescence signal intensity of brain from mice after receiving a gavage of DiR dye labeled oatN (*n* = 3 mice per group). Bar graphs represent the mean of imaging intensity from 3 mice/group. D) Intracranial injection (I.C), gavage given (GA), and intravenous injection (I.V.) of oatN (8 mg kg^−1^ of body weight) was done, and imaging was done after 4 h using an Odyssey scanner. Results represent 1 out of 3 independent experiments (left panel, *n* = 3 mice per group). Amount of oatN in the brain was calculated based on the standard curve shown in the right panel of Figure 1A. E) Mice (*n* = 3 per group) were sacrificed 4 h after being gavage-given DiR or PKH26-labeled oatN, five portions of brain tissue (cerebrum, thalamus, hippocampus, cerebellum, and stem) were dissected out and scanned. Amount of oatN in five portions were calculated based on the standard curve shown in the right panel of Figure 1A. F) Mouse microglial cells were isolated. PKH26 positive cells were gated, and microglial ells were determined by flow cytometry analysis. Results represent 1 out of 5 independent experiments. Flow cytometry analysis shows the % of CD45^mid^CD11b^+^ and CD45^+^CD11b^+^ cells (inset). G) Forty-eight hours after intracisternal injection of mice with clodronate (CLO, 1 mL stock diluted in 9 mL of PBS, stock 5 mg mL^−1^, 10 μL/mice), they were gavage-given PKH26-labeled oatN (8 mg kg^−1^ of body weight). 4 h after gavaging, brain tissues were excised and fixed. Sectioned brain tissue was stained with anti-Iba-1, a microglial cell marker (green), confocal imaging assays were performed, and representative images were photographed (*n* = 5). Scale bars, 20 mm. H) Fluorescent images of mice treated with DiR-labeled oatN (8 mg kg^−1^ of body weight) after CLO injection (1 mL stock diluted in 9 mL of PBs, stock 5 mg mL^−1^, 10 μL/mice) in the hemispheres of the brain. The intensity of the imaging signals was quantified. Bar graphs represent the mean of imaging intensity from 3 mice/group and SEM (*n* = 3 mice/group). ***p < 0.001 by ANOVA test.

Nanoparticles including exosomes have been reported to traffic to the brain.^[27]^ To determine whether oatN can traffic into the brain, oatN were labeled with DiR dye. Mice were gavage administrated DiR dye labeled oatN (oatN^DiR^, 8 mg kg^−1^ of body weight), equal amounts of DiR dye labeled oat microparticles (oatM) or unlabeled oatN and DiR free dye as controls (Figure 1A; Figure S2A, Supporting Information). A much stronger fluorescent signal was detected in the oatN^DiR^-treated mice brains than with the two control groups when using the Odyssey imaging analysis system (Figure 1A). In addition, oatN^DiR^ also trafficked to the liver (Figure 1B). OatN^DiR^ was initially detected in the brain at 1 h post gavage, reached a maximum at 4 h and then the fluorescent intensity gradually decreased after 6 h (Figure 1B). Increasing the amount of oatN^DiR^ administrated increased the intensity of the DiR signal which plateaued at a dose of 8 mg kg^−1^ of body weight. The liver had the strongest oatN^DiR^ signal at 1 h post gavage-administration (Figure 1B,C). OatN^DiR^ was also detected in the peripheral blood of mice (Figure S2B, Supporting Information).

To ensure the brain signal we detected was not free DiR dye dissociated from the oatN^DiR^ during trafficking from the gut to the brain, we evaluated the stability of oatN in simulated gastrointestinal digestion model in vitro. Sizes and distribution of oatN after simulated gastrointestinal buffer treatment were slightly large-scale shifted according to the data generated from the Nanosight analysis (Figure S2C, Supporting Information). Our results are consistent with previous observations, which reported that nanoparticles aggregated extensively when exposed to gastric fluid in vitro.^[32]^ Second, to prove whether oatN can cross the blood-brain barrier (BBB), we assessed the permeability in an in vitro BBB model using primary endothelial cells from mice brains. Our data showed the fluorescent signals in the group treated with PKH26 labeled oatN are higher than the signals in the PBS control group (Figure S2D, Supporting Information), suggesting that oatN can cross the BBB. To address the question as to how oatN crosses the BBB, we determined whether the transfer of oatN crossing the BBB occurs in an active or passive manner. The results show that oatN can pass the BBB at both 37 and 4 °C, suggesting oatN cross BBB via free diffusion and an active transport manner. In addition, ethanol treatment significantly enhances oatN passing through the BBB (Figure S2D, Supporting Information). Lastly, oatN proteins were covalently labeled with ExoGlow. After gavage-giving the ExoGlow labeled oatN to mice for 6 h, the mice were perfused, and the brain tissue was resected. The imaging results demonstrated that the oatN was trafficking into brain (Figure S2E, Supporting Information).

Next, we quantified the efficiency of gavage-given oatN^DiR^ trafficking to the brain by calculating the ratio of the intensity of fluorescent signal in the brain of mice gavage-given or intravenous injection given versus an intracranial injection of oatN using a previous described protocol.^[33]^ The results indicated that 0.3% of gavage-given oatN^DiR^ were trafficked to the brain within 4 h (Figure 1D). Within the brain the strongest signal of oatN^DiR^ was detected in the brain cerebrum, a moderate signal was detected in the thalamus, stem, and cerebellum portions, and the weakest signal was detected in the hippocampus region (Figure 1E). We next identified brain cells targeted (recipient cells) by oatN. The mice were gavage-given fluorescent dye PKH26-labeled oatN. Flow cytometry analysis showed that most of the PKH26 positive cell population displayed the CD45 intermediate expression and CD11b high expression (Figure 1F). Further, we stained frozen sections of mouse brain with Iba-1 (a microglial cell specific marker) and performed confocal imaging assay. As Figure 1G shows, double positive PKH26^+^ and Iba-1^+^ cells were detected in brain tissue sections. To further determine if microglial cells play a causative role in oatN trafficking to the brain, brain macrophages were depleted with clodronate (CLO) before gavage administration of PKH26 labeled or DiR labeled oatN. Confocal data (Figure 1G) and brain imaging analysis (Figure 1H) indicate that oatN is selectively take up by brain microglial cells.

### 2.2. OatN Inhibits Alcohol-Induced Brain Inflammation

**Figure 2.**
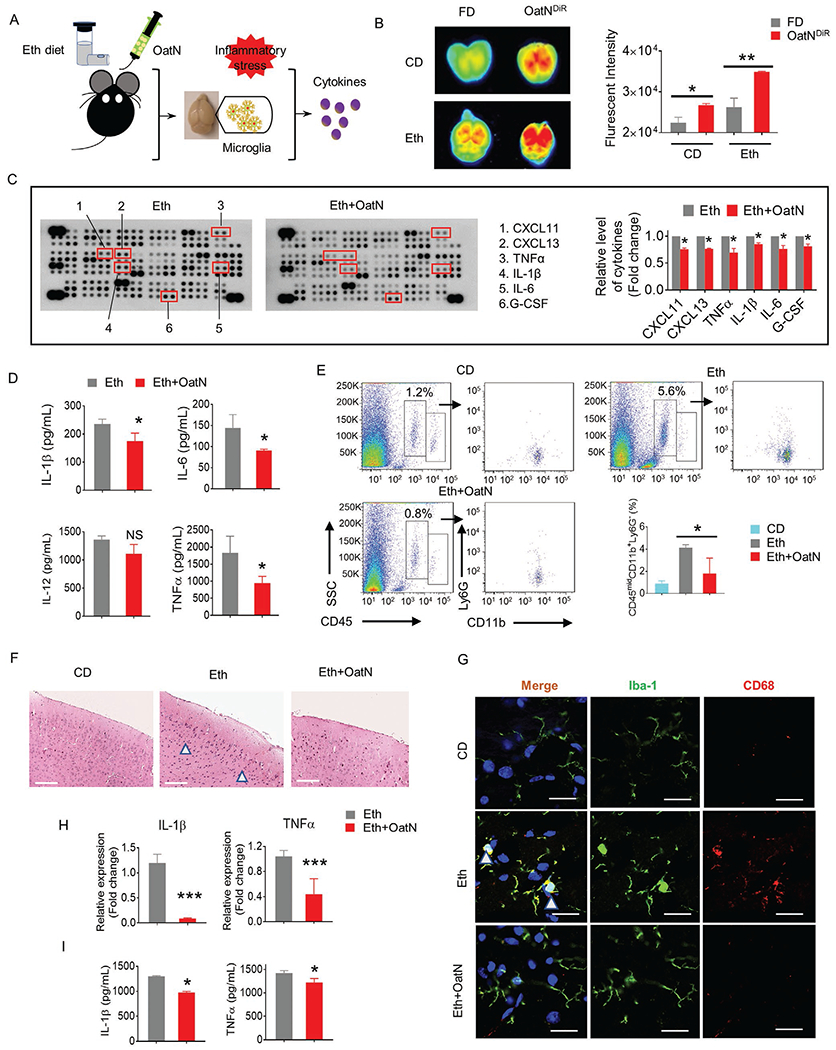
OatN inhibits alcohol induced brain inflammation. A) Schematic representation of the biological effects of oatN on the microglia cells in alcohol-induced chronic mouse brain inflammation. B) Mice were fed a control liquid diet (CD) or CD containing 5% ethanol (Eth) while being gavage-given oatN (8 mg kg^−1^ of body weight, three times per week) for 28 days. On the day before euthanasia, mice (*n* = 3 mice per group) were gavage-given DiR free dye (FD), or DiR labeled oatN (OatNDiR). Four hours after the gavage, the brain was excised and scanned with an Odyssey imager (left), and fluorescence intensity was quantitatively analyzed (right). Bar graphs represent the mean of imaging intensity from 3 mice/group and SEM (*n* = 3 mice/group). *p < 0.05, **p < 0.01 by ANOVA test. C) Mice (*n* = 5) were fed a control liquid diet (CD) or CD containing 5% ethanol (Eth) while being gavage-given oatN (8 mg/kg/mouse) three times per week for 28 days. The mouse cytokine array shows the signal intensity of each cytokine from the mice brain cerebrum lysates (left and middle, *n* = 3). Dot intensity was quantified and normalized with the ethanol group. Data represent ± mean SEM (right panel). *p*-values were calculated by means of Student’s *t*-test. *p < 0.05. D) Mice (*n* = 5) were fed a control liquid diet (CD) or CD containing 5% ethanol (Eth) while being gavage-given oatN (8 mg/kg/mouse) three times per week for 28 days. The inflammatory cytokine levels of IL-1*β*, IL-6, IL-12, and TNF*α* in the cerebrum were quantitatively analyzed with an ELISA. Data represent the mean ± SEM. *p*-values were calculated by means of Student’s *t*-test. *p < 0.05, NS, not statistically significant. E) Mouse (*n* = 5) microglial cells were isolated as described in Experimental Section. The percentage of CD45midCD11b+Ly6G− cell population in the brain cerebrum of mice fed with control liquid diet (CD) or CD containing 5% ethanol (Eth) while being gavage-given oatN (8 mg kg^−1^ of body weight) three times per week for 28 days was determined by flow cytometry. Representative cell staining and quantification of microglia cells in cerebrum. Data represent the mean (% of parent gate) ± SEM. *p*-values were calculated by means of ANOVA test. *p < 0.05. F) H&E-stained sections of brain cerebrum from mice treated with CD, Eth, or Eth plus oatN. Arrows indicate inflammation in the cerebrum. Scale bars, 70 mm. G) Mice were fed a control liquid diet (CD) or CD containing 5% ethanol (Eth) while gavage-given oatN (8 mg kg^−1^ of body weight) three times per week for 28 days. Brain cryostat sections were stained with anti-Iba-1 marker (green), anti-CD68 (red), and DAPI (blue), a confocal imaging assay was performed, and representative images were photographed (*n* = 5). Scale bars, 20 mm. H,I) Inflammatory cytokine expression in BV2 cells treated with Eth (200 mm) and Eth plus oatN (20 mg mL^−1^) in transcriptional level (H) and translational level (I) was determined using RT-PCR and ELISA, respectively. *N* = 3, data represent mean ± SEM. *p*-values were calculated by means of the Student’s *t*-test. *p < 0.05, ***p < 0.001.

Microglial cells are a specialized population of macrophages. They play an important role in immunomodulation for maintaining the health of the CNS. One of the hallmarks of alcohol use disorder (AUD) is chronic brain inflammation. Alcohol exposure induces brain inflammatory reactions via activation of microglial cells.^[13,34]^ Next, we analyzed the biological effects of oatN on the microglial cells in an alcohol-induced chronic mouse brain inflammation model (Figure 2A). OatN trafficked to the brain and chronic consumption of alcohol enhanced the uptake of oatN (Figure 2B). Cytokine array analysis results indicate that the level of the inflammatory cytokines (CXCL-11, CXCL-13, TNF*α*, IL-1*β*, IL-6, and G-CSF) was reduced in the cerebrum region of C57BL/6 mice administrated an alcohol liquid diet plus oatN in comparison with the mice treated with an alcohol liquid diet alone (Figure 2C). ELISA results further confirmed that oatN treatment significantly decreased the level of interleukin IL-6, IL-1*β*, and TNF*α* (Figure 2D). In addition, flow cytometry analysis showed that oatN treatment inhibited the induction of CD45^mid^CD11b^+^Ly6G^−^ microglia cells (Figure 2E). In comparison with the ethanol liquid diet treatment, H&E stained sections of brain cerebrum (Figure 2F) and hippocampus of mice treated with oatN (Figure S3D, Supporting Information) showed that oatN treatment led to the reduction of infiltrating cells, and confocal staining results indicate that activated microglial cells (CD68^+^Iba-1^+^) were decreased (Figure 2G). These results were further confirmed in an in vitro cell culture model. Microglial BV2 cells were used to determine the effect of oatN on the activation of BV2 cells. After ethanol treatment in the present or absent of oatN, the expression of IL-1*β* and TNF*α* were assessed by RT-PCR and ELISA, respectively. As shown in Figure 2H and 2I, oatN treatment significantly inhibited the activation of BV2 cells as indicated by reduction of the level of IL-1*β* and TNF*α*.

The oatN-mediated inhibition of expression of IL-1*β*, IL-6, and TNF*α* was also demonstrated in the liver (Figure S3E, Supporting Information) which is the primary site of alcohol metabolism and the organ for uptake of oatN. Collectively, these data suggest that oatN inhibits brain and liver inflammation.

### 2.3. OatN Protects Neuro Cells from Microglial Cells Mediated Brain Damage and Improves Mouse Memory

**Figure 3.**
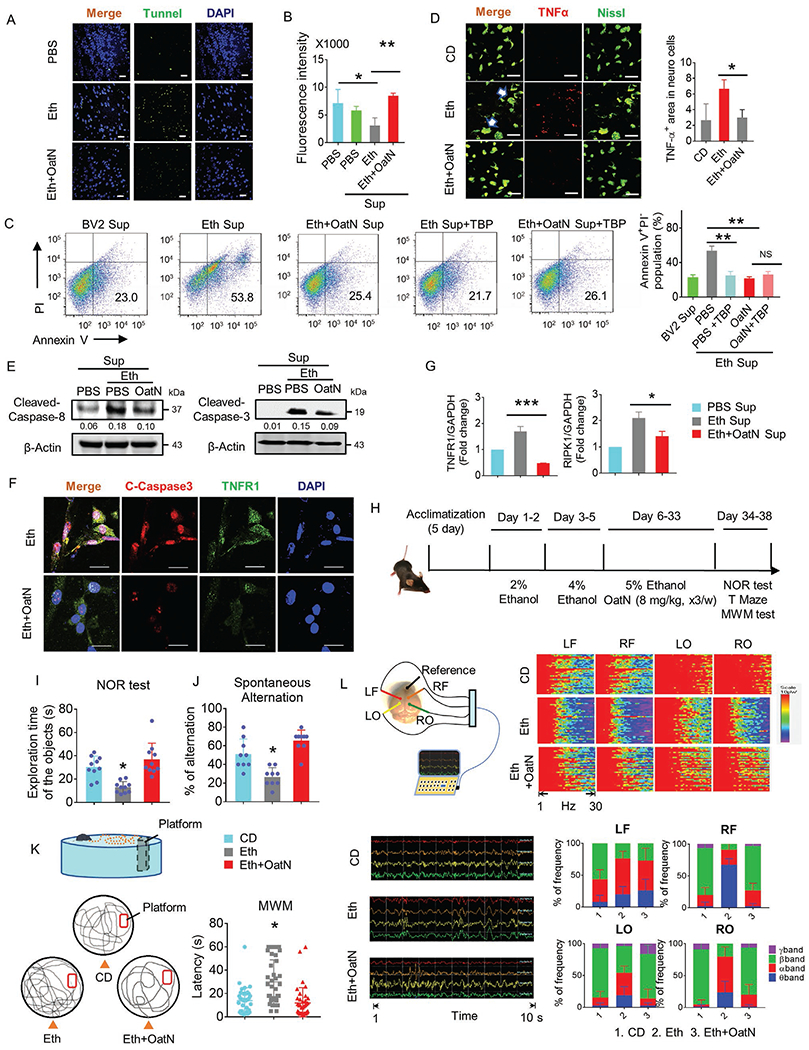
OatN protects neuro cells from microglial cells mediated brain damage and improves mouse memory. A) Mice were fed a control liquid diet (CD) or CD containing 5% ethanol (Eth) while being gavage-given oatN (8 mg kg^−1^ of body weight) three times per week for 28 days, mouse (*n* = 3 per group) cryostat sections were cut and stained using TUNEL staining kit. The slides were viewed by confocal microscope (TUNEL, green, DAPI, blue). Five random fields were photographed and representative results were shown. Scale bars, 20 mm. B) Primary neuro cells were isolated from C57B/6 mice (*n* = 5) and incubated with the supernatants from 24 h cultured BV2 cells (Sup) treated with ethanol (200 mm, Eth Sup), ethanol plus oatN (20 mg mL^−1^, Eth+OatN Sup) or PBS as a control. Neuro cell viability was determined using the ATPlite Luminescence Kit (PerkinElmer). *N* = 3, data represent mean of fluorescent intensity ± SEM. *p*-values were calculated by the means of ANOVA test. *p < 0.05, **p < 0.01. C) Primary neuro cells were isolated from C57B/6 mice (*n* = 5) and incubated with the supernatants from 24 h cultured BV2 cells treated with Eth (200 mm), Eth plus TNF*α* binding protein (TBP, 5 mg mL^−1^), Eth plus oatN (20 mg mL^−1^), or Eth plus oatN Sup and TBP. Percentages of apoptotic neuro cells (PI^+^AnnexinV^+^) were visualized and analyzed by flow cytometry. Flow cytometry analysis shows the % of apoptotic neuro cells in the bar graph (right panel). Data represent mean ± SEM. *p*-values were calculated by the means of an ANOVA test. **p < 0.01, NS, not statistically significant. D) Brain frozen sections from mice (*n* = 5 per group) treated with control diet (CD), ethanol diet (Eth), or Eth plus oatN were stained with an anti-neuro cell marker (Nissl, green) and anti-TNF*α* (red). Sections were examined by fluorescence microscopy and representative pictures were shown (*n* = 5). Arrow indicates of TNF*α*+ neuro cells. Bar graphs shows the mean number of TNF*α*+ neuro cells. Data represent mean SEM (*n* = 5). *p*-values were calculated by the means of Student’s *t*-test. *p < 0.05, scale bars, 20 mm. E) Total expression of cleaved caspase 3/8 of neuro cells treated 24 h with PBS Sup, Eth (200 mm) Sup or Eth plus oatN (20 mg mL^−1^) Sup from BV2 cells were analyzed by western blot. Band intensity was quantified and normalized by comparison to *β*-actin and the results are presented between the panels. F) Neuro cells were treated with Eth Sup or Eth plus oatN (20 mg mL^−1^) from BV2 cells for 24 h, and then fixed and stained with cleaved caspase-3 (red), TNFR1 (green), and DAPI (blue). Protein distribution and expression were visualized using confocal microscopy. Five random fields were photographed, and representative results (*n* = 3) are shown. Scale bars, 20 mm. G) Primary cultured neuro cells were treated with PBS Sup, Eth (200 mm) Sup, or Eth plus oatN (20 mg mL^−1^) from BV2 cells for 24 h, and TNFR1 and RIPK1 expressed in neuro cells was determined using an RT-PCR assay. Bar graphs represent the expression of TNFR1 and RIPK1 normalized to GAPDH (*n* = 5). *p < 0.05 and ***p < 0.001 by ANOVA test. H) Schematic representation of the oatN treatment schedule in the chronic alcohol consumption model. I) Mice were fed a control liquid diet (CD) or CD containing 5% ethanol (Eth) while being gavage-given oatN (8 mg kg1 of body weight) three times per week for 28 days. Mouse (*n* = 10 per group) behavior was evaluated using the novel object recognition (NOR) test. The time that mice spent with the discovery of the familiar and the new object was recorded. Data represent mean ± SEM. *p*-values were calculated by means of an ANOVA test. *p < 0.05. J) Mice were fed a control liquid diet (CD) or CD containing 5% ethanol (Eth) while being gavage-given oatN (8 mg kg^−1^ of body weight) three times per week for 28 days, mouse (*n* = 10 per group) behavior was evaluated by T-Maze test. Forced Alternation (%) was defined as described in Experimental Section. Data represent mean ± SEM. *p*-values were calculated by means of ANOVA test. *p < 0.05. K) Mice were fed with control liquid diet (CD) or CD containing 5% ethanol (Eth) while gavaging-given oatN (8 mg kg^−1^ of body weight) three times per week for 28 days, mouse (*n* = 10 per group) behavior was evaluated using the hidden platform Morris Water Maze (MWM) test (*n* = 15). Left figures represent one of the 15 tests. Data represent mean ± SEM. *p*-values were calculated by means of an ANOVA test. *p < 0.05. L) Mice (*n* = 5 per group) were fed a control liquid diet (CD) or CD containing 5% ethanol (Eth) while being gavage-given oatN (8 mg kg^−1^ of body weight) three times per week for 28 days, mouse (*n* = 5 per group). Representative images of four points in the mice brains (LF, left frontal, RF, right frontal, LO, left occipital, RO, right occipital) showed EEG record (bottom Left), EEG band power (upper right) and band frequency.

One of the pathological features of alcohol abuse is apoptosis of neuro cells.^[35]^ The results of TUNEL staining of brain cerebrum tissue demonstrated that oatN treatment led to a reduction in the number of apoptotic cells in the brain in comparison with alcohol treatment alone (Figure 3A). Whether oatN treatment has a direct or indirect effect on the prevention of alcohol-induced apoptosis of neuron cells was further studied. PKH26 labeled oatN was gavage-given to mice, 4 h after gavage, the mice were sacrificed. Our in vitro and in vivo data indicated that negligible numbers of oatN were taken up by neuro cells, while the majority of oatN were taken up by microglial cells (Figure 1G; Figure S4A,B, Supporting Information).

Central nervous system (CNS) function is supported by neuron-glia crosstalk. Excessive glial activation often occurs in neurodegeneration and is a cause of neuronal loss, which, in turn, establishes a state of chronic neuroinflammation.^[36–39]^ We hypothesized that neuronal damage is associated with the activation of microglia and oatN-mediated protection neuron cell death is via modulation of microglial cell activity. Primary neuro cells isolated from C57BL/6 mice were isolated and cultured with the supernatants from cultured microglial cells which had been treated for 24 h with or without oatN (20 mg mL^−1^) in the presence of ethanol (200 mm). The ATPlite assay showed that the supernatant from microglial cells treated with ethanol reduced the viability of the neuro cells (Figure 3B). Furthermore, the supernatant from microglial cells treated with ethanol increased the percent of apoptosis of neuron cells evaluated by co-staining with annexin V and PI, while the supernatant from ethanol plus oatN treated microglial cells did not (Figure 3C).

A number of factors including TNF*α* released from microglial cells may cause neuron cell apoptosis. Immunostaining of Nissl (neuron cell marker) and TNF*α* indicate that oatN inhibited the expression of TNF*α* protein in the brain (Figure 3D). To determine whether TNF*α* plays a causative role in neuron cell apoptosis induced by the supernatants released from microglial cells, TNF*α* binding protein (TBP)^[40]^ was added to neuron cell cultures to neutralize TNF*α* present in the microglial cell supernatant. Flow cytometry analysis of Annexin V positive apoptosis of neuron cells indicated that addition of TBP prevented neuro cell apoptosis induced by the supernatant collected from cultured microglial cells treated with ethanol (Figure 3C), suggesting that TNF*α* plays a role in the induction of neuron cell apoptosis. Activation of caspase-3 and caspase-8 has been reported in TNF*α* mediated apoptosis.^[41]^ Our results indicate that caspase-8 and caspase-3 were activated in neuro cells treated with the supernatants derived from microglial cells treated with ethanol (Figure 3E,F). This induction of activation was abrogated when neuro cells treated with the supernatants derived from microglial cells were treated with oatN plus ethanol (Figure 3E,F). Although no significant change was found in FADD activation, interestingly, PCR analysis indicated that oatN treatment inhibited the expression of TNFR1 and RIPK1 in the neuron cells stimulated with the supernatants derived from ethanol treated microglial cells (Figure 3G; Figure S4C, Supporting Information). Furthermore, less TNFR1 was available for TNF*α* mediated apoptosis of neuron cells, implying that oatN protected neuro cells from apoptosis via inhibition of expression of TNF*α* in the recipient microglial cells and TNFR1 expression in neuron cells (Figure S4C, Supporting Information). Neurons-glia crosstalk modulates neural activity. Alcohol abuse causes cognitive dysfunction, therefore, we determined whether oatN treatment can improve mouse memory as analyzed by the NOR, T maze and Morris-water maze (MWM) tests (Figure 3H). Interestingly, oatN-treated mice exhibited improved working and spatial memory in these behavioral tests (Figure 3I–K). To demonstrate the potential effect of alcohol and oatN on the of brain cerebrum function, a mouse epidural EEG from multiple cortical surface loci in the mice was recorded using a modified method.^[42]^ We found the incoherence of rhythms in the EEG patterns that are captured from 5% ethanol administrated mice while receiving oatN reversed the incoherence of rhythms (Figure 3L bottom left panel). Chronic ethanol liquid diet treated mice exhibited greater frequency in the theta and alpha bands in all four regions of the brain (Left frontal-LF, Right frontal-RF, Left occipital-LO, and Right occipital-RO), but lower frequency in the beta band compared to control diet treated mice (Figure 3L, bottom right panel). Also, suppression of the beta band density spectral assay (DSA) power was consistently found among the four sample regions of mice treated with alcohol (Figure 3L, upper right panel). However, oatN administration induced the DSA power in all four regions of the brain from alcohol treated mice (Figure 3L).

### 2.4. HPCA is Required for OatN Uptake by Microglial Cells and Inhibits Alcohol-Induced Expression of Inflammatory Cytokines Released from Microglial Cells via Interaction with Rab11a

**Figure 4.**
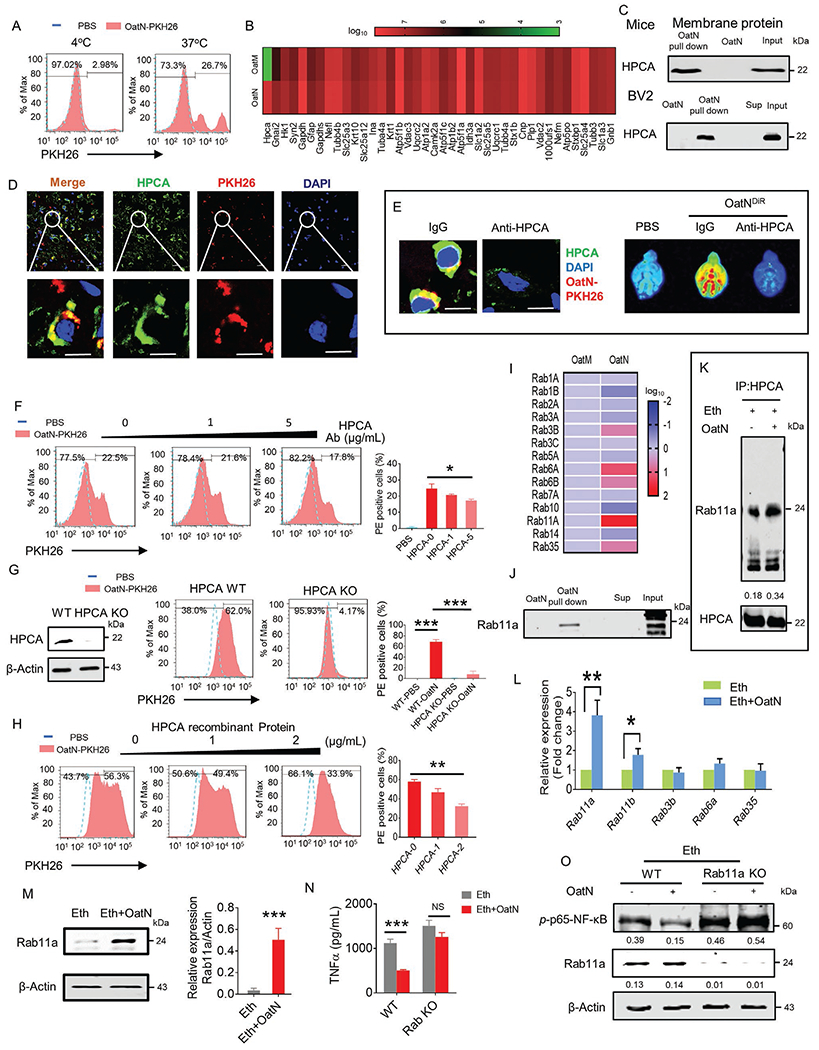
HPCA is required for OatN up-take by microglial cells and inhibits the expression of inflammatory cytokines released from microglial cells via an interaction with Rab11a. A) PKH26 labeled oatN (20 mg mL^−1^) was added to BV2 cell medium. Cell plates were incubated at 4 °C or 37 °C for 1 h. Cells were harvested, and fluorescence activated cell sorting (FACS) was performed to determine the PKH26+ cells. Flow cytometry analysis shows the % of PKH26+ cells and PKH26− cells (inset). B) Mouse microglial cell were isolated and lysed as described in Experimental Section. Lysate proteins were pulled down with oatN or oatM and analyzed on a MS/MS. The heat map plot shows comparative analysis of the top 50 most abundant proteins interacting with oatN versus oatM from the microglial cells isolated from mice. Green (low interaction) and red (high interaction). C) Mouse brain cerebrum membrane proteins (top panel) or BV2 cell membrane protein (bottom panel) were isolated. After pulling down proteins with oatN, the particles were washed and resuspend in RIPA lysis buffer. Western blot was performed to confirm HPCA binding to oatN. Results represent 1 out of 3 independent experiments. Input (left columns, top and bottom panels, total cell membrane protein lysates from before pulldown). D) Mice (*n* = 3) were gavage-given PKH26-labeled oatN (red, 8 mg kg^−1^ of body weight). 4 h after gavaging mice, the brains were excised, fixed, and stained with anti-HPCA (green) and DAPI (blue). Protein interaction and overlay were visualized using confocal microscopy. Five random fields were photographed, and representative results were shown. Scale bars, 10 mm (top), 20 mm (bottom). E) Representative brain images (*n* = 5, right panel) of mice gavage-given DiR-labeled oatN (8 mg kg^−1^ of body weight) after injection with IgG or anti-HPCA (3 μg per mouse) for 48 h. The confocal assay was performed to detect HPCA expression (green) after injection of IgG or anti-HPCA (left panel). Scale bars, 20 mm. F) BV2 cells were preincubated with varying concentration of anti-HPCA antibody for 8 h at 37 °C. PKH26 labeled oatN (20 mg mL^−1^) was then added to cell medium for 2 h and then percentages of PKH26+-oatN taken up by BV2 cells was determined by flow cytometry. Bar graphs represent the mean of FACS analysis results (*n* = 5). *p*-values were calculated by means of an ANOVA test. **p < 0.05. G) BV2 cells were infected with CRISPR/Cas9 HPCA knockout lentivirus as described in Experimental Section. HPCA knockout efficiency was confirmed by western blot (wild-type (WT) versus HPCA knockout (KO), left panel). OatN was labeled with PKH26 (20 mg mL^−1^) and added to the BV2 cell medium. PE-positive cells were quantitatively analyzed by FACS (middle panel). Bar graphs represent the mean of FACS analysis results (*n* = 5). *p*-values were calculated by means of an ANOVA test. ***p < 0.001. H) OatN was coated with the indicated concentration of HPCA recombinant protein at 37 °C for 1 h. OatN was then labeled with PKH26 (20 mg mL^−1^) and added to BV2 cell medium. PE-positive cells were quantitatively analyzed by flow cytometry. Bar graphs represent the mean of FACS analysis results (*n* = 5). *p*-values were calculated by means of an ANOVA test. **p < 0.01. I,J) Using the same approach as described in Figure 4B, the heat map plot shows comparative analysis of oatN versus oatM interacting with Rab family proteins (fold change) from lysates of mouse microglial cells listed in Figure 1. Legend: Red (upregulated), dark blue (downregulated), and white (no modulation). Rab11a was pulled down by oatN and examined by western blot. Sup (cell membrane protein supernatants after oatN pulldown), input (total cell membrane protein lysates before oatN pulldown). K) Interaction of HPCA and Rab11a was confirmed by immunoprecipitation and compared between cerebrum lysates of mice treated Eth or Eth plus oatN. HPCA were pull down and then Rab11a were probed by western blot. Band intensity was quantified and normalized with HPCA and the results are presented between the panels. Rab11a expression was detected at the L) transcriptional and M) protein levels in brain cerebrum lysates of mice (*n* = 3) fed a control liquid diet (CD) or CD containing 5% ethanol (Eth) while being gavage-given oatN (8 mg kg^−1^ of body weight) three times per week for 28 days. Band intensity was quantified and normalized to *β*-actin. Data represent the mean ± SEM. *p*-values were calculated by means of the Student’s *t*-test. *p < 0.05, **p < 0.01. N) BV2 wild-type cells and Rab11a knockout cells were treated with Eth (200 mm) or Eth plus oatN (20 mg mL^−1^). TNF*α* level in cell medium was determined using an ELISA. Bar graphs represent the mean of ELISA analysis results (*n* = 5). *p*-values were calculated by means of an ANOVA test. ***p < 0.001, NS, not statistically significant. O) Representative western blot of p-p65-NF-*κ*B and Rab11a in BV2 wild type cells and Rab11a knockout cells treated with Eth (200 mm) or Eth plus oatN (20 mg mL^−1^). Band intensity was quantified and normalized to *β*-actin and the results are presented between the panels.

**Figure 5.**
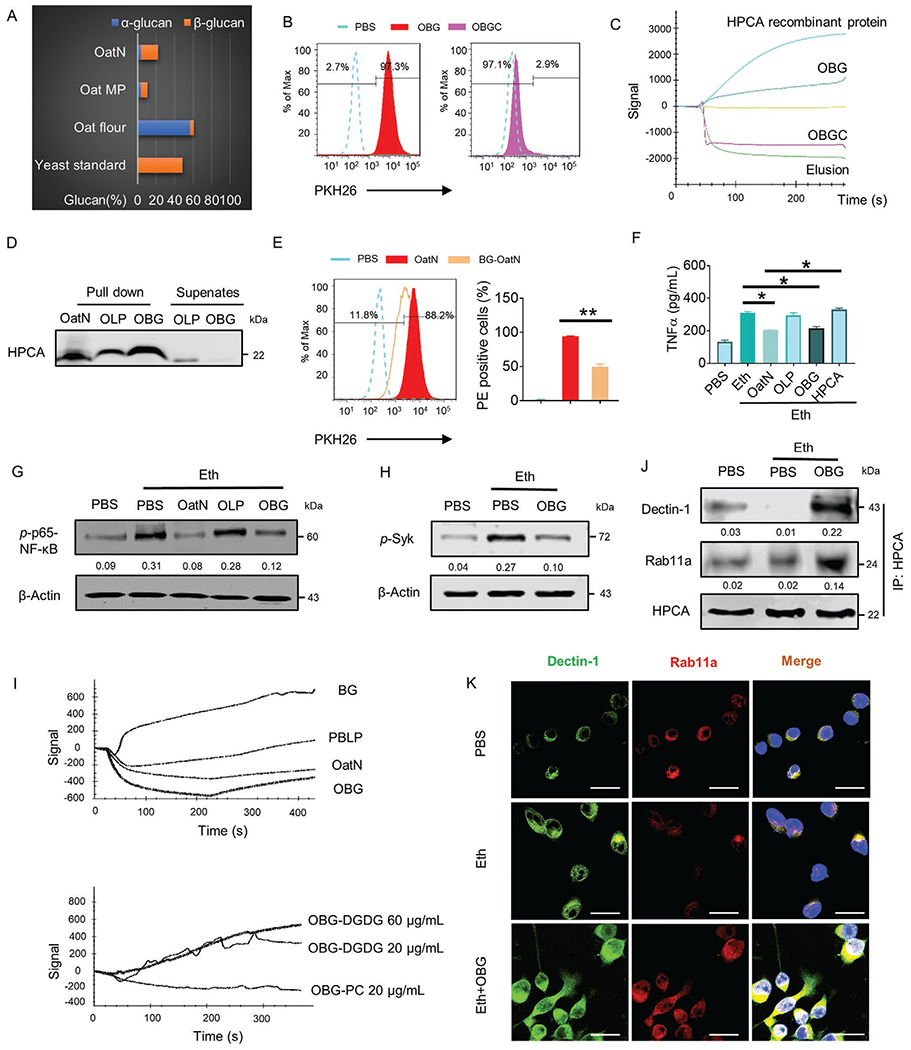
OatN *β*-glucan binds to HPCA, promotes microglia up-take of oatN and inhibits dectin-1 activation, whereas OatN digalactosyldiacylglycerol (DGDG) inhibits oatN *β*-glucan access to dectin-1. A) Total, *α*- and *β*-glucan in oatN, oatM, or oat flour were determined using *β*-glucan assay kit (Megazyme). Yeast *β*-glucan powder was used as a standard. Data were replicated in at least two independent experiments. B) OatN derived *β*-glucan and enzymatically (*β*-glucanase, 100 U mL^−1^) digested oatN derived *β*-glucan were packed into liposomes made from the total lipids extracted from oatN (OBG and OBGC). OBG and OBGC were labeled with PKH26 and then added to BV2 cell medium. PKH26-positive cells were quantitatively analyzed by FACS. Flow cytometry analysis shows the % of PKH26+ cells and PKH26− cells (Inset). C) The surface plasmon resonance (SPR) assay was performed to determine OBG binding to HPCA. The signal output is directly related to changes in binding capacity on the sensor surface. The five curves represent signals sequences: NTA sensor chip coated with HPCA-his protein (light blue, HPCA-his, 200 ng mL^−1^), after OBG injection (dark blue, OBG, 20 mg mL^−1^), before OBG injection (yellow, baseline), after OBG-chopped by *β*-glucanase (purple, OBGC, mg mL^−1^) injection and analyte elution (green, elusion). D) Mouse brain cerebrum (*n* = 5) membrane proteins were isolated. After being pulled down by oatN, particles were washed and resuspend in RIPA lysis buffer. Western blot was performed to compare binding capacity of HPCA to nanoparticles made from the total lipids extracted from oatN (OLP) or OBG. E) BV2 cells were blocked with commercially available *β*-glucan (BG, 100 mg mL1) before PKH26 labeled oatN (20 mg mL^−1^) was added to cell medium. PKH26-positive cells were quantitatively analyzed by FACS (right panel). Bar graphs represent the mean of FACS analysis results (*n* = 5). *p*-values were calculated by means of an ANOVA test. **p < 0.01. F) BV2 cells were treated with Eth (200 mm) or Eth plus oatN (20 mg mL^−1^), OLP, OBG, or HPCA protein (50 mg mL^−1^) coated oatN (HPCA) for 24 h, respectively. TNF*α* level in cell medium was determined using an ELISA. *N* = 3, data represent the mean ± SEM. *p*-values were calculated by means of the Student’s *t*-test. *p < 0.05. G) A representative western blot of p-p65-NF-*κ*B in BV2 cells treated with or without Eth (200 mm), Eth plus OatN (20 mg mL^−1^), OLP (20 mg mL^−1^), or OBG (20 mg mL^−1^). Results represent 1 out of 3 independent experiments. Band intensity was quantified and normalized to *β*-actin and the results are presented between the panels. H) A representative western blot of p-Syk in BV2 cells treated with Eth (200 mm) or Eth plus OBG (20 mg mL^−1^). Results represent 1 out of 3 independent experiments. Band intensity was quantified and normalized to *β*-actin and the results are presented between the panels. I) The surface plasmon resonance (SPR) assay was performed to determine OBG binding to dectin-1 . (Upper panel) BG (commercial *β*-glucan, 100 ng mL^−1^), PBLP (liposomes made by peripheral blood cell derived lipids), OatN (20 mg mL^−1^), and OBG (20 mg mL^−1^) were used as analytes. (Bottom panel) OBG-DGDG (OBG remove DGDG, 20 or 60 mg mL^−1^) and OBG-PC (OBG remove PC, 20 mg mL^−1^) were used as analytes. Results represent 1 out of 3 independent experiments. J) Dectin-1 binding to HPCA-Rab11a complex was detected by immunoprecipitation. HPCA complex in BV2 cells treated with Eth or OBG was pulled down by anti-HPCA antibody (1 mg mL^−1^). Dectin-1 and Rab11a were probed in a western blot. Results represent 1 out of 3 independent experiments. Band intensity was quantified and normalized by HPCA, and the results are presented between the panels. K) Confocal images of dectin-1 (green) overlay with Rab11a (red) in BV2 cells treated with Eth (200 mm) or Eth plus OBG (20 mg mL^−1^). Results represent 1 out of 3 independent experiments. Five random fields were photographed, and representative results are shown. Scale bars, 20 μm.

Microglial cells behaved as major oatN recipient cells, however, the underlying molecular mechanism explaining the trafficking pattern was unclear. First, we determined whether energy is required for uptake of the oatN. To address this aspect, PKH26 dye labeled oatN was co-cultured with the BV2 cell line at 4 and 37 °C. Fluorescence activated cell sorting (FACS) analysis results indicate that a much higher percentage of PKH26^+^ positive cells were observed in cells incubated at 37 °C than at 4 °C (Figure 4A) suggesting that oatN uptake by microglial cells requires active transportation. Next, the proteins from murine microglial cells, pulled down by oatN in vitro and identified using Tandem mass spectrometry (MS/MS) by comparison to a mammalian database were analyzed to identify the microglial factor(s) that interact with oatN (Figure 4B; Table S4, Supporting Information). Comparison of oat microparticle (oatM) pulled down proteins with oatN pulled down protein indicated that oatN bound overwhelmingly higher amounts of hippocalcin (HPCA). The oatN-pulled down protein interaction was confirmed by western blot analysis of HPCA present in murine microglial cells as well as the BV2 cell line derived membrane proteins (Figure 4C). Also, co-localization of oatN and HPCA were visualized on brain cerebrum frozen sections (Figure 4D). To assess the critical role of brain HPCA in terms of oatN uptake, mice were injected intracranially with HPCA antibody using a previously described protocol and with a normal IgG used as a control.^[43]^ Notably, pre-injection of anti-HPCA antibody inhibited oatN uptake in the brain (Figure 4E), suggesting that microglial HPCA plays a role in oatN uptake. In vitro analysis was done using an FACS method which showed that pre-incubation of BV2 cells with anti-HPCA antibody inhibited the uptake PKH26 labeled oatN in an anti-HPCA antibody dose dependent manner (Figure 4F). The HPCA role in the uptake of oatN was further supported by the fact that specifically knocking out or inducing HPCA expression in BV2 cells transfected with hippocalcin CRISPR plasmid, respectively, abolished or promoted the entry of oatN into the BV2 cells (Figure 4G; Figure S5A, Supporting Information). Moreover, oatN pre-incubated with HPCA recombinant protein led to inhibition of oatN entry into BV2 cells in an HPCA protein concentration dependent manner (Figure 4H). Taken together, these data suggest that microglial HPCA is required for oatN entry. ELISA results showed that oatN preincubated with HPCA protein had no effect on the inhibition of TNF*α* being induced by ethanol (Figure 5F), suggesting that HPCA not only plays a role in oatN entry but also inhibition of TNF*α* expression.

In addition to our findings regarding HPCA, we noticed that Rab11a is also enriched in the oatN pulldown complex (Figure 4I). MS/MS analysis results were further confirmed by western blot analysis of Rab11a (Figure 4J). Since Rab11a and HPCA are in the oatN complex, whether HPCA interacted with Rab11a is unknown. Our HPCA pulldown followed by western blot analysis of Rab11a indicate that Rab11a does interact with HPCA upon oatN stimulation (Figure 4K). Next, we determined whether oatN has an effect on the expression of Rab11a and HPCA in microglial cells (Figure S5B, Supporting Information) or in the brain of mice (Figure S5C, Supporting Information) treated with an ethanol diet. Western blot analysis indicated that oatN appears to have no effect on the level of HPCA protein (Figure S5B,C, Supporting Information). However, expression of Rab11a at both the mRNA (Figure 4L) and protein level (Figure 4M; Figure S5D, Supporting Information) was increased in the brain of alcohol fed mice gavage-given oatN.

Next, we determined whether Rab11a participated in oatN mediated inhibition of inflammatory cytokine expression in microglial cells. Since knockout (KO) of Rab11a is lethal, the BV2 cell line was used to study the effect of Rab11a on the activation of mouse microglial cells. Rab11a was knocked out in BV2 cells (Figure S5E, Supporting Information), and cells were then treated with ethanol in the present/absent of oatN. The expression of TNF*α* and activation of NF-*κ*B were assessed by ELISA and western blot, respectively. OatN treatment significantly inhibited the alcohol induced BV2 activation as indicated by a reduction in the level of TNF*α* and Rab11a KO cancelled the beneficial effect (Figure 4N,O).

### 2.5. OatN *β*-Glucan Binds to HPCA, Promotes the Microglia Uptake of OatN and Inhibits Dectin-1 Activation, Whereas OatN Digalactosyldiacylglycerol (DGDG) Inhibits OatN *β*-Glucan Access to Dectin-1

Next, we sought to identify the oatN-derived factors that contributed to the phenotypes induced by oatN in microglial cells that were involved in the inhibition of the inflammatory pathway. *β*-glucan has been shown to modulate the expression of cytokines. Oat *β*-glucan (BG) is reported as the main beneficially active component for cardiovascular disease.^[44]^ We found that BG was enriched, from 4% in flour to nearly 20% in oatN (Figure 5A). The role of BG extracted from oatN in microglial cell uptake was further demonstrated. Addition of oatN derived BG to the nanoparticles made from the total lipids (OBG) extracted from oatN (OLP) promoted the uptake of OBG in a BG does dependent manner (Figure S6A, Supporting Information). Moreover, the bulk of the particles taken up by BV2 cells was reduced when BG was enzymatically digested with beta-glucanase (Figure 5B), suggesting that BG plays a role in microglial cell uptake of OBG.

We have demonstrated that host HPCA protein is required for oatN entry into microglial cells (Figure 4F–H). Whether oatN derived BG directly binds to HPCA for the oatN entry was further determined. We utilized the surface plasmon resonance (SPR) technique to determine whether HPCA binds to BG. HPCA protein was coated on the protein sensor chip. OBG was used as analyte and run over the sensor chip. The results showed that OBG binds to HPCA while beta-glucanase chopped OBG did not (Figure 5C). We further confirmed HPCA interacting with OBG using an OBG pull-down assay, followed by a western blot for HPCA. The result indicates that OBG interacts more HPCA protein than the control liposomes made from the total lipids extracted from oatN (OLP) (Figure 5D). To prove BG plays a critical role in the uptake of oatN, we blocked HPCA on the surface of BV2 cells with commercial oat *β*-glucan prior to adding PKH26 labeled oatN. FACS data indicated that the ability of oatN to enter cells was significantly inhibited (Figure 5E).

A number of pathways could contribute to microglial cell uptake of oatN, therefore, we sought to determine the specific pathway essential for oatN uptake. BV2 cells were incubated for 1 h with PKH26 labeled oatN in the present of inhibitors including amiloride (1 mm, macropinocytosis), chlorpromazine (20 μm, clathrin-related endocytosis), and Filipin (5 μg mL^−1^, caveolin-mediated endocytosis). FACS results for PKH26 positive cells analyzed (Figure S6B, Supporting Information) indicated that the majority of oatN was taken up via macropinocytosis.

Next, we determined whether OBG plays a role in prevention of microglial cell activation induced by alcohol. BV2 cells were treated with OBG and oatN in the presence of ethanol for 24 h. OLP served as a control (also in the presence of ethanol for 24 h). After the incubation period, TNF*α* in the cultured supernatants were quantified and phosphorylation of p65-NF-*κ*B was determined by western blot. The results suggests that OBG inhibited alcohol induction of TNF*α* (Figure 5F), and phosphorylation of p65-NF-*κ*B (Figure 5G).

BG is a well-known factor that binds to and activates dectin-1. Dectin-1 recognizes various danger-associated molecular patterns (DAMPs) and triggers inflammatory signals by recruiting its downstream molecular tyrosine kinase, Syk.^[45]^ Our results show that ethanol induced the phosphorylation of Syk of BV2 cells treated with PBS (Figure 5H). Interestingly, OBG treatment inhibited the phosphorylation of Syk induced by ethanol.

Next, the role of dectin-1 in the context of alcohol mediated activation of NF-*κ*B, Syk, and induction of TNF*α* was further investigated by orally giving laminarin or PBS as a control while mice were being provided with a 5% ethanol liquid diet or control liquid diet. Laminarin, a water-soluble *β*-glucan is a dectin-1 antagonist that binds to dectin-1 without stimulating downstream signaling.^[46,47]^ Twenty-eight days after mice were put on an ethanol diet, they were treated with laminarin (300 mg kg^−1^ of body weight), brain tissue lysates and the supernatants extracted from brain tissue of treated mice were analyzed. The results indicate that the alcohol stimulated activation of NF-*κ*B, Syk, and induction of TNF*α* compared with results from mice fed the control liquid diet. Laminarin treatment reversed the effect of alcohol, suggesting that dectin-1 contributes to the alcohol mediated activation of NF-*κ*B, Syk, and induction of TNF*α* (Figure S6C,D, Supporting Information). The results generated from the mouse model were reproduced in BV2 cell cultures (Figure S6E,F, Supporting Information).

Collectively, these results prompted us to further explore the molecular mechanism underlying the reason why oatN *β*-glucan preferentially binds to HPCA but not dectin-1 which is partially located within the cytoplasmic membrane. We noticed that OBG stimulated the expression of Rab11a and inhibited the expression of TNF*α* (Figure S6G,H, Supporting Information). However, BG which does not contain oatN lipids induced the expression of TNF*α* protein, suggesting that oatN lipid may prevent oatN *β*-glucan from accessing plasma membrane dectin-1. Therefore, we utilized the SPR technique to determine whether oatN lipid inhibits oatN BG binding to dectin-1. Based on the oatN lipid profile (Table S1, Supporting Information), phosphatidylcholine (PC) (30%) and digalactosyldiacylglycerol (DGDG) (29.8%) are the two most predominate lipids in oatN. To determine whether PC and DGDG inhibit *β*-glucan binding to dectin-1, dectin-1 protein was coated on the SPR protein sensor chip. BG carried by nanoparticles made from oatN total lipid with depletion of PC or DGDG were used as analyte to run over the sensor chip. In addition, OBG, oatN, or nanoparticles made from the total lipid extracted from B6 mice peripheral blood cells (liposomes) were used as controls. OBG, oatN, or the control nanoparticles did not bind to dectin-1 (Figure 5I upper panel). The nanoparticles with depletion of PC did not bind to dectin-1, whereas nanoparticles with depletion of DGDG bound to decin-1 (Figure 5I bottom panel), suggesting that DGDG inhibits *β*-glucan binding to dectin-1. OBG with depletion of DGDG also induced p65-NF-*κ*B as well as p-Syk and induction of TNF*α* in BV2 cells treated with ethanol (Figure S6I,J, Supporting Information).

The fact that oatN *β*-glucan preferentially binds to HPCA but not dectin-1 was further confirmed by confocal imaging data. BV2 cells pretreated with/without *β*-glucanase were incubated for 1 h with PKH26 labeled oatN in the present of amiloride (1 mm). The treated cells were fixed and, subsequently stained with dectin-1 or HPCA for confocal microscopic analysis. The results suggest that HPCA but not dectin-1 co-localized with oatN (Figure S6K, Supporting Information). OatN pretreated with *β*-glucanase resulted in no oatN co-localized with HPCA, suggesting that oatN *β*-glucan interacted with HPCA (Figure S6L, Supporting Information).

Once the oatN enters the microglial cell, *β*-glucan still binds to HPCA whereas DGDG is dropped from the HPCA pulldown complex (Figure S6M,N, Supporting Information). The role of HPCA complex was further demonstrated. Our results suggest that ethanol treatment inhibits the interaction of HPCA with Rab11a (Figure 5J) and reduced the expression of Rab11a (Figure S5D, Supporting Information) in BV2 cells. OBG enhanced the HPCA/dectin-1/Rab11a complex formation in BV2 cells (Figure 5J). Further, OBG induced Rab11a protein expression and enhanced reciprocal interaction of dectin-1 and Rab11a in BV2 cells (Figure 5K).

### 2.6. Rab11a Alters the Trafficking Routes of Dectin-1 in Microglial Cells Treated with OatN

**Figure 6.**
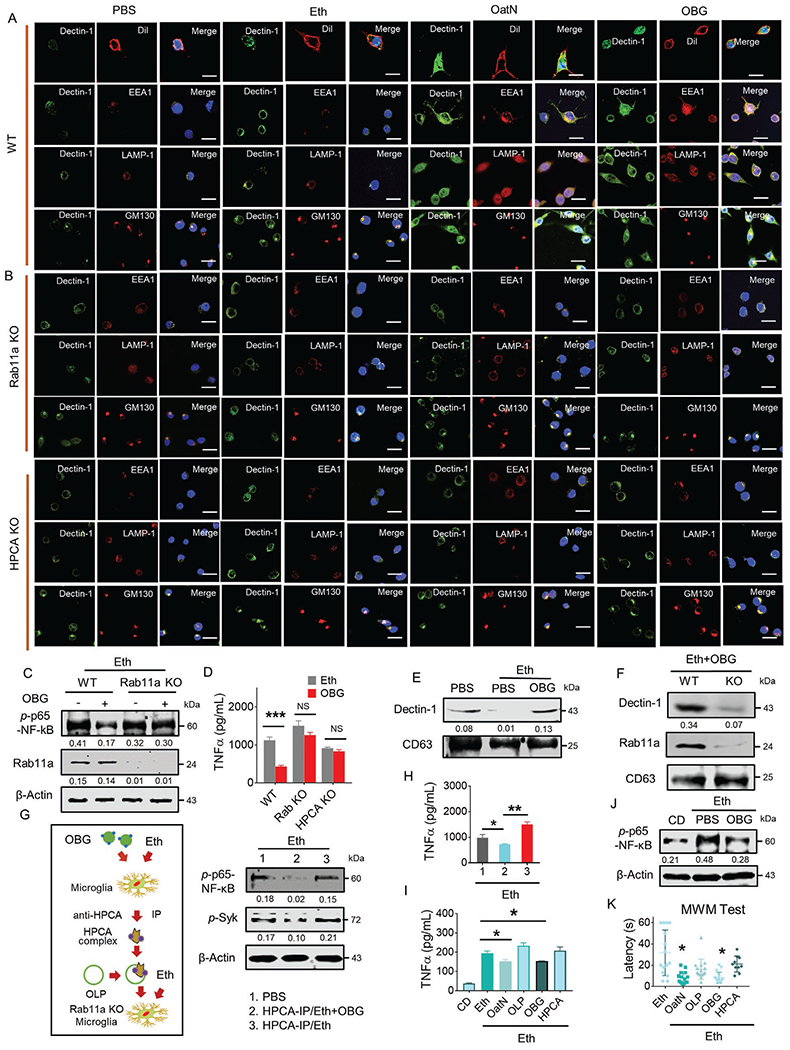
Rab11a alters the trafficking routes of dectin-1 in microglial cells treated with oatN. A) BV2 cells were treated with PBS, Eth (200 mm), Eth plus oatN (20 mg mL^−1^), or OBG (20 mg mL^−1^) for 12 h. Fluorescence images of microglial cells stained with Dectin-1 (green) were overlayed EEA1, LAMP-1, GM130, DiI (red), or DAPI (blue). Five random fields were photographed, and representative results were shown. Scale bars, 20 mm. B) BV2 cells with knockout of Rab11a or HPCA were treated with PBS, Eth (200 mm), Eth plus oatN (20 mg mL^−1^), or OBG (20 mg mL^−1^) for 12 h. Fluorescence images of microglial cells stained with Dectin-1 (green) were overlayed with EEA1, LAMP-1, GM130, or DAPI (blue). Five random fields were photographed, and representative results were shown. Scale bars, 20 mm. C) A representative western blot of p-p65-NF-*κ*B and Rab11a in BV2 wild type cells and Rab11a knockout cells treated Eth (200 mm) or Eth plus OBG (20 mg mL^−1^). Band intensity was quantified and normalized to *β*-actin and the results are presented between the panels. D) BV2 cells with/without knockout of Rab11a or HPCA (*n* = 3) were treated for 24 h with Eth (200 mm) or Eth plus OBG (20 mg mL^−1^). TNF*α* level in the cell medium was determined using an ELISA. Bar graphs represent the mean of ELISA analysis results (*n* = 5). *p*-values were calculated by means of an ANOVA test. *p < 0.05. E) A representative western blot of dectin-1 in exosomes isolated from cultured BV2 cells treated for 24 h with PBS, Eth (200 mm) or Eth plus OBG (20 mg mL^−1^). CD63 was used as loading control. Band intensity was quantified and normalized by CD63, and the results are presented between the panels. F) A representative western blot of dectin-1 in exosomes isolated from BV2 wild-type or Rab11a knockout cells treated with Eth (200 mm) or Eth plus OBG (20 mg mL^−1^). CD63 was used as a loading control. Band intensity was quantified and normalized by CD63 and the results are presented between the panels. HPCA complex in BV2 cells treated with Eth (200 mm) or OBG (20 mg mL^−1^) was pulled down and packed into OLP. Rab11a knockout BV2 cells were incubated for 24 h with Eth or Eth plus OLP packing HPCA complex (20 mg mL^−1^). Phosphorylation of NF-*κ*B was probed by western blot. G) Band intensity was quantified and normalized to *β*-actin and the results are presented between the panels. H) Cell supernatants were collected and the TNF*α* concentration was detected using an ELISA. Bar graphs represent the mean of ELISA results (*n* = 5). *p*-values were calculated by means of an ANOVA test. *p < 0.05, **p < 0.01. I) Mice (*n* = 5) were fed a control liquid diet (CD) or CD containing 5% ethanol (Eth) while being gavage-given oatN (8 mg kg^−1^ of body weight), OLP (8 mg kg^−1^ of body weight), OBG (8 mg kg^−1^ of body weight), or HPCA coated oatN (8 mg kg^−1^ of body weight), three times per week for 28 days. TNF*α* levels in brain cerebrum lysates were determined using an ELISA. Bar graphs represent the mean of ELISA analysis results (*n* = 5). *p*-values were calculated by means of ANOVA test. *p < 0.05. J) A representative western blot of p-Syk and p-p65-NF-*κ*B in mice fed a CD containing 5% ethanol (Eth) or Eth while being gavage-given OBG (8 mg kg^−1^ of body weight) three times per week for 28 days. Results represent 1 out of 3 independent experiments. Band intensity was quantified and normalized to *β*-actin and the results are presented between the panels. K) Mice (*n* = 10) were fed a CD containing 5% ethanol (Eth) or Eth plus gavage-given oatN, OLP, OBG, or HPCA coated oatN (8 mg kg^−1^ of body weight), three times per week for 28 days. Mouse behavior was evaluated using the hidden platform Morris Water Maze (MWM) test (*n* = 15). Data represent mean ± SEM. *p*-values were calculated by means of an ANOVA test, *p < 0.05.

Published data indicates that particulate BG binds to dectin-1,^[45]^ and our data in this study show that oatN OBG binds to HPCA which recruits Rab11a, and subsequently enhances interaction of Rab11a with intracellular dectin-1. Rab11a plays a crucial role in recycling of proteins from the trans-Golgi network (TGN) to the plasma membrane. Within membrane-trafficking routes, the small G protein Rab11 localizes at the TGN, post-Golgi vesicles of the secretory pathway, and the recycling endosome (RE). During recycling of cell membrane proteins, Rab11 functions in later transport steps from the early endosome (EE) and late endosome (LE)/multivesicular body (MVB) to the pericentriolar RE and back to the plasma membrane (late recycling). Dectin-1 is predominately located at the Golgi and cytoplasmic membrane.^[48]^ Whether oatN or OBG treatment alters the location of dectin-1 was further analyzed. Confocal imaging results indicate that ethanol stimulation increased dectin-1 signal detected in the cytoplasmic membrane (Figure 5K) and OBG plus alcohol treatment seems to have no effects on the level of cytoplasmic membrane associated dectin-1 (DiI^+^dectin-1^+^) but cytosol dectin-1 was increased (Figure 6A, the first panel). Next, we examined where dectin-1 is localized after the treatment. Confocal imaging results indicate that oatN or OBG treatment remarkedly enhanced the dectin-1 signals detected not only in Golgi, but also in early endosome and lysosome over time, starting from 60 min and reaching maximum signal that plateaued at 12 h (Figure 6A, panels 2–4 and Figure S7A, Supporting Information). KO of Rab11a or HPCA canceled the enhancing effects (Figure 6B), suggesting that Rab11a which is recruited by the oatN *β*-glucan/HPCA complex dictate the dectin-1 trafficking routes and therefore its downstream signaling pathways. This assumption was supported by the fact that when Rab11a or HPCA is knocked out of the BV2 cell line, there was no OBG mediated inhibition of ethanol induced *p*-p65-NF-*κ*B or *p*-Syk, and expression of TNF*α* significantly recovered (Figure 6C,D; Figure S7B,C, Supporting Information).

Exosomes are released from cells via multivesicular bodies (MVB). Golgi vesicles participate in MVB formation.^[49,50]^ As Rab11a also play a key role in the release of exosomes,^[51]^ we further proposed that Rab11a also acts as endosomal cargo to regulate the sorting of dectin-1 into exosomes. We observed that the level of exosomal dectin-1 was significantly increased when cells were treated with OBG (Figure 6E) and, KO of Rab11a abolished the OBG induced dectin-1 sorting into exosomes (Figure 6F). The results generated from the Nanosight NS300 analysis of PE-dectin-1-stained exosomes indicates that the dectin-1 was present on the surface of exosomes (Figure S7D,E, Supporting Information).

To determine whether Rab11a plays an essential role in OBG stimulated HPCA complex mediated inhibition of alcohol induced inflammation, we isolated this complex by HPCA pulldown of BV2 cells treated for 24 h with OBG and BG or PBS as controls in the presence of ethanol (200 mm). The western blot analysis results suggested that OBG induced HPCA/dectin-1/Rab11a complex formation (Figure 5J). Rab11a KO BV2 cells were treated with the oatN lipid derived nanoparticles carrying the HPCA/dectin-1/Rab11a complex in the presence of ethanol (Figure 6G, left panel). The results indicated that phosphorylation of NF-*κ*B and expression of TNF*α* induced by alcohol was inhibited by the HPCA complex of BV2 cells treated with OBG when compared to the HPCA complex isolated from BV2 cells treated with OBG or PBS (Figure 6G,H).

Collectively, our results show that oatN *β*-glucan binds to HPCA rather than dectin-1 via DGDG mediated inhibition of *β*-glucan access to dectin-1. Subsequently *β*-glucan/HPCA recruits Rab11a to the HPCA complex via interaction with HPCA, leading to formation of the oat *β*-glucan/HPCA/dectin-1/Rab11a complex. OBG stimulation further enriches the HPCA/dectin-1/Rab11a complex, and that inhibition of activation of NF-*κ*B, Syk, and induction of TNF*α* induced by alcohol are Rab11a dependent. Rab11a associated HPCA complex has at least two biological effects: 1) promoting translocation of dectin-1 from the Golgi to early endosomes and lysosomes and 2) recruitment of Rab11a in the complex triggers sorting of dectin-1 into exosomes, collectively leading to preventing alcohol mediated activation of the microglia inflammatory signaling pathway.

Next, we tested whether OBG plays a key role in oatN mediated inhibition of brain inflammation and improvement of brain memory of mice fed an ethanol liquid diet. Mice were treated with ethanol liquid diet plus OBG and compared with mice fed an ethanol plus oatN, OLP, OBG, or HPCA protein coated nanoparticles made from the total lipids extracted from oatN as controls. The results indicate that treatment of mice with OBG nanoparticles alleviated alcohol induced TNF*α* increase in brain cerebrum lysates as did oatN, in contrast the results generated from OLP and HPCA coated nanoparticles showed no inhibitory effects on TNF*α* induction (Figure 6I). We also found OBG treatment induced dectin-1 co-localized with Rab11a, EEA1, or LAMP prevented NF-*κ*B activation in vivo (Figure 6J; Figure S8A, Supporting Information). In addition, we observed an improvement in alcohol related memory damage when oatN and OBG were given to mice (Figure 6K), suggesting that oat BG plays a major role in preventing alcohol induced brain inflammation and improving brain memory function.

### 2.7. Discussion

Through a natural oral administration route, this study defines an edible plant nanoparticle-mediated mechanism that acts via microglia to inhibit alcohol induced brain inflammation and reestablished brain memory functions. As proof-of-concept, we demonstrate that oatN *β*-glucan binding to HPCA drives microglial cell engulfment of the oatN meanwhile oatN DGDG inhibits oatN *β*-glucan access to membrane dectin-1.

After endocytosis, *β*-glucan/HPCA subsequently recruits Rab11a and dectin-1 into the HPCA complex, leading to an altering of the dectin-1 trafficking route, revealing a molecular pathway through which alcohol induced microglial cell activation is inhibited and memory functions are recovered. The cellular and molecular mechanism(s) underlying the protective effect and the findings are summarized in the graphical abstract.

### 2.8. Edible Plant Nanoparticles Regulate Brain Function via Targeting Microglial Cells

Numerous studies indicate that the plant kingdom provides not only nutrients but also bioactive natural products to prevent/treat brain diseases that occur in the mammalian kingdom. How plant-derived bioactive natural products execute their functions on the mammalian brain while resisting the harsh GI environment, that is, the low pH in the stomach and the degradative enzymes. Recently, our data^[52–54]^ and others published^[55–58]^ indicate that depending on what types of nanoparticles, there are a variety of mechanisms underlying the resistance. It is also challenging to treat brain related disease via orally delivering therapeutic agents due to the fact that the agent must resist the harsh GI environment. Our data published^[52–54]^ and presented in this study show that edible plant-derived exosome-like nanoparticles are potential candidates to meet this challenge. Therefore, it is conceivable that oatN could be used as a therapeutic agent as well as a drug delivery vehicle to treat brain disease in a non-invasive manner by targeting microglial cells.

Finding that oral administration of oat derived nanoparticles leads to protection of mice from alcohol-induced brain inflammation not only suggests that oatN could be used as a novel agent to protect the brain against inflammation but provides a foundation for further studying the brain immune tolerance mechanism underlying interspecies communication through nanoparticles we ingest daily from many different types of edible plants. Furthermore, the finding that oral administration of edible plant derived nanoparticles led to a homing of the nanoparticles to brain microglial cells is significant since microglial cells play a pivotal role in CNS development and homeostasis.^[59,60]^ Chronic neuro-inflammation mediated by microglia is one of the pathological hallmarks of brain disease such as Alzheimer’s disease (AD).^[61,62]^

In this study, we also found that oatN entry into the brain via BBB is host HPCA dependent. HPCA is expressed in microglia cells and also in neuron cells. The preferential uptake of oatN by microglial rather than neuron cells could be due to the following reasons: 1) Unlike neuron cells which do not move around rapidly, trafficking via the brain vasculature, microglial cells are capable of rapidly migrating to where the tissue is damaged. The brain vasculature is different from the vasculature of other organs as it consists of the neurovascular unit (NVU), which is made up of various cell types consisting of vascular (i.e., endothelial cells, pericytes, smooth muscle cells, and perivascular macrophages (PVMs)) and brain (i.e., astrocytes and neurons) cells. A recent study showed that microglia is one of bona fide components of the NVU.^[63]^ 2) Unlike neuron cells, most data published indicate that the major function of microglia is to phagocytose particulate material including damaged and infected cells. Microglia express a wide array of receptors and thus rapidly respond to pleiotropic stimuli including edible plant exosomes-like nanoparticles. Our results show that once oatN enters the brain, it is subsequently taken up by microglia which is likely due to microglia having a stronger capacity for phagocytosis than neuron cells.

In this study, we also observed that the oatN signal in brain regions is different. Depending on the brain anatomical region, microglia account for 0.5–16.6% of the total cell population in the human brain^[64]^ and 5–12% in the mouse brain.^[65]^ In response to insult such as alcohol, microglia could also relocate to different regions of the brain and shift into different functional states, modifying the release of pro- and anti-inflammatory factors such as cytokines and chemokines that can be induced by alcohol. Therefore, uneven distribution of the oatN in different subregions of the brain could be due to an uneven location of microglia cells.

### 2.9. Edible Plant Nanoparticles Regulate Dectin-1 Trafficking

Our result show that oatN *β*-glucan cannot access to dectin-1 before the oatN enters microglial cells. Although we do not know molecular mechanism underlying how DGDG inhibits *β*-glucan access to dectin-1 on microglia membrane, it is clear that oatN DGDG and *β*-glucan are situated in two different microenvironments: oatN associated cell membrane versus microglial cell cytosol microenvironment. Before the entry into the microglia, the oatN in cell membrane associated microenvironment has an effect on the interaction of *β*-glucan with host cellular membrane HPCA, whereas after the entry into microglia, the interaction is under microglial cell cytosol control. It is conceivable that these two distinct environments will have different effect on the interactions. Here, using oatN as an example, we show that in the former case, *β*-glucan preferentially interacts with HPCA with DGDG participation, and in the latter case recruiting of host cell Rab11a to the HPCA/*β*-glucan complex occurs, subsequently leading to trafficking to Golgi where dectin-1 resides. Once in the Golgi, there is a “recycling pathway” that transports the HPCA/*β*-glucan/dectin-1 to recycling endosomes marked with Rab11 and then to the other intracellular vesicles including early endosome (EE) and lysosomes, concomitant with enhancing the movement of dectin-1 into exosomes. Therefore, increasing dectin-1 is not necessary to lead to activation of its downstream inflammatory pathway depending on the dectin-1intracellular location.

Our findings indicate that edible plant exosome like nanoparticles (ELN) regulate Rab11 mediated exocytic and recycling processes of dectin-1. Besides binding dectin-1 and HPCA, *β*-glucan has the ability to bind other molecules such as the *β*-integrin-type complement receptor CR3 and lactosylceramide.^[66,67]^ Therefore, our findings provide the foundation for further determining whether other molecules in oatN recipient cells are also recruited into the *β*-glucan/HPCA complex, what their role is in the oatN *β*-glucan mediated signaling pathway, and the immune response regulated by oatN *β*-glucan.

### 2.10. OatN *β*-Glucan Interaction with Hippocalcin (HPCA) Is Significant

Hippocalcin (HPCA) is a calcium-binding protein^[68,19,20]^ that is localized in the cytoplasmic and plasma membrane^[18]^ in neuron cells. HPCA is important for the homeostasis of intracellular calcium levels. HPCA KO mice have been shown to display memory deficits in spatial recognition tasks and impairment in the activation of Raf associated with the Ras signaling pathway.^[69–71]^ However, the HPCA role in microglial cells has not been studied. Our finding indicates that oatN *β*-glucan binding to HPCA is required for oatN entry into microglia cells and that oatN subsequently alters the Rab11a mediated exocytic and recycling processes of dectin-1. This finding provides a new avenue to further investigate whether *β*-glucan binding to HPCA has an effect on the levels of intracellular and extracellular calcium as a means to determine whether microglial cells crosstalk with neuron cells via an oatN mediated regulation of calcium levels, therefore maintaining homeostasis.

### 2.11. In the Context of OatN, *β*-Glucan, and DGDG Regulate Microglial Cell Activity

To date, most efforts to understand response pathways elicited by *β*-glucan have been conducted primarily in fungus therefore, very little is known about edible plant nanoparticles associated *β*-glucan-specific responses in microglia. Dectin-1 has been shown to mediate cellular recognition of *β*-glucan derived from a variety of fungal species. Upon *β*-glucan/dectin-1 receptor engagement, the phosphorylated receptor subsequently provides a docking site for Syk. Syk is required for *β*-glucan-induced production of ROS and cytokines/chemokines.^[72]^ Whether plant derived *β*-glucan in the context of oatN can activate the dectin-1 mediated inflammatory pathway is not known. In this study we show that oatN *β*-glucan binds to HPCA rather than dectin-1 via oatN DGDG mediated inhibition of *β*-glucan binding to dectin-1 that avoids activation of the alcohol mediated inflammatory pathway in microglial cells. We also found that oatN DGDG inhibits oatN *β*-glucan access to dectin-1 whereas there is no effect on *β*-glucan binding to HPCA before the oatN enters microglial cells.

DGDG is an abundant galactolipid present in thylakoid membranes and photosynthesis systems I and II, which is crucial for photosynthetic efficiency and plant development.^[73]^ DGDG is a bilayer-forming glycolipid which is responsible for the formation and stabilization of thylakoid membranes.^[74]^ DGDG is involved in stabilization of plant LHCII trimers and mediates the interactions between adjacent LHCII trimers.^[75,76]^ In cyanobacteria, DGDG may be involved in binding of extrinsic proteins to PSII and stabilizing the oxygen-evolving complex.^[77]^ The role of plant-derived DGDG in the context of oatN in regulating interaction of mammalian cell proteins such as dectin-1 and HPCA with plant-derived factors such as oatN *β*-glucan has not been previously studied. OatN consists of multiple molecules and the arrangement pattern of these molecules within and across the membrane of oatN could be affected by DGDG and contribute to inhibition of oatN *β*-glucan access to dectin-1. Detailed information of DGDG interacting factors that contribute to preferentially inhibit oatN *β*-glucan binding to dectin-1 but not HPCA may serve as a framework for future research as to the role of DGDG in oatN mediated inhibition of microglial cell activation. The mechanistic insights obtained will also improve our understanding on the roles on oatN associated DGDG and DGDG released from the oatN upon entry the microglial cells.

In addition, the current study raises several questions concerning the details of HPCA-dependent detin-1 endosomal trafficking. First, is there a HPCA domain(s) that binds to *β*-glucan, subsequently enhancing the recruitment of Rab11a? Second, are additional factor(s) recruited into the HPCA complex upon oatN stimulation leading to DGDG mediated inhibition of *β*-glucan binding to dectin-1? Third, HPCA is a calcium binding protein and calcium binding proteins have an effect on the retention of Rab11-positive vesicles. Whether changing intracellular Ca^[2+]^ levels influence recruiting Rab11a into the HPCA complex needs to be studied further. Lastly, another group has reported that dectin-1 is expressed in brain microglia but does not result in significant cytokine/chemokine production in microglia.^[78]^ The discrepancy between our results and theirs is that in the context of oatN *β*-glucans, tri-molecules, *β*-glucans, DGDG, and HPCA reside in the same compartment, and crosstalk to regulate microglial cell immune responses under an alcohol stress situation. Thus, our results indicate that the production of inflammatory cytokines seems to be dependent on the cooperation between a dectin-1-HPCA centered complex. *β*-glucan/dectin-1-mediated signaling in microglia represents a new pathway of interest in an anti-neuroinflammatory pathway mediated by natural products we consume daily. Moreover, although the dectin-1 mediated inflammatory pathway is known to contribute to brain inflammation, other NF-*κ*B mediated inflammatory pathway(s) induced by alcohol may also be inhibited by oatN cannot be excluded (Figure S8B, Supporting Information).

### 2.12. OatN Can Be Developed as a Functional Nano Food

A prime candidate to exploit for the production of nutraceutical foods is cereals. They are widespread crops throughout large parts of the world and an important part of the human diet. Cereal grains account for over 50% of the plant derived energy intake of humans and are the most consumed staple food worldwide (https://www.tandfonline.com/doi/pdf/10.1080/10942912.2018.1489837). The most abundant cereals in the human diet include wheat, barley, and oats. Cereals contain a variety of dietary fibers, of which *β*-glucans (BG) are among the most intensely studied.^[79,80]^ Consuming foods that contain at least 3 g oat BG per day (0.75 g per serving) provides beneficial effects including decreased serum lipid concentration, lowered blood pressure, attenuated blood glucose levels, reduced bowel transit time, and increased stool bulk.^[77,81^,^44]^ Despite the proven health-promoting properties of cereal BG, its potential as a nutraceutical ingredient is greatly underutilized. The reasons for this underutilization are numerous. The overall low concentration of BG in a cereal kernel is one of the major drawbacks. Thus, simple use of wholegrain products usually does not provide high enough levels of BG. The production of a BG concentrate is usually cost intensive, has low efficiency and induces structural changes, often resulting in the loss of the desired physiological effects.^[82]^ Based on the technology developed for purification of nanoparticles, large scale of production of oatN with column-based technology can overcome the forementioned difficulties and provide a successful production means for the nano food industry. The biological effects of oatN may not only be applicable for treating brain inflammation, but we also speculate it may have an effect on the macrophages located in other tissues as well, such as liver and intestine. BG has been reported to have the antitumor and antimetastatic activities, antimicrobial effects, inflammation-reducing effects, food allergy-alleviating effects, and stress-relieving effects.^[83–85]^ Therefore, our findings provide the basis for further investigating whether oatN also has the same or even better effects as BG alone.

## 3. Conclusion

Alcohol causes the chronic inflammation that contributes to neurodegenerative disease, yet no effective therapy has been developed for prevention of brain inflammation. This study focuses on the molecular mechanism(s) underlying how oatN contributes to the inhibition of microglial cell mediated inflammation. The findings from this study are significant in that it provides a basis for developing mechanism-driven novel approaches for prevention of microglial cell mediated chronic inflammation of the brain via non-invasive oral administration. The data generated from this study also suggest that future therapy may include ELNs as unique prebiotics to manipulate brain physiology in short- and long-term ways using specific ELNs by having a patients ingest ELNs to combat brain inflammation via the ELN-mediated enhancement of the brain immune homeostasis.

## 4. Experimental Section

### OatN Preparation:

Plant-derived exosome isolation and purification were performed according to our previous publications.^[28,86]^ In brief, oat bran meal was dissolved in PBS and was incubated in a 37 °C water bath for 30 min. The supernatant was collected and taken through serial centrifugations at 1000 × *g* for 10 min, 2000 × *g* for 20 min and 4000 × *g* for 30 min with the supernatant collected after each centrifugation. Oat microparticles (OatM) were pelleted at 10 000 × *g* for 60 min. The supernatant fractions of OatM juice were further spun down to isolate OatN by ultracentrifugation at 100 000 × *g* for 2 h at 4 °C. To purify the pellets, another centrifugation at 100 000 × *g* for 2 h on OptiPrep gradients (10%, 20%, and 40% OptiPrep in Tris-HCl buffer) was done. The visible bands of nanoparticles between the OptiPrep layers were collected and fixed in 2% paraformaldehyde. OatN were visualized using a Zeiss EM 900 electron microscope and characterization (size and concentration) was determined using the Nanosight (NS300).

### Extraction of *β*-Glucan from OatN:

OatN was dissolved in 0.1 m NaOH (pH = 10) and subjected to sonication for 30 min (400 W, 24 kHz). The *β*-glucan was extracted in boiling water for 2 h.^[87]^ The solution was acidified to remove proteins (pH = 3). After centrifuging the solution, the supernatant was collected, and *β*-glucan was precipitated in 60% ethanol overnight at 4 °C. Residual ethanol was removed by centrifuge and the pellet was dried by vacuum.^[88]^

### Liposomes Preparation:

Total lipid was isolated from oatN in accordance with our previous publication.^[28]^ Briefly, 1 mL oatN was thoroughly mixed with 3.75 mL MeOH and CHCl_3_ at a ratio of 2:1 v/v by vortex. Then 1.25 mL CHCl_3_ and ddH_2_O were sequentially added. The turbid sample was centrifuged at 2000 rpm for 10 min at room temperature generating two visibly separated phases. The bottom phase (organic phase) was aspirated using a Pasteur pipette without disturbing the upper aqueous phase. The collected samples dried by vacuum overnight. The dried lipid residue was hydrated in PBS (OLP) or *β*-glucan solution (OBG) for 30 min, after which liposomes were prepared by FS60 bath sonicator (Fisher Scientific, Pittsburg, PA) for another 30 min. Liposomes were pelleted by ultracentrifugation at 100 000 × *g* for 2 h and re-suspended in PBS. Characterization of liposomes based on size and concentration was accomplished using the Nanosight. To prepare HPCA coated oatN, an aliquot of oatN was supplemented with HPCA recombinant protein (50 mg mL^−1^) and incubated in 37 °C for 1–2 h with gently shaking. Unbound HPCA was removed by centrifugation for 45 min at 100 000 × *g* and the pellets were resuspended in PBS.

### β-Glucan Measurement:

The concentration of *β*-glucans in oatN was determined using a *β*-glucan assay kit (Megazyme) in accordance with provided instructions. OatN pellets (2 g) were accurately weight and mixed with 2.0 mL ice cold sulfuric acid (12 m). After incubating in an ice-water bath for 2 h, 4 mL of water were added to the mixture with vigorous vortexing. Tubes containing the mixture were placed in boiling water for 2 h and then transferred to a 100 mL volumetric flask. The mixture was neutralized using 10 m KOH solution and the flask was filled up with 200 mm sodium acetate buffer. One mL aliquots were digested at 40 °C for 60 min using *β*-glucanase (100 U mL^−1^). GOPOD reagent was added before transferring the volumetric flask to 40 °C water bath for 20 min. A glucose standard was used as a control. The absorbance of all solutions was measured at 510 nm on a plate reader (BioTek Synergy HT). Another group of oatN pellets were mixed with 2 mL of 2 m KOH and stirred in an ice bath for 20 min. Eight mL of 1.2 m CH_3_COONa buffer, 0.2 mL of amyloglucosidase and invertase (100 U mL^−1^) were added to the oatN pellets and incubated for 30 min in a 40 °C water bath with occasional vortexing. Absorbance of the solutions were determined using an OD Reader at a wavelength of 510 nm. The concentration of *β*-glucans was determined using the formula calculations below:

(1) Total glucan(%w/w)=ΔA×F/W×90

(2) a−Glucan(%w/w)=ΔA×F/W×9.27

(3) β−glucan=Total glucan−a−Glucan

Δ*A* = reaction absorbance – blank absorbance.

*F* = a factor to convert absorbance to mg of D-glucose.

### Polysaccharide Analysis:

Oat-derived polysaccharide was extracted by sonication for 20 min (400 W, 24 kHz) and then incubated for 2 h in a boiling water. After centrifuging at 5000 rpm for 10 min, the supernatants were collected and polysaccharide was precipitated overnight at 4 °C using a 75% ethanol solution. The solution was acidified to remove proteins (pH = 3). After centrifuging, the juice was collected, and resuspended in 75% ethanol overnight at 4 °C. Residual ethanol was removed by centrifugation and the pellet was dried under vacuum. High-performance liquid chromatography (HPLC) was performed on an Agilent 1260 system equipped with a TSKgel PW column (7.5 mm ID × 30 cm, G5000PW, TOSOH Bioscience) which separates different molecular weight polysaccharides. Deionized water was used as mobile phase and polysaccharide was detected by an evaporative light scattering detector (ELSD). Glycosyl composition analysis was carried out by combined gas chromatography-mass spectrometry (GC-MS) of the per-*O*-trimethylsilyl (TMS) derivatives of the monosaccharide methyl glycosides produced from both samples. In brief, 200 mg of the sample was heated for 18 h at 80 °C in methanolic. After cooling and removal of the solvent under a stream of nitrogen, the sample was treated with a mixture of methanol, pyridine, and acetic anhydride for 30 min at room temperature. The solvents were evaporated, and the sample was derivatized with Tri-Sil HTP Reagent (Thermo Scientific) at 80 °C for 30 min. After extraction with hexane, GC/MS analysis of TMS methyl glycosides was performed on an Agilent 7890A GC interfaced to a 5975C MSD, using a Supelco Equity-1 fused silica capillary column (30 m × 0.25 mm ID).

### Lipid Analysis:

Lipids from oatN were extracted and analyzed by Thin-Layer Chromatography (TLC) according to a method previously published.^[89]^ In brief, a glass plate was coated with silica gel 60, the coated plate was used to separate different types of polar or non-polar lipids. OatN-derived lipid samples were developed with chloroform/methanol/acetic acid (190:9:1 v/v/v). After drying in air, the TLC plate was placed in a chamber with a few iodine crystals and the lipid samples were visualized as yellow-brown bands on the TLC plate. Aliquoted lipid samples were analyzed at the Lipidomic Research Center, Kansas State University (Manhattan, KS) as previous described.^[28]^ In brief, the lipid samples were detected using a triple quadrupole MS (Biosystems Q-TRAP, Applied Biosystems, Foster City, CA). The results were displayed as the percentage of each lipid based on the total signal and compared to lipid database after normalization of the signals to internal standard controls.

### Animals:

C57BL/6 6-to 10-week-old female mice were purchased from Jackson Laboratory (Bar Harbor, ME). Mice were housed under specific-pathogen-free (SPF) conditions. Animal care was performed according to the institute for laboratory Animal Research (ILAR) regulations, and all animal procedures were approved by the University of Louisville Institutional Animal Care and Use Committee (Louisville, KY).

### OatN Labeling and Ex Vivo Brain Imaging:

Particle labeling with red fluorescent was performed according to the manufacturer’s instructions (Sigma, PKH26GL). In brief, 200 mg of oatN were resuspended in diluent C, and PKH26 ethanolic dye was added in an equal volume of diluent C. Particle suspension and dye solution were mixed and incubated for 5 min at room temperature (RT). OatN was pelleted by ultracentrifuge at 100 000 × *g* for 2 h to remove free dye. After washing twice in PBS, PKH26 labeled oatN was ready for use in experiments.

For ex vivo brain imaging, 200 mg of oatN were labeled with DiR dye (Thermo Fisher) at room temperature for 30 min, pelleted by ultracentrifugation at 100 000 × *g* for 2 h and the pellet washed in PBS. 200 mg of oatM were labeled with DiR dye (Thermo Fisher) at room temperature for 1 h, pelleted by centrifugation at 10 000 × *g* for 60 min and the pellet washed in PBS. Mice were gavage-given 0–800 mg labeled particles for the indicated time period and were then perfused using PBS. The brain and other organs were resected and imaged on an Odyssey scanner (excitation filter 750, and emission filter 800).

ExoGlow Vivo dye (SBI system Biosciences), a non-lipophilic dye, was used to covalently label oatN according to the manufacturer’s instructions. 200 mg of oatN were mixed with 2 mL ExoGlow Vivo dye and incubated at room temperature for 1 h. OatN from the labeling reaction was purified using ExoQuick-TC buffer provided in the kit. In brief, oatN was mixed with ExoQuick-TC buffer and incubated overnight at 4 °C. OatN was pelleted by centrifugation at 13 000 × *g* for 10 min to recover the nanoparticles and the pellet was resuspended in PBS. Mice were gavage-given labeled particles (8 mg kg^−1^ of body weight), rested for 6 h and were perfused using PBS. Brains were resected and imaged using Odyssey scanner (excitation filter 784, and emission filter 806).

### Clodronate Liposome Treatment:

Mice were anesthetized using 3% isoflurane inhalation and maintained by 1% isoflurane during surgery. Clophosome-A clodronate liposomes (Anionic, 5 mg mL^−1^, F70101C-A) were purchased from FormuMax (Sunnyvale, CA, USA). C57BL/6 mice were administrated 10 μL (1 mL stock diluted in 9 mL of PBS) of clodronate liposomes by injection into the brain parenchyma (0.8 mm anterior and 2.0 mm lateral of the bregma, 3.0 mm in depth) as previously reported.^[90]^ The needle was left in the brain for additional 3 min to allow for diffusion. To minimize pain and infection, buprenorphine and baytril were used after the intracranial injection. Body weights were monitored and compared before and after surgery.

### In Vitro Gastrointestinal Digestion:

The stability of the nanoparticles was determined using the in vitro digestion model which was previously published.^[32]^ On the first step, oatN were added to simulated gastric buffer (SGB, 3.2 g L^−1^ pepsin and 2.0 g L^−1^, pH = 1.2) at 37 °C for 1 h with gently rotation. On the second step, equal aliquots of the mixture were divided into two tubes. One of the tubes was mixed with simulated intestinal buffer (SIB, 2.0 g L^−1^ pancreatin, 8.8 g L^−1^ NaCl, 6.8 g L^−1^ monopotassium phosphate, and 12.0 g L^−1^ bile salts) after adjustment of pH from 1.2 to 7.5 to stop gastric digestion. The resulting sample was then incubated at 37 °C for another 2 h with gently rotation. The oatN from the two different treatments were spun down by ultracentrifugation at 100 000 × *g* for 1 h. Size and distribution of the pellet was detected using the Nanosight (NS300).

### In Vitro Endothelial Transwell Permeability Assay:

Endothelial cells (5 × 10^5^) were seeded on upper chamber (3.0 μm) of a HTS Transwell-24 well plate (Costar). After the cells reached 100% confluence, oatN labeled with PKH26 was added to the upper chamber for 3 h. The media from the bottom chamber at time points (0.5, 1, 2, and 3 h) were collected and the fluorescence intensity was determined using a Synergy Microplate Reader (BioTek) at 485/528 nm (emission and excitation wavelengths).

### Chronic Alcohol Consumption Model and Treatment:

C57BL/6 mice (6–10 weeks) were randomly divided into six groups and housed at 21 °C in a 12 h light/dark cycle (*n* = 5–8 per group). Mice were provided the rodent control liquid diet (BioServ, #F1259) or the 5% ethanol liquid diet (BioServ, #F1258). After acclimation for 5 days to the liquid diet, mice were gradually subjected to the 5% ethanol liquid diet for an additional 4 weeks. All mice were checked daily for food intake and health condition.

Mice were gavage-given either oatN (8 mg kg^−1^ of body weight), OLP (8 mg kg^−1^ of body weight), OBG (8 mg kg^−1^ of body weight), or HPCA-coated OatN (8 mg kg^−1^ of body weight) three times per week one mice were acclimated to the ethanol liquid diet. After 4 weeks, mice were subjected to the behavioral action testing or sacrificed.

### Cell Culture and Treatment:

The mouse microglial cell line (BV2) was provided by Dr. Behnam Badie (Beckman Research Institute of the City of Hope, Los Angeles, CA) and maintained in Dulbecco’s Modified Eagle Medium (DMEM, Gibco) supplemented with 10% fetal bovine serum (FBS, Gibco) in an incubator at 5% CO_2_, 37 °C. BV2 cells were treated for 24 h with ethanol (200 mm), ethanol plus oatN (20 mg mL^−1^), OLP (20 mg mL^−1^), OBG (20 mg mL^−1^), or HPCA-coated oatN (20 mg mL^−1^). Before treating neural primary cells with BV2 supernatant, BV2 cells were treated with ethanol (200 mm) or ethanol plus oatN (20 mg mL^−1^). Ethanol and particles were removed by PBS washing three times. Fresh medium was added to the BV2 cells and the supernatants were collected after 12 h (BV2 supernatants). Primary neural cells were co-incubated with BV2 supernatants for another 24 h.

### BV2 Cell Exosomes Isolation:

The supernatants from BV2 cell cultures were collected and processed through a series of centrifugations done at 1000 × *g* for 10 min, 2000 × *g* for 20 min, 4000 × *g* for 30 min, and 10 000 × *g* for 60 min. The supernatant after the 10 000 × *g* centrifugation was then ultracentrifuged at 100 000 × *g* for 2 h at 4 °C to isolate BV2 exosomes.

### Cellular Uptake Assay:

OatN (20 mg mL^−1^) were labeled with PKH26 and the labeled PKH26-oatN incubated with BV2 cells in 4 or 37 °C for 1 to 2 h. After the incubation, the cells were harvested and washed using PBS containing 2% FBS to remove free particles. Flow cytometry were used to analyze the ratio of PKH26 positive versus negative cells.

The cellular uptake mechanisms were determined by incubation of oatN with various inhibitors, including amiloride (Enzo, 1 mm), chlorpromazine (Sigma-Aldrich, USA, 20 μm), and filipin (Sigma-Aldrich, USA, 5 mg mL^−1^). BV2 cells were preincubated with one of the tested inhibitors for 1 h and then mixed with labeled oatN-PKH26 for 2 h. Free particles and inhibitor was removed followed by washing with PBS. Flow cytometry was used to analyze the ratio of PKH26 positive to negative cells.

### Antibody-Mediated Inhibition Assay:

BV2 cells were incubated antibody for 8 h at 37 °C with varying concentration of anti-HPCA. PKH26 labeled oatN was added to the cell medium for 2 h and then the percentages of nanoparticles taken up by BV2 cells were determined by FACS assay.

For in vivo assay, mice control IgG or anti-HPCA antibody (Thermo Fisher, 3 μg) was injected into opposite hemispheres of the brain at ±2 mm lateral, 0 mm anterior–posterior, and −1.5 mm deep relative to the intersection of the coronal and sagittal suture (bregma). The needle was left in the brain for additional 3 min to allow for diffusion. To minimize pain and infection, buprenorphine and baytril were used after the intracranial injection. After 48 h, mice were gavage given DiR dye labelled oatN (8 mg kg^−1^ of body weight) for 4 h and were then anaesthetized and perfused with PBS. Brain tissue was resected and imaged as previously described.

### Lentivirus Production and Infection:

Cas9 and sgRNA-containing plasmid (abm) and packaging plasmid (pCMV delta R8.2 and pCMV-VSV-G, addgene) were co-transfected into HEK293T cells. After 48 h and 72 h, supernatants were collected and centrifuged to remove cell debris. Lentivirus particles were concentrated by mixing with PEG8000 overnight and resuspended in DPBS buffer. BV2 were infected with lentivirus for 6 h and then puromycin selection (2 mg/mL) was performed 48 h post transduction. Cells were maintained for 7–14 days with antibiotics and a lentivirus producing monoclonal population was selected. HPCA or Rab11a knock out efficiency was determined by Western blot.

### RNA Extraction and RT-qPCR:

Total RNA was extracted from BV2 cells, neural cells, or murine brain tissue using the RNeasy mini kit (Qiagen) according to the manufacturer’s instructions. Briefly, cells or tissue were lysed in Buffer RLT. Samples were homogenized using a needle and syringe or tissue grinder. The supernatant was transferred to a fresh tube after centrifuging at 18 000 × *g* for 3 min. An equivalent volume of 70% ethanol was added to the tube, mixed well, and transferred to a RNeasy spin column. The flow-through was discarded after centrifugation, and the column was rinsed by Buffer RW1 and RPE sequentially. The total RNA was eluted by RNase-free water. The quality and quantity of the isolated RNA was measured using a NanoDrop spectrophotometer.

For analysis mRNA expression, 1 mg of total RNA was reverse transcribed by Superscript III reverse transcriptase (Invitrogen). The yield was quantified using SsoAdvancedTM Universal SYBR Green Supermix (BioRad). Q-PCR was performed on the BioRad CFX96 qPCR System and comparative threshold cycle (Ct) value was recorded. Relative fold changes of gene expression were calculated based on the following formula: 2^−ΔΔCt^. Primers used include the following: Rab11a-F: CAAGAAGCATCCAGGTTGATGGG and Rab11a-R: AAGGCACCTACTGCTCCACGAT, Rab11*β*-F: TGAGACCTCAGCCTTGGATTCC and Rab11*β*-R: CGGTCAGCGATTTGCTTCTGTG, Rab3*β*-F: CCTCCTTCCTTTTCCGCTATG and Rab3*β*-R: TCACACGCTTCTCATGGCG, Rab6a-F: GATACTGCGGGTCAGGAACG and Rab6a-R: GCAGCAGAGTCACGGATGTAA, Rab35-F: CCACAATCGGAGTGGATTTCA and Rab35-R: CGTCGTAAACCACAATGACCC, IL-1*β*-F: GTGTGCCGTCTTTCATTACACAG and IL-1*β*-R: CAGACCCTCACACTCAGATCATCT, TNF*α*-F: TCTATGGCCCAGACCCTCAC and TNF*α*-R: GACGGCAGAGAGGAGGTTGA, GAPDH-F: GGTCGGTGTGAACGGATTTG and GAPDH: GGAGTCATACTGGAACATGTAG.

### NF-κB Signaling Pathway PCR Array:

R&D System’s RT^2^ Profiler PCR Array Mouse NF-*κ*B Signaling Pathway (R&D System, PAMM-025ZE) was used to explore the effects of oatN on the NF-*κ*B signaling pathway gene expression in murine brain. Briefly, RNA isolated from mouse brain cerebrum was reverse transcribed to cDNA. PCR component mix (2x RT2 SYBR Green Mastermix, cDNA, and RNase-free water) was prepared and then loaded on the RT2 Profiler PCR Array. The array was tightly sealed with adhesive film and a PCR run (95 °C, 10 min for 1 cycle,95 °C, 15 s and 60 °C, 1 min for 40 cycles) was conducted. Ct values were calculated using the cycler software and data analysis was conducted using QIAGEN’S GeneGlobe Data Analysis Center.

### Murine Cytokine Array:

R&D System’s Proteome Profiler Mouse XL Cytokine Array Kit (R&D System, ARY028) was used to explore the effects of oatN on the regulation of cytokine expression in murine brain. In brief, excised brain cerebrum tissue was homogenized in PBS containing protease inhibitors (Complete, Millipore Sigma). After this step, 1% of Triton X-100 was added to the lysates and cell debris was removed by a 10 000 × *g* centrifugation for 5 min. After blocking and hybridization, the membrane array was exposed to X-ray film for 10 min. Cytokine levels were analyzed and quantified based on spot intensity. Spot intensity was quantified and normalized compared to reference spots. Fold changes in cytokines levels were compared to the spot intensity of mice treated with ethanol.

### Membrane Protein Isolation:

Membrane proteins were isolated base on the published literature^[91]^ BV2 cells or brain tissue were washed with HES buffer (20 mm Hepes, 1 mm EDTA, 250 mm sucrose, pH = 7.4) three times and then re-suspended in 1 mL HES buffer containing protease inhibitors. After disruption using a dounce homogenizer, the lysates were centrifuged at 196 000 × *g* for 1 h. The pellets were lysed using 0.5 mL of MBS (25 mm MES, 150 mm NaCl, pH = 6.5) with 0.5% Triton X-100 and further homogenized with the dounce homogenizer. The lysates were mixed with an equal volume of 80% w/v sucrose in MBS, and the mixture was carefully dispensed under 2.2 mL of 30% sucrose and 1.4 mL of 5% sucrose solution. Sample were centrifugated at 240 000 × *g* for 18 h and the rotor allowed to stop without braking. 400 mL of the top fraction containing highly enriched membrane protein weas collected from the gradients.

### Nanoparticle Pull-Down Assay and MS/MS Mass Spectrometry:

Murine brains were resected and washed in ice cold HES buffer containing protein inhibitors (cOmplete) and the fresh brain tissue was lysed using a dounce homogenizer. Membrane proteins were isolated as mentioned previously. The protein solution was pulled down by mixing with oatN overnight at 4 °C on a roller platform. The oatN were collected by ultracentrifugation at 120 000 × *g* for 1 h, the pellet rinsed twice in PBS, and resuspended by MS/MS lysis buffer (2% SDS, 100 mm DTT, 20 mm Tris-HCl pH 8.8). After 30 min with occasionally vortexing, the lysates were centrifuged at 120 000 × *g* for 1 h to remove particle debris. Supernatants were collected and analyzed by MS/MS tandem mass spectrometry (SRP121341). A mammalian database was used to identify the protein that was pulled down by oatN.

### Western Blotting and Immunoprecipitation Assay:

Cells or murine brain tissue were suspended in lysis buffer (20 mm Tris-HCl, 150 mm NaCl, 1 mm EDTA, 1 mm EGTA, and 1% NP-40) with proteinase inhibitor (cOmplete) and phosphatase inhibitor cocktail (Sigma). Samples were homogenized using a needle and syringe or tissue grinder. Debris were discarded after centrifugation at 13 000 × g for 10 min. Supernatants were collected and mixed with SDS loading buffer. After boiling for 5 min, 20–30 mg each protein sample were loaded on 8–15% SDS-Page gels, electrophoresed and then transferred to a PVDF membrane (BioRad Laboratories, Inc., Hercules, CA). The membrane was incubated with primary antibody (Anti-dectin-1, R&D system, AF1756, Anti-*p*-Syk, Cell Signaling, 2710S, Anti-*p*-NF-*κ*B p65, Cell Signaling, 3031S, Anti-*β*-Actin, Santa Cruz, sc-47778, Anti-HPCA, ThermoFisher, PA519535, Anti-Rab11A, Invitrogen, 71–5300, Anti-Rab11, Santa Cruz, sc-6565) over night at 4 °C and in Alexa Fluor-790 (Invitrogen, A11357 and A11369) conjugated secondary antibody for 1 h at room temperature. Bands were scanned and analyzed using an Odyssey imager (LiCor Inc, Lincoln, NE).

Cell or tissue lysates (1 mg) were incubated with 1 mg antibody at 4 °C overnight with gently rotation. Magnetic protein G or protein A beads (10 mL) were added to each sample to pull down the antibody complex. After washing in lysis buffer, the immunocomplex was eluted using either SDS loading buffer in boiling water for 5 min or glycine buffer (0.2 m, pH = 2.6) for 10 min with frequent agitation. Western blots were performed to further analyze the protein interaction.

### Confocal Image Assay:

Mice were euthanized with 2.5% v/v Avertin and had a transcardial perfusion done using ice-cold PBS. Fresh brain tissue was isolated and fixed in PLP buffer (0.075 m lysine, 0.37 m sodium phosphate, 2% formaldehyde and 0.01 m NaIO_4_). After being dehydrated using a 30% sucrose solution, the tissue was embedded in disposal mold containing Optimal Cutting Temperature (O.C.T) medium. The sample were frozen at −80 °C overnight. Next, the OCT mounted tissue was transferred to −20 °C freezer before it was cut into 8 μm thick sections using a cryotome. The sections were thawed and mounted onto charged microscope slides, dried on a 37 °C warming block and the tissue sections were blocked with a 5% BSA solution containing 0.3% Triton X-100. Diluted primary antibody (Anti-Iba-1, FujiFilm, 019–19741, Anti- *β*-Tubulin III, Sigma, T2200, Anti-dectin-1, R&D system, AF1756, Anti-Lamp-1, Santa Cruz, sc17768, Anti-EEA1, Cell Signaling, 3288S, Anti-HPCA, ThermoFisher, PA519535, Anti-Rab11A, Invitrogen, 71–5300, Anti-GM130, BD Transduction, 610822) was applied and incubated at 4 °C overnight. After rinsing twice in PBS, the specimens were stained with a fluorochrome-conjugated secondary antibody (Invitrogen, A32723 and A11005) for 1 h in the dark. DAPI staining were conducted before the slide was covered by anti-fade mounting buffer. The images were visualized using a Nikon A1R-A1 confocal microscope equipped with a digital image analysis system (Pixera, San Diego, CA).

Microglial cells were seeded and grown in 4 well chamber slides (Lab TekII, 154526). After treatment, the cells were washed with PBS and fixed by 4% paraformaldehyde for 20 min at room temperature. Next, the cells were blocked using 5% BSA solution containing 0.3% Triton X-100, stained with an indicated primary antibody and a fluorochrome-conjugated secondary antibody, and mounted using the identical protocol described above.

### Enzyme-Linked Immunosorbent Assay (ELISA):

The cytokines level of IL-1*β*, IL-6, IL-12, and TNF*α* in brain cerebrum tissue or cell culture medium was measured using the Set-Ready-Go ELISA kit (eBioscience). In brief, 96 microtiter plates were coated with capture antibody by incubating the plate overnight at 4 °C. Any remaining binding sites were blocked with ELISA diluent buffer for 1 h and then incubated with brain lysates or microglial cell supernatants for another 2 h. Before incubating with detection antibody, the plate was rinsed three times with PBS containing 0.05% Tween-20. Avidin conjugated with horseradish peroxidase was used to enhance the signal, substrate was subsequently added for 15 min. After stopping the reaction with 2n H_2_SO_4_, the plate was read at an absorbance of 450 nm.

### Isolation of Microglial Cells and Neuro from Brain:

Microglial cells were isolated from cerebrum of mice according to a previously described protocol.^[92]^ Murine brains were resected, mixed with digestion buffer ((HBSS containing 0.05% collagenase D (Worthington), 0.1% proteinase inhibitor cocktail (cOmplete), and 0.5% dispase (Gibco)) and minced using a dounce homogenizer. Cell suspensions were transferred to a fresh tube and rocked at room temperature for 15 min. After allowing for debris to settle for another 15 min, the cells were pelleted by centrifugation at 300 × *g* for 7 min. The pellets were resuspended in 37% stock isotonic Percoll (SIP). The sample mixtures were overlaid on the top of 4 mL of 70% SIP in a conical tube, followed by 4 mL 30% SIP and 2 mL HBSS. The gradients were centrifuged at 200 × *g* for 40 min without braking upon completion of the centrifugation to maintain the interphase of the gradient. The layer at the interface of the 37–70% SIP layers was removed and washed in HBSS. Cells were pelleted by centrifugation at 500 × *g* for 7 min.

Prior to isolate of primary neuro cells, 24-well plates were coated with 50 mg/mL poly-d-lysine (Gibco) overnight. An E17 timed pregnant mouse was sacrificed and the embryos were separated. Brains from the embryos were removed and the cortices were dissected. The tissue was centrifuged at 1000 rpm for 1 min and the pelleted tissue digested using 0.25% trypsin plus EDTA (Gibco) in a 37 °C incubator for 15 min with mixing every 5 min on a rocking platform. After adding DMEM with 5% FBS to neutralize the trypsin, the cells were pelleted by centrifugation at 1000 rpm for 5 min. Single cell suspensions were prepared by passing the resuspended pellets from the centrifugation step through a 40 mm filter. The single cell suspensions were counted and seeded into 24-well plates at a density of 2 × 10^5^ cells/well.

### Cell Viability:

Cell viability was assessed using the ATPlite Luminescence Assay System (PerkinElmer) according to manufacturer’s instructions. In brief, 100 mL of cell suspension were mixed on an orbital shaker at 700 rpm with 50 mL of mammalian cell lysis solution for 5 min, and then with 50 mL of substrate solution for another 5 min. The mixture was held in a dark room for 10 min before detecting the luminescence using a microplate reader (Synergy HT, BioTek, Winooski, VT).

### Flow Cytometry:

Isolated microglial cells from brain tissue and BV2 cells were washed in PBS twice. The cells were blocked with CD16/32 for 10 min and then stained for 30 min in a dark chamber with appropriate fluorochrome-conjugated antibodies (Anti-CD45-APC, eBioscience, Anti-CD11*β*-FITC, eBioscience, Anti-F4/80-Percp, eBioscience, Anti-Ly6G-Percp-PE, eBioscience). After washing thrice in PBS, flow cytometry was performed to analyze microglial cell populations using a BD FACSCalibur flow cytometer (BD Bioscience, San Jose, CA) and FlowJo software (Tree Star Inc., Ashland, OR).

### Surface Plasmon Resonance (SPR) Analysis:

Interaction and binding analyses of HPCA or dectin-1 protein were performed on OpenSPR instrument (Nicoya Lifesciences) and the procedures were carried out according to the manufacturer’s instructions. In brief, a sensor chip coated with nitrilotriacetic acid (NTA) was loaded into the instrument and the chip was charged with Ni^2+^ ions using a 40 mm NiCl_2_ solution. HPCA recombinant protein (MYBiosource) or dectin-1 recombinant protein (NOVUS) with His-tag were diluted in running buffer (HEPES containing NaCl (150 mm), 0.005–0.05% Tween 20, pH = 7.4) to a final concentration at 200 ng mL^−1^. HPCA or dectin-1 recombinant protein was immobilized on a chip after loading into the instrument. The amount of ligand binding was measured by comparing the signal before and after ligand injection.

### Histological Analysis:

Fresh brain tissues were resected and fixed in 10% formalin solution (SF93–20, Fisher Scientific, Fair Lawn, NJ) for 24 h at 4 °C. The specimens were then dehydrated by successive ethanol gradients of 70%, 80%, 95%, and 100% for 45 min each, after that, the tissues were immersed in xylene (Fisher) and embedded in paraffin. A microtome was used to cut ultrathin tissue slices (5 mm). Deparaffinization and rehydration were performed using xylene and ethanol gradients starting 100% followed by 95%, 80%, and 70%. Sections were stained with hematoxylin and eosin and, scanned using the Aperio ScanScope slide scanner.

### Novel Object Recognition (NOR) Test:

On the habituation phase, mice (*n* = 10 per group) were allowed free exploration of a 40 × 40 cm open-field box for 5 min. The arena was thoroughly cleaned between mouse use, using 70% v/v ethanol. On the first training run, two identical objects were placed in opposite quadrants of the box and the mice were allowed to familiarize themselves with the objects for 10 min. After 24 h, one of the objects was replaced with a novel object of different color and shape but in in the same location. Mice were placed in the open-field box and the time spend in discovery of the familiar and the new object was recorded during a 10 min test period.

### T-Maze Test:

Mice (*n* = 10 per group) were gently handled, habituated to the T-Maze apparatus, and subjected to food deprivation. On the forced alternation sample trail (T1), the animals were exposed to T-maze with one of the arms opened and rewarded a pellet of food at end of the arm. The opposite arm was blocked by a guillotine door. The mouse was allowed to explore the open arm and consume the reward. Ten consecutive trials were performed, and the mouse was removed. After 30 min, retrieval testing (T2) was performed. The guillotine door blocking the arm was removed, and mouse was placed in the same start position. If the mouse entered previously closed arm, its response was recorded “Correct,” and the animal received a reward. If the mouse entered the open arm from T1, the mouse was confined for 10 seconds and this response was marked as “Error.” Each mouse was subjected to ten consecutive runs. Forced alternation (%) was defined as the percent of mice first entering the novel arm during T2.

### Morris Water Maze:

Mice (*n* = 10 per group) were trained in a 122 cm diameter, 50 cm height open-field water maze with non-reflective interior surfaces. The pool was filled with water maintained at 25 ± 1 °C and mixed with non-toxic white tempera paint. Distinctive and extra-maze cues were marked on the wall at specific locations and were visible to the mice in the maze. Mice were removed from cages, placed into the water from quasirandom start points and allowed a maximum of 60 s to probe the escape platform. The inter interval of each trial remained for 15 s. Four trials were repeated per animal/per day for four subsequent days. Before beginning testing, the water in the tank was muddled by non-toxic white tempera paint. The mice were released to perform maze task and the time recorded when the mouse reached the escape platform.

### Electroencephalography (EEG) Recording in Mice:

Groups of mice were anesthetized by peritoneal administration of a ketamine (90 mg kg^−1^) and xylazine (10 mg kg^−1^) mixture. The mice were also injected with analgesic Buprenorphine (0.1 mg kg^−1^) to reduce pain. After removing the fur of skull area, the skin area was disinfected using betadine and 70% ethyl alcohol. A midline skin incision was made, and the skull was exposed for drilling. Two craniotomies (on frontal lobes and occipital cerebrum) on each side were made at the base on the skull midline (3 mm distance from midline vertical fissure in frontal region and occipital region) using a hand-held drill (Kopf). The electrodes were implanted over the frontal (FC) or occipital regions (OC) and the ground/reference electrode was implanted over the region of cerebellum. In this regard, two independent EEG channels were created for the FC and OC regions on each side of the hemisphere. Tissue adhesive (Vetbond) was used to fix the electrodes and denture adhesive (Fixodent ultra) was applied to cover exposed skull area to further keep the electrodes in place. Mice were left on a heating pad (37 °C) until waking. EEG recording was performed after post-operative recovery for 10 min using a Cascade PRO IONM system (Cadwell). EEG signals were amplified 1000× and digitized at 1 kHz. Data collection used a standard PC computer (Optiplex GX620, Dell) running Cascade IDNM software (Cadwell).

### Statistical Analysis:

To determine significant difference of the results, SPSS 16.0 software using one- or two-way ANOCA and Student’s *t*- test (*p < 0.05, **p < 0.01, ***p < 0.001, and ****p < 0.0001) was employed. A *p*-value greater than 0.05 was marked as not statistically significant (NS).

## Supplementary Material

Supplementary material

## Data Availability

The data that support the findings of this study are available from the corresponding author upon reasonable request.
