# Supplementary material for: Restoring Oat Nanoparticles Mediated Brain Memory Function of Mice Fed Alcohol by Sorting Inflammatory Dectin-1 Complex Into Microglial Exosomes
Source: Small. Author manuscript; Available in PMC 2023 Feb 1. (PMC8858573; doi:10.1002/smll.202105385)
Supplement: Supplementary material [file NIHMS1774068-supplement-Supplementary_material.pdf]

NANO MICRO
small

Supporting Information

for *Small,* DOI: 10.1002/smll.202105385

Restoring Oat Nanoparticles Mediated Brain Memory Function of Mice Fed Alcohol by Sorting Inflammatory Dectin-1 Complex Into Microglial Exosomes

Fangyi Xu, Jingyao Mu, Yun Teng, Xiangcheng Zhang, Kumaran Sundaram, Mukesh K. Sriwastva, Anil Kumar, Chao Lei, Lifeng Zhang, Qiaohong M. Liu, Jun Yan, Craig J. McClain, Michael L. Merchant, and Huang-Ge Zhang**

Supplementary figure legends

Figure S1 Characterization and composition of OatN

(A). OatN were isolated and purified by OptiPrep gradient solution (left panel). The oatN were collected between the 20%-40% OptiPrep layers and was quantified (n = 3) by weight (right panel).

(B). Size distribution of oatN was determined using a NanoSight NS300 (Westborough, MA) with a flow speed at 0.03 mL per min (left panel). A representative electron microscopic image of oatN (insert). Scale bar, 200 nm.

(C). After electrophoresis on a 1.2% polyacrylamide gel, oatN RNA was stained with ethidium bromide and visualized using an ultraviolet imaging system, RNA extracted from ginger exosome like nanoparticles (GNV) was used as a positive control.

(D). Proteins of oatN were separated by 10% sodium dodecyl sulfate polyacrylamide gel electrophoresis (SDS-PAGE) and stained with Coomassie blue.

(E). The lipid of oatN was isolated and developed using a TLC method.

(F). The lipid profile of oatN was determined by Tandem Mass (MS/MS) spectrometry.

(G, H). Molecular weight of oatN derived polysaccharide was detected using high-performance liquid chromatography (HPLC) equipped with a TSKgel PW5000 column (G). The joint peak was collected, and monosaccharide composition analysis was performed by on a GC-MS (H).

Figure S2 Oral administration of oatN trafficking to the brain are preferentially taken up by microglial cells

(A). Standard curve of fluorescence intensity from DiR free dye and DiR labeled oatM (Upper and bottom panel, respectively).

(B). Mice were treated with varying doses of DiR labeled oatN. After 4 h, mice were perfused and organs (lung, liver, spleen and kidney) and blood were scanned using an Odyssey imager (left panel). Fluorescent intensity was quantified (n = 3) (right panel).

(C). Size distribution of oatN (oatN1) after treatment of simulated gastro buffer (SGB, oatN2) and simulated intestinal buffer (SIB, oatN3) was determined using a NanoSight NS300. Biological activity of oatN after treatment of SGB and SIB was assessed by nanoparticles pull down assay with HPCA from mice brain lysates.

(D). Brain blood endothelial cells were planted on the upper of transwell chamber until reaching fully confluency. Cells were treated with PKH26 labeled oatN with or without ethanol (Eth) at 37 °C or 4 °C for 3 h. Fluorescent signals at different time points in bottom media were quantitatively analyzed by Microplate reader. *$P < 0.05$, **$P < 0.01$ by an ANOVA test.
(E). Mice were gavage-given ExoGlow^{TM}-Vivo labeled oatN (8 mg/kg of body weight). Animals were imaged using the Odyssey imager (Left panel) at 1, 4 and 6 h after gavage of ExoGlow^{TM}-Vivo labeled oatN. Brain tissue was excised and scanned after 6 h using the Odyssey imager (Right panel, top). ExoGlow^{TM}-Vivo labeled oatN was used as a positive control (Right panel, bottom). Bar graphs represent the mean of imaging intensity from 3 mice/group and SEM (n = 3 mice/group). ***$P < 0.001$ by ANOVA test.

Figure S3 OatN inhibits alcohol induced brain inflammation
(A). Mice (n=8-10) were fed a control liquid diet (CD) or CD containing 5% ethanol (Eth) while being gavage-given oatN (8 mg/kg of body weight), OLP (8 mg/kg of body weight), OBG (8 mg/kg of body weight) or HPCA coated OatN (8 mg/kg of body weight) three times per week for 28 days. Mice body weights were done every three days after mice were acclimated to Eth. Data represent the mean ± SEM.
(B). Liquid diet intake of mice (n=8-10) fed a control liquid diet (CD) or CD containing 5% ethanol (Eth) while being gavage-given oatN (8 mg/kg of body weight), OLP (8 mg/kg of body weight), OBG (8 mg/kg of body weight) or HPCA coated OatN (8 mg/kg of body weight) three times per week for 28 days. Mouse food intake were measured every three days after mice acclimated to Eth. Data represent the mean ± SEM.
(C). Mice (n=8-10) were fed a control liquid diet (CD) or CD containing 5% ethanol (Eth) while being gavage-given oatN (8 mg/kg of body weight), OLP (8 mg/kg of body weight), OBG (8 mg/kg of body weight) or HPCA coated OatN (8 mg/kg of body weight) every other day for 28 days. Blood ethanol concentration was measured using an alcohol reagent set (POINTE SCIENTIFIC, #A750439) and the value was expressed as the mean ± SEM.
(D). Mice were fed a control liquid diet (CD) or CD containing 5% ethanol (Eth) while being gavage-given oatN (8 mg/kg of body weight), OLP (8 mg/kg of body weight), OBG (8 mg/kg of body weight) or HPCA coated OatN (8 mg/kg of body weight) every other day for

28 days. Paraffin fixed brain sections were H&E stained; the arrows indicate inflammation in the hippocampus (CA2 region). Scale bar, 70 μm, Results represent 1 out of 3 independent experiments (n = 3).

(E). Mice (n=5) were fed a control liquid diet (CD) or CD containing 5% ethanol (Eth) while being gavage-given oatN (8 mg/kg of body weight), OLP (8 mg/kg of body weight), OBG (8 mg/kg of body weight) or HPCA coated OatN (8 mg/kg of body weight) every other day for 28 days. Inflammatory cytokine levels of IL-1β, IL-6 and TNFα in liver were detected using an ELISA. Bar graphs represent the mean of imaging intensity from 5 mice/group and SEM (n = 5 mice/group). ***, $P < 0.05$ by an ANOVA test.

Figure S4 OatN inhibits the protein expression of neuron cell TNFR1

(A). 4h after orally administration of PKH26-labeled oatN (8 mg/kg of body weight), mice brain cerebrum was fixed and embedded in O.C.T. Cryostat section were stained with anti-β-tublin (neuron cell marker, green); the confocal imaging assay was performed, and representative images were photographed. Results represent 1 out of 3 independent experiments (n = 3). Scale bars, 10 mm (top), 20 mm (bottom).

(B). Primary neuro cells, microglia and astrocytes were isolated from brain of mice embryos. HPCA expression level in these cells were determined by western blot (Top panel). PKH26 labeled OatN was respectively added to neuro and microglia cell medium. PE-positive cells at different time point (0, 1, 2 and 4 h) were quantitatively analyzed by FACS (Bottom panel).

(C). Primary neuro cells were incubated with the supernatants from 24h-cultured BV2 cells treated with Eth or Eth plus oatN for 24 h. FADD complex was pulled down using an anti-FADD antibody, TNFR1 was probed by western blot. Band intensity of TNFR1 or FADD were quantified and normalized by FADD or β-actin, respectively, and the results are presented between the panels.

Figure S5 HPCA is required for OatN up taken by microglial cells and inhibits the expression of inflammation cytokines released from microglial cell via interaction with Rab11a

(A). BV2 cells were transfected with CRISPR/Cas9 activation plasmid with Lipofectamine 3000. After drug selection for 14 days, the activation of HPCA ($HPCA^{ov}$) was confirmed by western blot (Left panel). Band intensity of HPCA was quantified and normalized to β-actin. and the results are presented between the panels. OatN was labeled with PKH26 and added to BV2 cell medium. PE-positive cells were quantitatively analyzed by FACS (second and third column from left panel). Bar graphs represent the mean of the FACS analysis results (n = 5). **P < 0.01, ***P < 0.001 by an ANOVA test.

(B). A representative western blot of HPCA in cerebrum lysates of mice fed a control liquid diet (CD) or CD containing 5% ethanol (Eth) while being gavage-given oatN (8 mg/kg of body weight). Band intensity was quantified and normalized to β-actin; bar graphs represent the mean of the intensity analysis results and SEM (n = 3).

(C). A representative western blot of HPCA in BV2 cells treated Eth (200 mM) or Eth plus oatN (20 mg/mL) was shown. Band intensity was quantified and normalized to β-actin; bar graphs represent the mean of the intensity analysis results and SEM (n = 3).

(D). A representative western blot of Rab11a and in BV2 cell lysates treated Eth (200 mM) or Eth plus oatN (20 mg/mL). Band intensity was quantified and normalized to β-actin; bar graphs represent the mean of the intensity analysis results and SEM (n = 3). **P < 0.01 by an ANOVA test.

(E). BV2 cells were infected with CRISPR/Cas9 Rab11a KO lentivirus. A representative western blot of Rab11a is shown. Band intensity was quantified and normalized to β-actin; bar graphs represent the mean of the intensity analysis results and SEM (n = 5). ***P < 0.001 by the Student t test.

Figure S6 OatN DGDG prevents dectin-1 mediated activation of microglial cells

(A). Varying concentration from 10 to 90 (mg/mL) of BG was packed into liposomes made from the total lipids extracted from oatN (OBG). OBG was labeled with PKH26 and added to BV2 cell medium. PE-positive cells were quantitatively analyzed by FACS (left panel). Bar graphs represent the mean of the flow cytometry analysis results (n = 5). **P < 0.01 by an ANOVA test.

(B). BV2 cells were pretreated with amiloride (1 mM), chlorpromazine (20 mM) or filipin (5 mg/mL) for 1 h, and then continued in culture in the present of PKH26 labeled oatN for 2 h.

PE-positive cells were quantitatively analyzed by FACS (upper panel). Bar graphs (bottom panel) represent the mean of the flow cytometry analysis results (n = 5). ***$P < 0.001$ by an ANOVA test.

(C, D). Mice (n=5) were administrated a control liquid diet (CD) or CD containing 5% ethanol (Eth) in the present or absent of laminarin (LAM, 300 mg/kg of body weight). TNFα level were determined by ELISA (C) and phosphorylation of Syk and p65-NF-κB in brain tissue lysates were determined using western blots (D), respectively. Band intensity is normalized by β-actin and are presented between the panels. Bar graphs (C) represent the mean of the ELISA analysis results (n = 5) and SEM. **$P < 0.01$ by an ANOVA test.

(E, F). BV2 cells were treated with Eth (200 mM) in the present or absent of LAM (300 mg/mL). TNFα level was determined using an ELISA (E), and phosphorylation of Syk and p65-NF-κB in brain tissue lysates were determined using western blots, respectively (F). Bar graphs (E) represent the mean of ELISA analysis results and SEM (n = 5). **$P < 0.01$ by an ANOVA test. Band intensity (F) is normalized to β-actin and are presented between the panels.

(G, H). BV2 cells were treated with Eth (200 mM) or Eth plus OBG (20 mg/mL) or the free form of BG (BG, 20 mg/mL). TNFα level in cells medium were determined by using an ELISA. Bar graphs (G) represent the mean of the ELISA analysis results (n = 3) and SEM. *$P < 0.05$, **$P < 0.01$, NS, not statistically significant by an ANOVA test. Band intensity (H) are normalized to β-actin and the result are presented between the panels.

(I, J). BV2 cells were treated with Eth (200 mM) or Eth plus OBG (20 mg/mL), OBG with depleted PC (OBG-PC, 20 mg/mL) or OBG with depleted DGDG (OBG-DGDG, 20 mg/mL). (I). TNFα level in cell medium were determined using an ELISA assay. Bar graphs (I) represent the mean of ELISA analysis results (n = 5) and SEM. *$P < 0.05$, NS, not statistically significant. by an ANOVA test. (J). Phosphorylation of SyK and p65-NF-κB were evaluated by Western blots. Band intensity (H) is normalized to β-actin and the result are presented between the panels.

(K). BV2 cells were incubated with PKH26 labeled oatN (red, 20 mg/mL) for 1 h. Cells were stained with dectin-1 or HPCA (green) for confocal analysis. Scale bars, 20 mm.

(L). BV2 cells pretreated with/without β-glucanase (100 U/mL) digestion were incubated with PKH26 labeled oatN (red) in the present of amiloride (1 mM) for 1 h. Cells were stained

with HPCA (green) for confocal analysis. Five random fields were photographed and representative results are shown. Scale bars, 20 mm.

(M). HPCA, Rab11a or Dectin-1 complex in BV2 cells treated with oatN (20 mg/mL) was respectively pulled down with an anti-HPCA, anti-Rab11a or Dectin-1 antibody (1 mg/mL) and then β-glucan content was detected. Bar graphs represent the mean of β-glucan content (n = 3) and SEM.

(N). HPCA complex in BV2 cells treated with oatN (20 mg/mL) was pulled down with an anti-HPCA antibody (1 mg/mL) and then used to perform TLC. OatN and DGDG standards (50 mg/mL) were loaded as controls. Results represent 1 out of 3 independent experiments

Figure S7 RAb11a alters the trafficking routes of dectin-1 of microglial cells treated with oatN and inhibits phosphorylated Syk

(A). BV2 cells were treated with Eth (200 mM) plus OBG (20 mg/mL) at the indicated time points. Fluorescent images of microglial cells were stained with dectin-1 (green) overlaid with EEA1 (red) or LAMP-1 (red) and DAPI (blue). Results represent 1 out of 3 independent experiments. Five random fields were photographed and representative results are shown. Scale bars, 20 mm.

(B). A representative western blot of *p*-Syk detected in BV2 wild type cells and Rab11a knockout cells treated with Eth (200 mM) or Eth plus OBG (20 mg/mL). Band intensity are normalized to β-actin and the results are presented between the panels.

(C). A representative western blot of *p*-Syk in BV2 wild type cells and HPCA knockout cells treated with Eth (200 mM) or Eth plus OBG (20 mg/mL). Band intensities are normalized to β-actin and the results are presented between the panels.

(D). Size distribution of nanoparticles derived from BV2 cells was determined using a NanoSight NS300 (Westborough, MA) with a flow speed at 0.03 mL per min (left panel). A representative electron microscopic image of BV2 derived exosomes (Right panel). Scale bar, 200 nm (right panel).

(E). Exosomes isolated from 24 h cultured BV2 treated with OBG were stained with goat anti-dectin-1 primary antibody and followed by rabbit anti-Goat 594 second antibody. Nanolight3000 was used to quantify the total number of exosomes and PE-dectin-1 positive exosomes. Results represent 1 out of 3 independent experiments.

Figure S8. OatN alters the trafficking routes of dectin-1 expressed in brain of mice fed an alcohol liquid diet

(A). Mice (n=5) were fed a control liquid diet (CD) or CD containing 5% ethanol (Eth) while being gavage-given oatN (8 mg/kg of body weight) or OBG (8 mg/kg of body weight), respectively, every other day for 28 days. Brain frozen sections were co-stained with dectin-1 and Rab11a, EEA1 or LAMP (red). Five random fields are photographed, and representative results were shown. Scale bars, 20 mm.

(B). Mice (n=5) were fed a control liquid diet (CD) or CD containing 5% ethanol (Eth) while being gavage-given oatN (8 mg/kg of body weight) every other day for 28 days. RNA extracted from murine cerebrum was used in a NF-κB signaling pathway PCR array. A bar graph showing the relative changes of NF-κB regulated genes.

Fig. S1

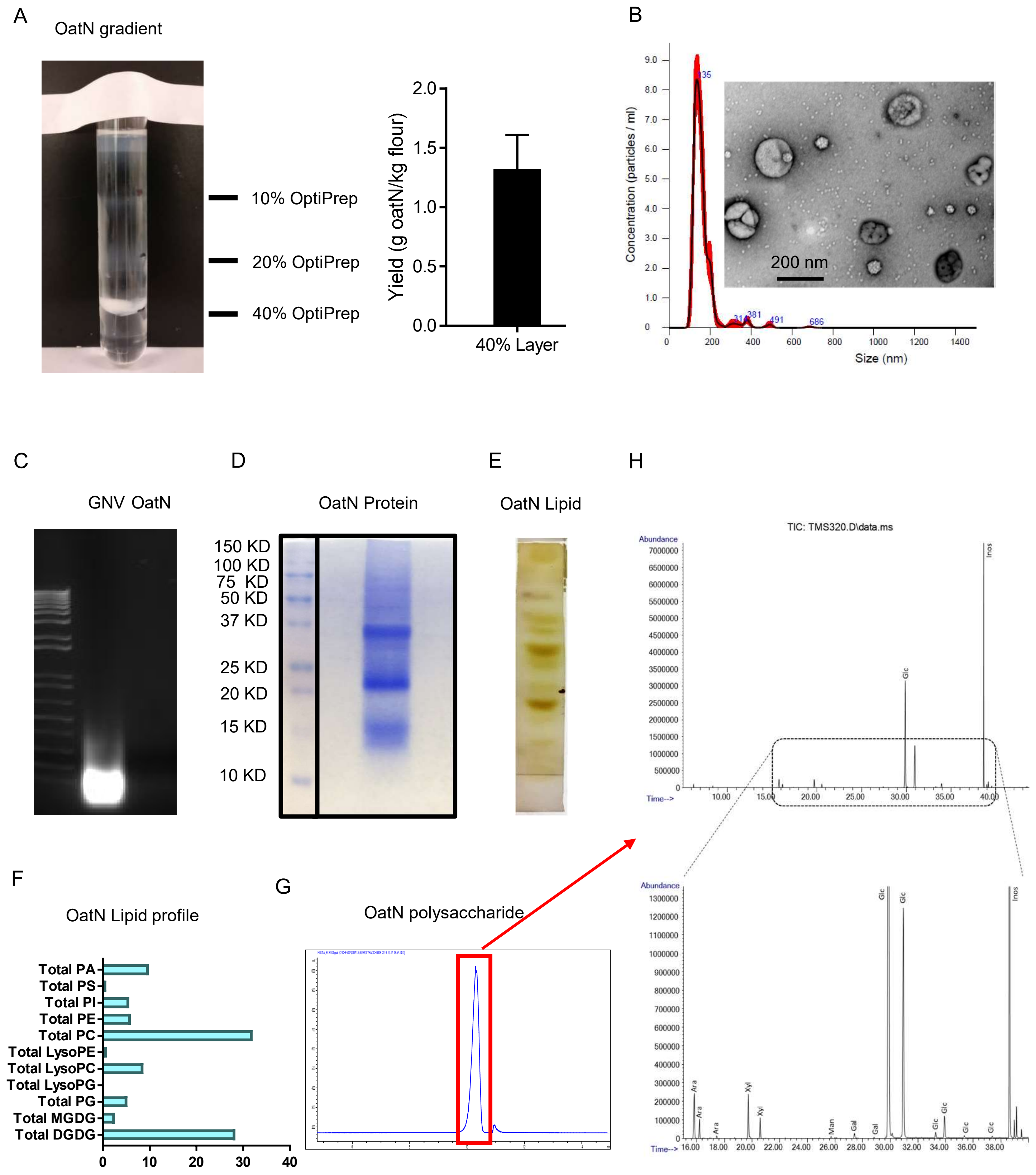
A
OatN gradient
10% OptiPrep
20% OptiPrep
40% OptiPrep
Yield (g oatN/kg flour)
2.0
1.5
1.0
0.5
0.0
40% Layer
B
Concentration (particles / ml)
Size (nm)
200 nm
135
314
381
491
686
C
GNV OatN
D
OatN Protein
150 KD
100 KD
75 KD
50 KD
37 KD
25 KD
20 KD
15 KD
10 KD
E
OatN Lipid
H
TIC: TMS320.D\data.ms
Abundance
Time-->
Glc
Inos
Ara
Xyl
Man
Gal
F
OatN Lipid profile
Total PA
Total PS
Total PI
Total PE
Total PC
Total LysoPE
Total LysoPC
Total LysoPG
Total PG
Total MGDG
Total DGDG
0
10
20
30
40
G
OatN polysaccharide

Fig. S2

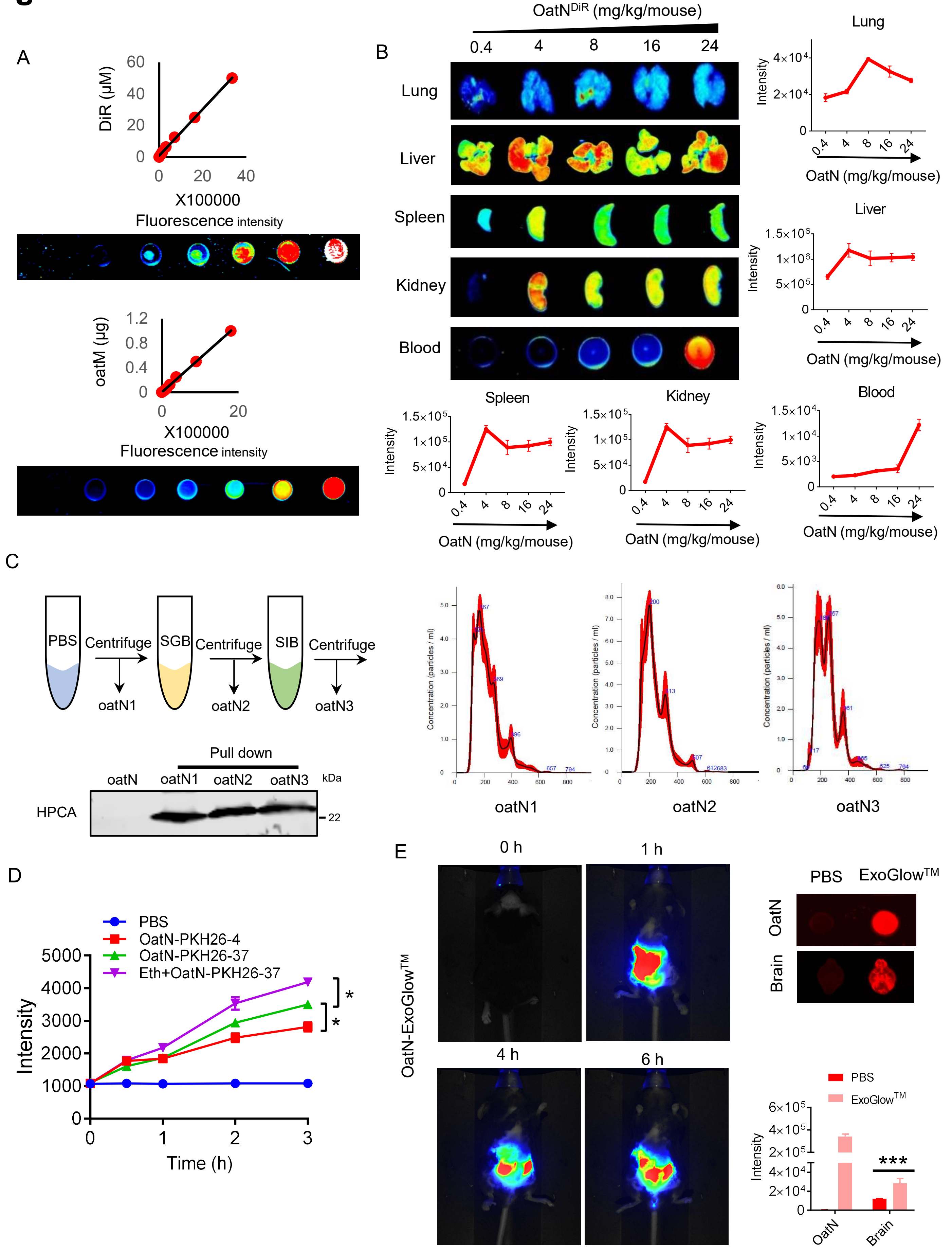

A
DiR (μM)
X100000
Fluorescence intensity
oatM (μg)
X100000
Fluorescence intensity
B
OatN^DiR (mg/kg/mouse)
0.4 4 8 16 24
Lung
Liver
Spleen
Kidney
Blood
Intensity
OatN (mg/kg/mouse)
C
PBS
Centrifuge
SGB
SIB
oatN1
oatN2
oatN3
Pull down
oatN oatN1 oatN2 oatN3 kDa
HPCA
22
Concentration (particles / ml)
D
PBS
OatN-PKH26-4
OatN-PKH26-37
Eth+OatN-PKH26-37
Intensity
Time (h)
E
0 h
1 h
4 h
6 h
OatN-ExoGlow™
PBS ExoGlow™
OatN
Brain
***

Fig. S3

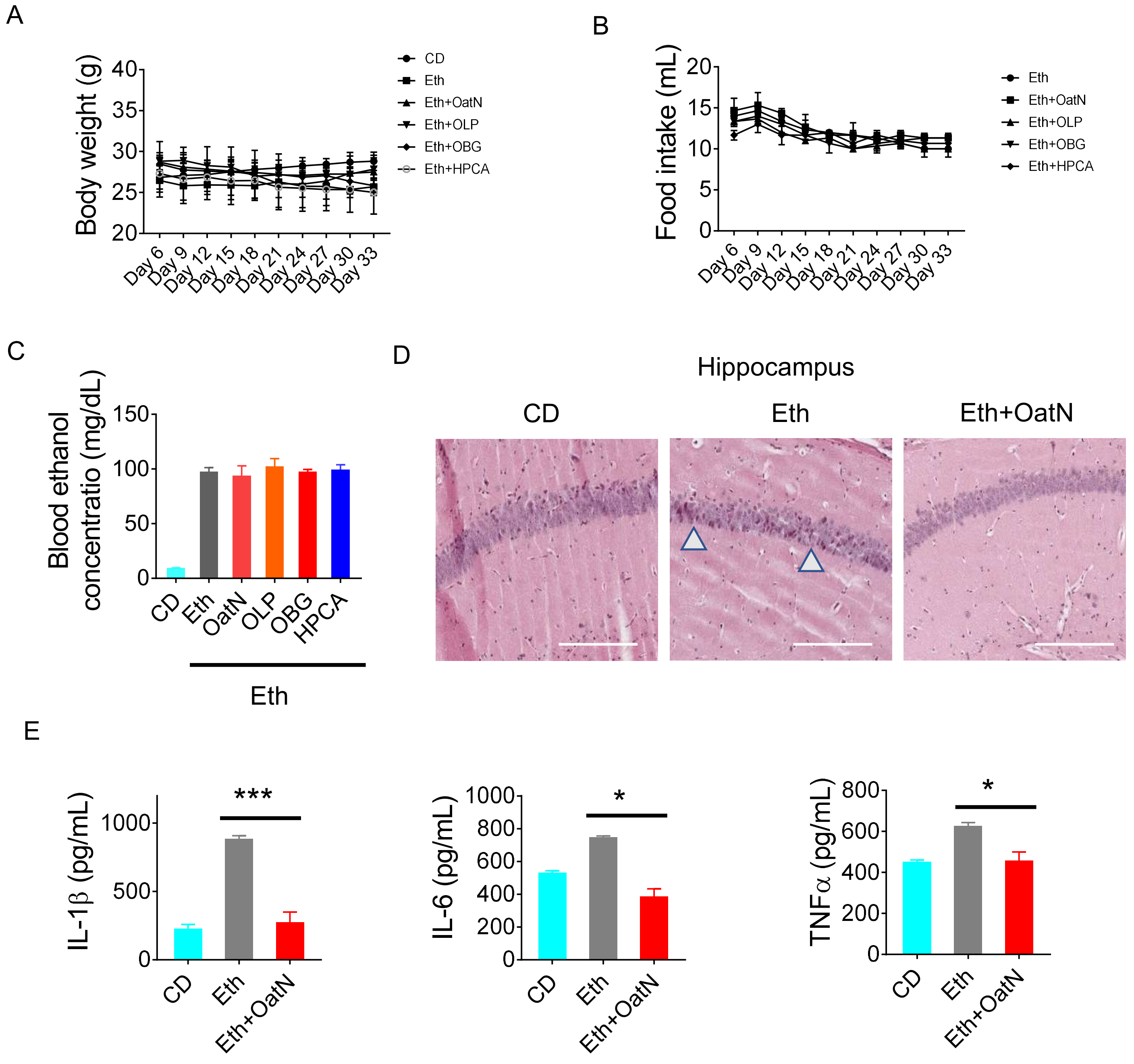

A
Body weight (g)
CD
Eth
Eth+OatN
Eth+OLP
Eth+OBG
Eth+HPCA
B
Food intake (mL)
Eth
Eth+OatN
Eth+OLP
Eth+OBG
Eth+HPCA
C
Blood ethanol concentratio (mg/dL)
CD
Eth
OatN
OLP
OBG
HPCA
Eth
D
Hippocampus
CD
Eth
Eth+OatN
E
IL-1β (pg/mL)
IL-6 (pg/mL)
TNFα (pg/mL)
CD
Eth
Eth+OatN
***
*
*

Fig. S4

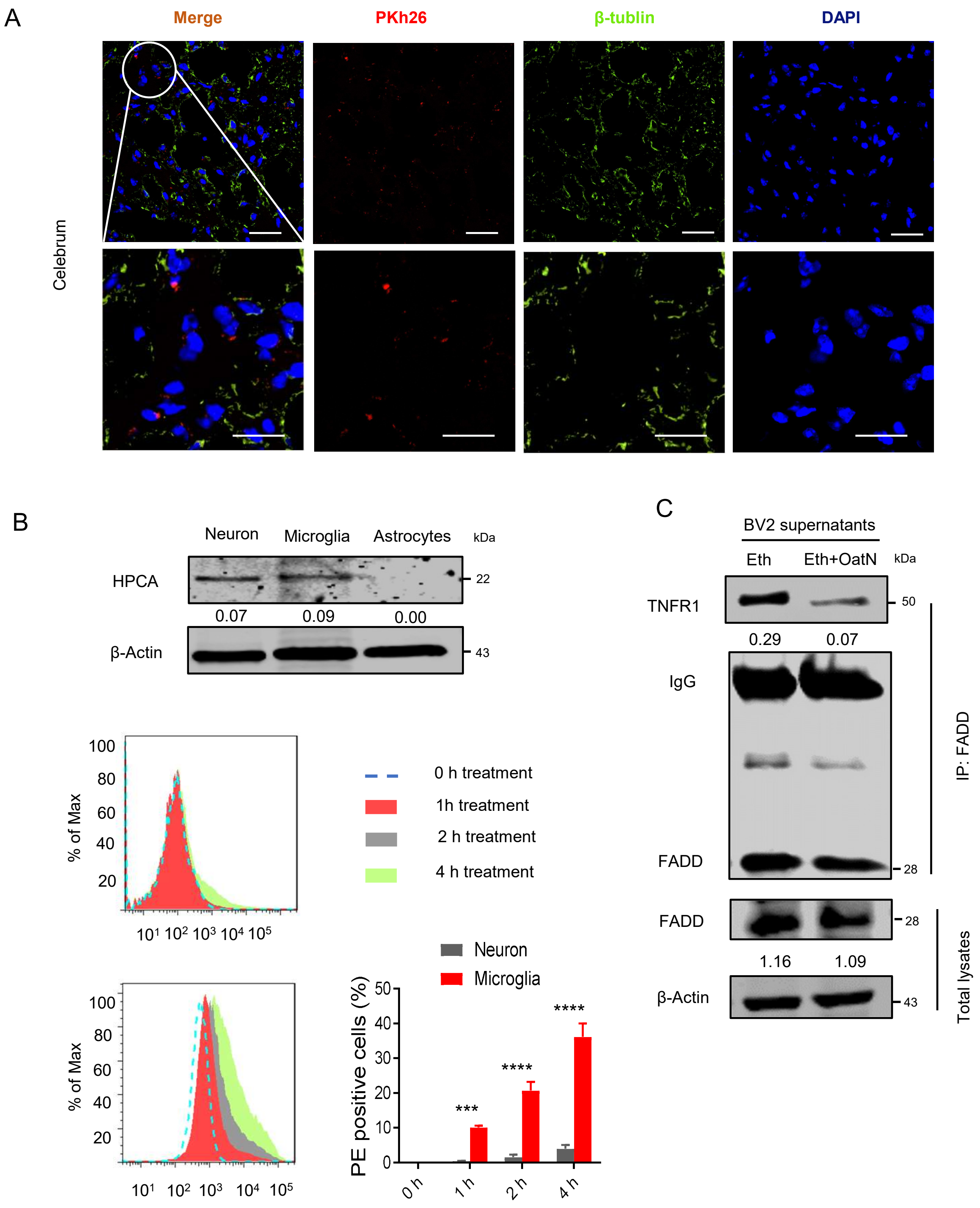

A
Merge
PKh26
β-tublin
DAPI
Celebrum
B
Neuron
Microglia
Astrocytes
kDa
HPCA
22
0.07
0.09
0.00
β-Actin
43
% of Max
100
80
60
40
20
10¹ 10² 10³ 10⁴ 10⁵
0 h treatment
1h treatment
2 h treatment
4 h treatment
Neuron
Microglia
PE positive cells (%)
50
40
30
20
10
0
***
****
****
0 h
1 h
2 h
4 h
C
BV2 supernatants
Eth
Eth+OatN
kDa
TNFR1
50
0.29
0.07
IgG
FADD
28
IP: FADD
FADD
28
1.16
1.09
β-Actin
43
Total lysates

Fig. S5

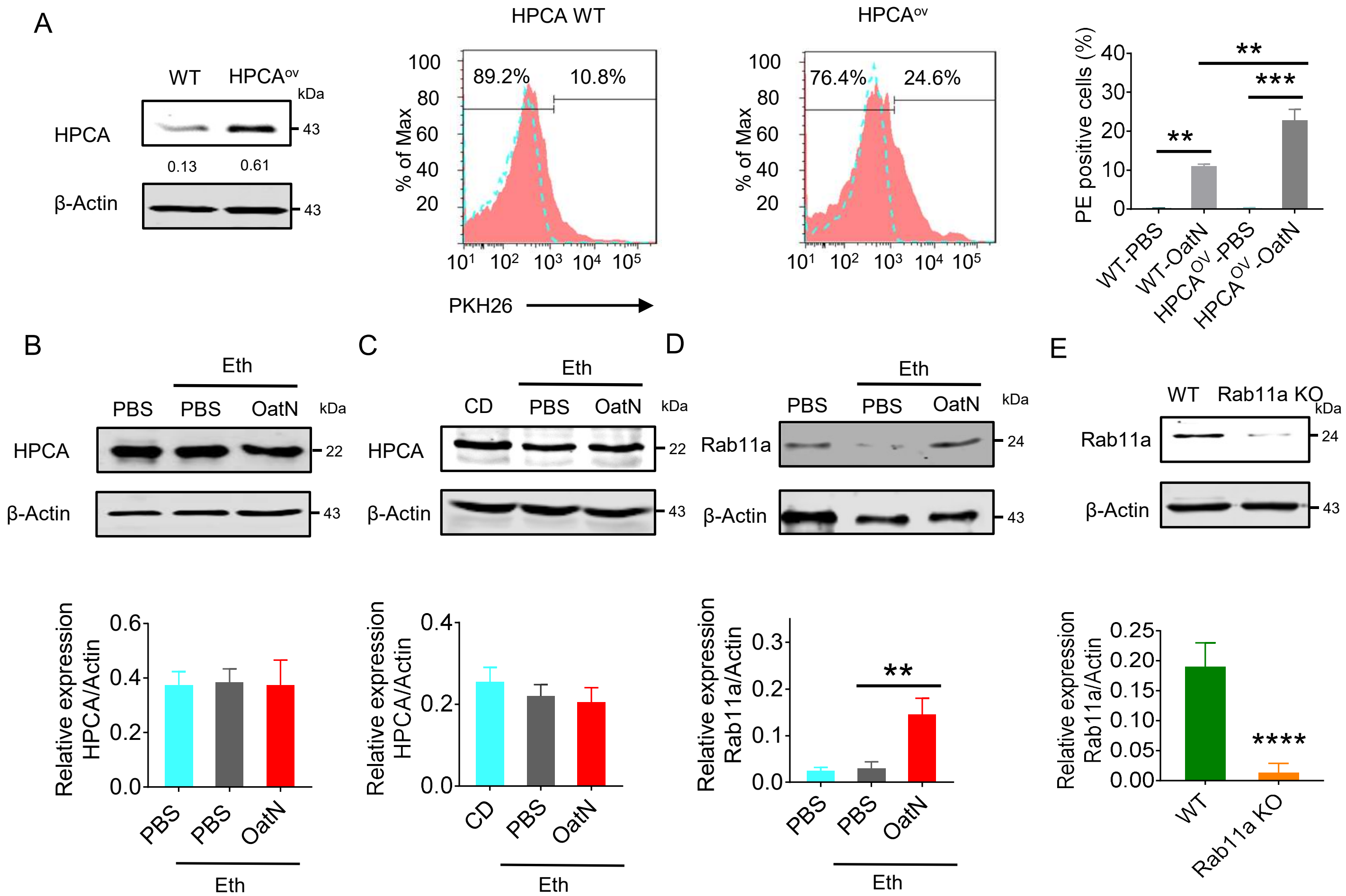

A
WT
HPCAov
kDa
HPCA
43
0.13
0.61
β-Actin
43
HPCA WT
HPCAov
100
80
60
40
20
% of Max
89.2%
10.8%
76.4%
24.6%
PKH26
PE positive cells (%)
WT-PBS
WT-OatN
HPCAOV -PBS
HPCAOV -OatN
**
***
B
Eth
PBS
PBS
OatN
kDa
HPCA
22
β-Actin
43
Relative expression HPCA/Actin
C
CD
PBS
OatN
D
Rab11a
24
Relative expression Rab11a/Actin
E
WT
Rab11a KO
****

Fig. S6

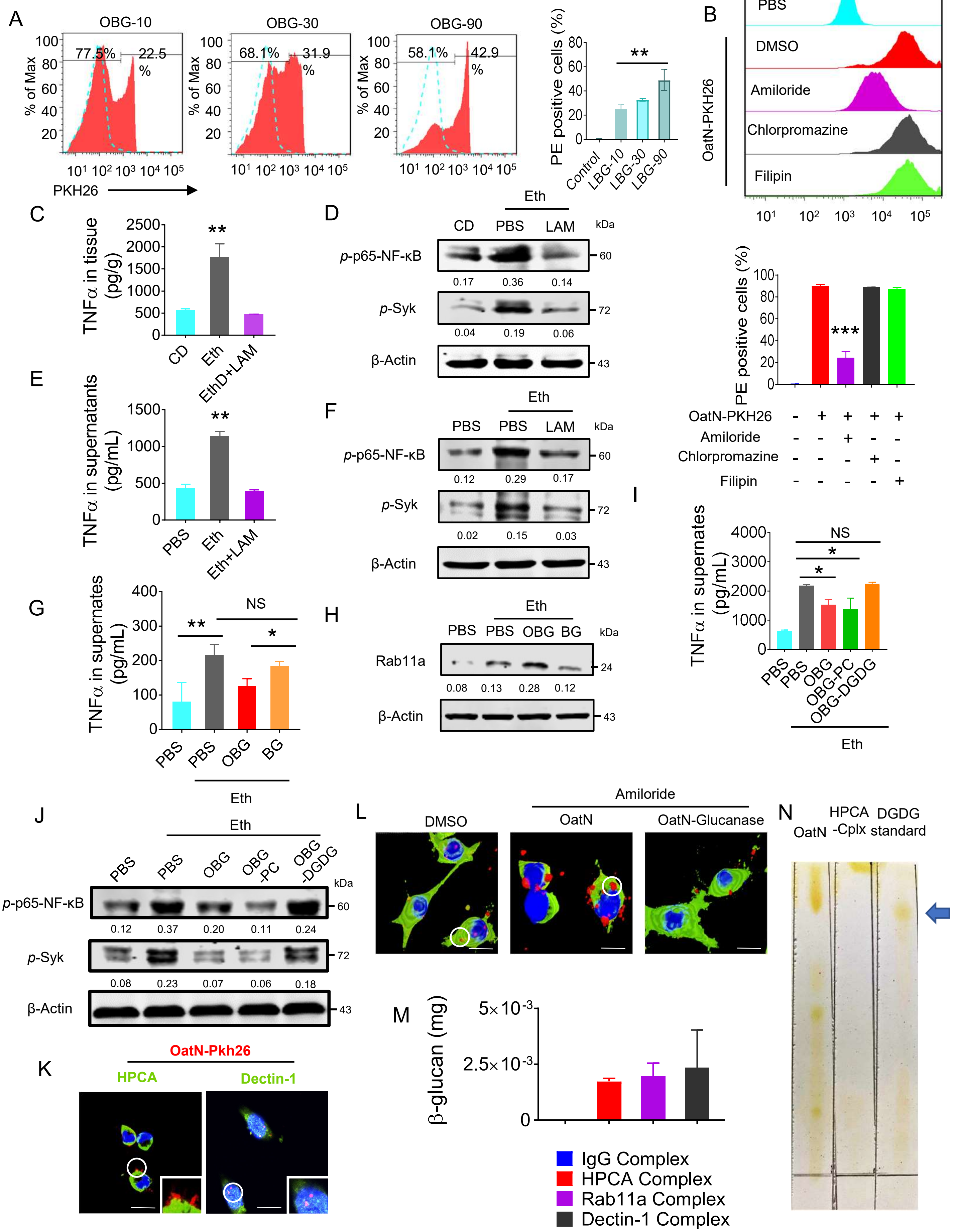
A
OBG-10
OBG-30
OBG-90
77.5%
22.5 %
68.1%
31.9 %
58.1%
42.9 %
% of Max
PKH26
PE positive cells (%)
**
Control
LBG-10
LBG-30
LBG-90
B
PBS
DMSO
Amiloride
Chlorpromazine
Filipin
OatN-PKH26
C
TNFα in tissue (pg/g)
CD
Eth
EthD+LAM
D
Eth
CD
PBS
LAM
kDa
p-p65-NF-κB
p-Syk
β-Actin
0.17
0.36
0.14
0.04
0.19
0.06
E
TNFα in supernatants (pg/mL)
PBS
Eth
Eth+LAM
F
0.12
0.29
0.17
0.02
0.15
0.03
***
OatN-PKH26
Amiloride
Chlorpromazine
Filipin
G
TNFα in supernates (pg/mL)
NS
OBG
BG
H
Rab11a
0.08
0.13
0.28
0.12
I
OBG-PC
OBG-DGDG
J
0.12
0.37
0.20
0.11
0.24
0.08
0.23
0.07
0.06
0.18
K
OatN-Pkh26
HPCA
Dectin-1
L
Amiloride
DMSO
OatN
OatN-Glucanase
M
β-glucan (mg)
IgG Complex
HPCA Complex
Rab11a Complex
Dectin-1 Complex
N
HPCA -Cplx
DGDG standard

Fig. S7

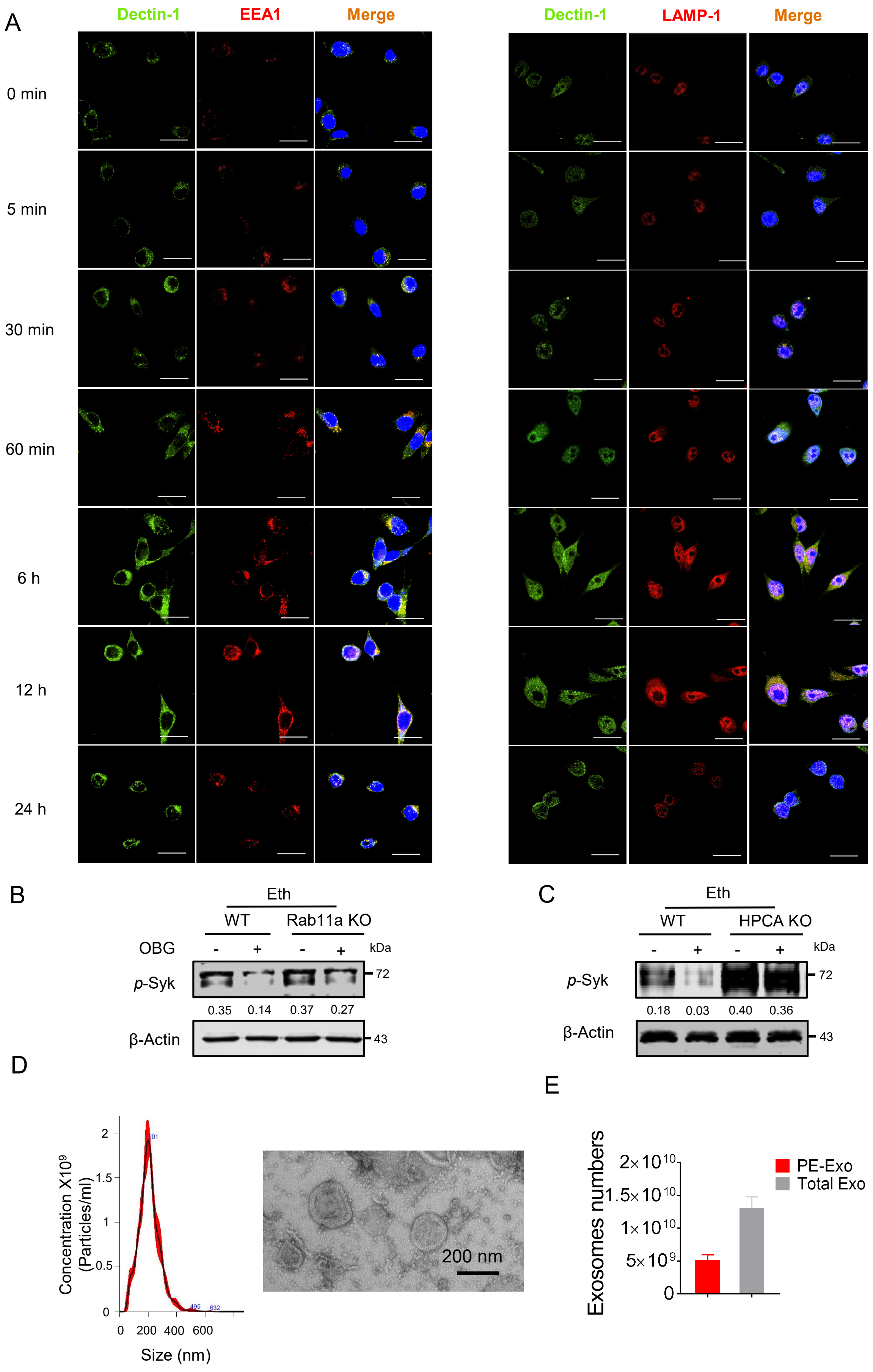
A
Dectin-1
EEA1
Merge
Dectin-1
LAMP-1
Merge
0 min
5 min
30 min
60 min
6 h
12 h
24 h
B
Eth
WT
Rab11a KO
OBG
- + - +
kDa
p-Syk
72
0.35 0.14 0.37 0.27
β-Actin
43
C
Eth
WT
HPCA KO
- + - +
kDa
p-Syk
72
0.18 0.03 0.40 0.36
β-Actin
43
D
Concentration X10^9 (Particles/ml)
Size (nm)
0 200 400 600
0 0.5 1 1.5 2
200 nm
E
Exosomes numbers
0 5×10^9 1×10^10 1.5×10^10 2×10^10
PE-Exo
Total Exo

Fig. S8

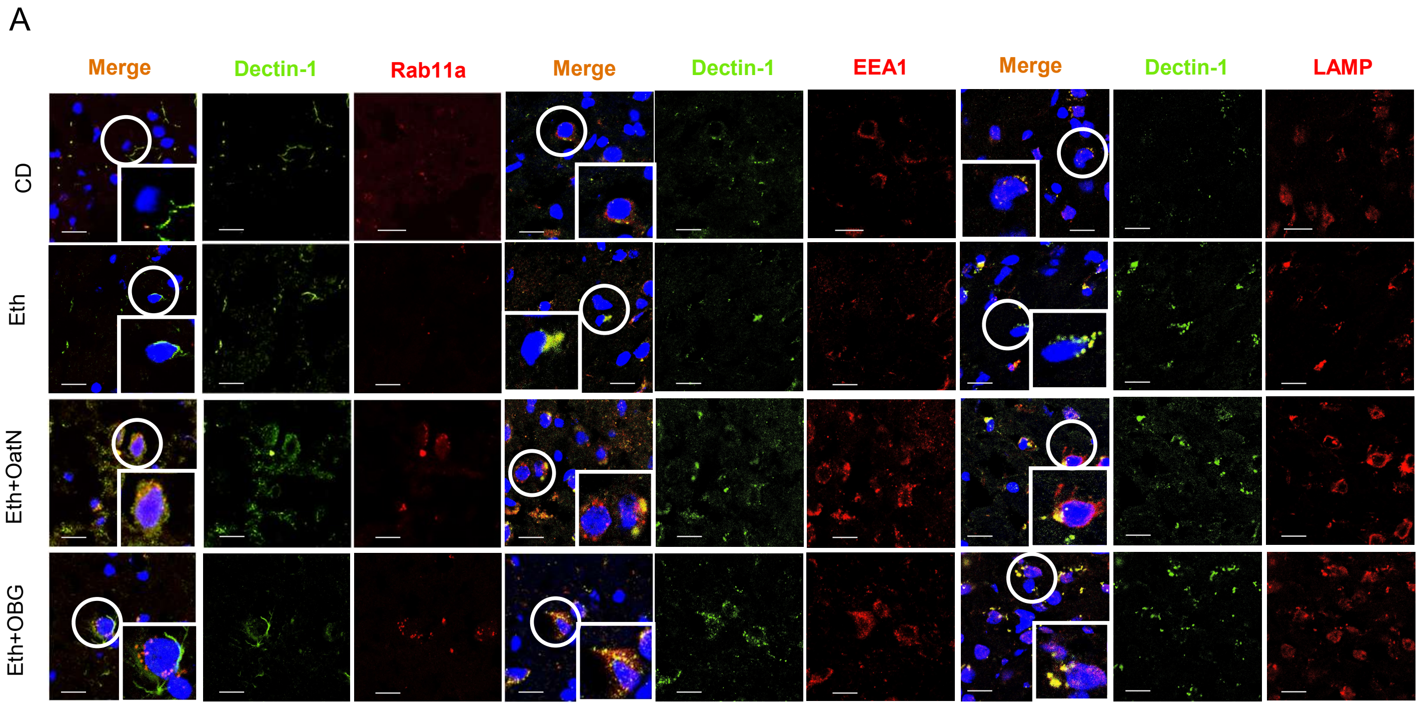
A
Merge
Dectin-1
Rab11a
Merge
Dectin-1
EEA1
Merge
Dectin-1
LAMP
CD
Eth
Eth+OatN
Eth+OBG

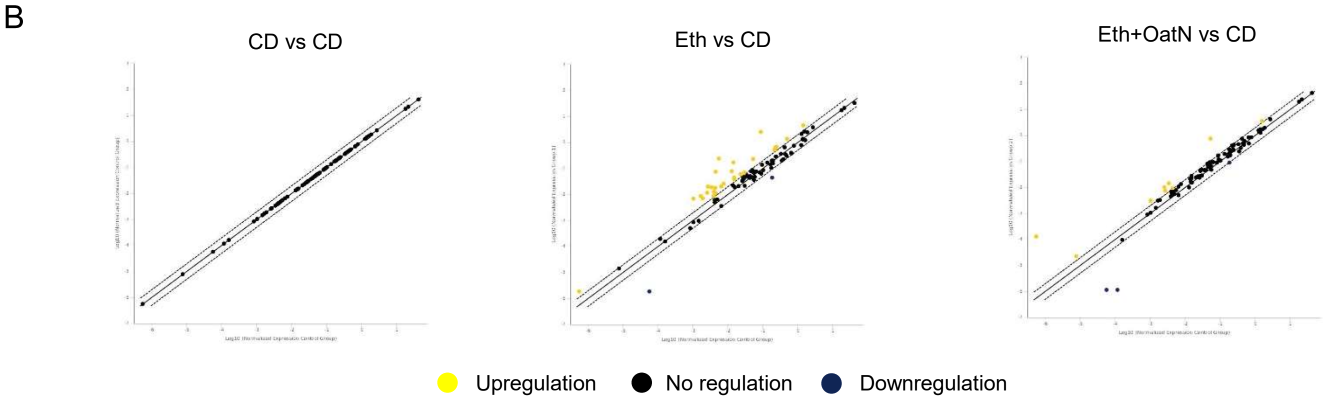
B
CD vs CD
Eth vs CD
Eth+OatN vs CD
Upregulation
No regulation
Downregulation

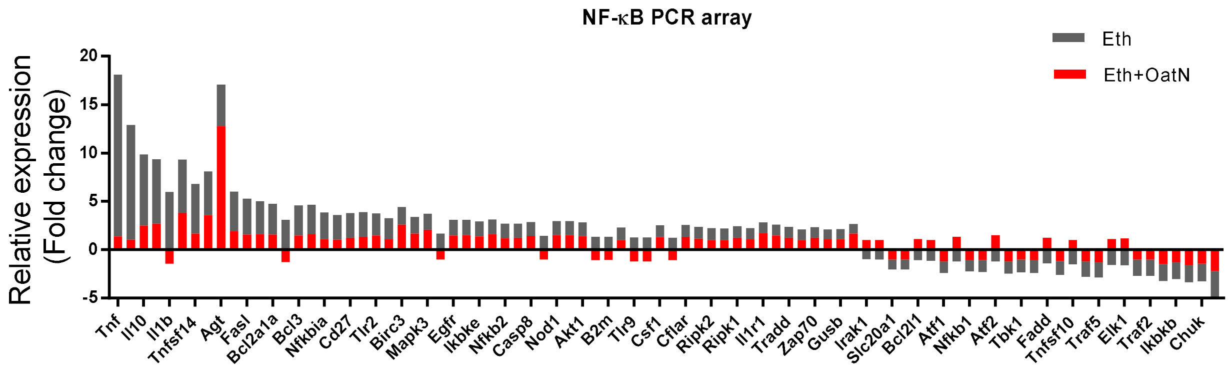
NF-κB PCR array
Eth
Eth+OatN
Relative expression
(Fold change)
20
15
10
5
0
-5
Tnf
Il10
Il1b
Tnfsf14
Agt
Fasl
Bcl2a1a
Bcl3
Nfkbia
Cd27
Tlr2
Birc3
Mapk3
Egfr
Ikbke
Nfkb2
Casp8
Nod1
Akt1
B2m
Tlr9
Csf1
Cflar
Ripk2
Ripk1
Il1r1
Tradd
Zap70
Gusb
Irak1
Slc20a1
Bcl2l1
Atf1
Nfkb1
Atf2
Tbk1
Fadd
Tnfsf10
Traf5
Elk1
Traf2
Ikbkb
Chuk

Table S1 OatN lipid profiles

Summary

			nmol per mg dry lipid wt		% total signal	
			Oat		Oat	
Mass	Compound Formula	Compound Name	ave	stdev	ave	stdev
926.6	C49H80O15	**DGDG(34:6)**	0.000	0.000	0.000	0.000
928.6	C49H82O15	**DGDG(34:5)**	0.000	0.000	0.000	0.000
930.6	C49H84O15	**DGDG(34:4)**	0.000	0.000	0.006	0.002
932.6	C49H86O15	**DGDG(34:3)**	0.003	0.001	0.445	0.069
934.6	C49H88O15	**DGDG(34:2)**	0.027	0.006	4.448	1.072
936.6	C49H90O15	**DGDG(34:1)**	0.021	0.003	3.578	1.129
954.6	C51H84O15	**DGDG(36:6)**	0.001	0.000	0.128	0.051
956.6	C51H86O15	**DGDG(36:5)**	0.008	0.002	1.378	0.303
958.6	C51H88O15	**DGDG(36:4)**	0.061	0.017	10.021	1.801
960.6	C51H90O15	**DGDG(36:3)**	0.028	0.010	4.415	0.417
962.6	C51H92O15	**DGDG(36:2)**	0.021	0.007	3.433	0.471
964.7	C51H94O15	**DGDG(36:1)**	0.003	0.001	0.408	0.006
982.6	C53H88O15	**DGDG(38:6)**	0.000	0.000	0.000	0.000
984.6	C53H90O15	**DGDG(38:5)**	0.000	0.000	0.000	0.000
986.6	C53H92O15	**DGDG(38:4)**	0.000	0.000	0.017	0.024
988.7	C53H94O15	**DGDG(38:3)**	0.000	0.001	0.063	0.052
**Total DGDG**		**Total DGDG**	**0.174**	**0.047**	**28.340**	**5.240**
764.5	C43H70O10	**MGDG(34:6)**	0.000	0.000	0.000	0.000
766.5	C43H72O10	**MGDG(34:5)**	0.000	0.000	0.000	0.000
768.5	C43H74O10	**MGDG(34:4)**	0.000	0.000	0.000	0.000
770.5	C43H76O10	**MGDG(34:3)**	0.000	0.000	0.003	0.005
772.6	C43H78O10	**MGDG(34:2)**	0.001	0.000	0.207	0.021
774.6	C43H80O10	**MGDG(34:1)**	0.001	0.000	0.175	0.090
792.5	C45H74O10	**MGDG(36:6)**	0.001	0.001	0.117	0.093
794.5	C45H76O10	**MGDG(36:5)**	0.002	0.002	0.225	0.187
796.6	C45H78O10	**MGDG(36:4)**	0.006	0.003	0.894	0.118
798.6	C45H80O10	**MGDG(36:3)**	0.003	0.002	0.420	0.094
800.6	C45H82O10	**MGDG(36:2)**	0.003	0.002	0.524	0.008
802.6	C45H84O10	**MGDG(36:1)**	0.000	0.000	0.005	0.008
820.6	C47H78O10	**MGDG(38:6)**	0.000	0.000	0.000	0.000
822.6	C47H80O10	**MGDG(38:5)**	0.000	0.000	0.000	0.000
824.6	C47H82O10	**MGDG(38:4)**	0.000	0.000	0.000	0.000
826.6	C47H84O10	**MGDG(38:3)**	0.000	0.000	0.003	0.004
**Total MGDG**		**Total MGDG**	**0.017**	**0.010**	**2.573**	**0.405**
738.5	C38H73O10P	**PG(32:1)**	0.000	0.000	0.000	0.000
740.5	C38H75O10P	**PG(32:0)**	0.008	0.004	1.240	0.028
760.5	C40H71O10P	**PG(34:4)**	0.000	0.000	0.000	0.000
762.5	C40H73O10P	**PG(34:3)**	0.000	0.000	0.071	0.026
764.5	C40H75O10P	**PG(34:2)**	0.013	0.005	2.131	0.136
766.5	C40H77O10P	**PG(34:1)**	0.005	0.002	0.778	0.044
768.5	C40H79O10P	**PG(34:0)**	0.001	0.001	0.193	0.024

784.5	C42H71O10P	**PG(36:6)**	0.000	0.000	0.000	0.000
786.5	C42H73O10P	**PG(36:5)**	0.000	0.000	0.009	0.013
788.5	C42H75O10P	**PG(36:4)**	0.002	0.001	0.312	0.028
790.5	C42H77O10P	**PG(36:3)**	0.002	0.002	0.300	0.122
792.5	C42H79O10P	**PG(36:2)**	0.001	0.000	0.156	0.027
794.6	C42H81O10P	**PG(36:1)**	0.000	0.000	0.020	0.029
**Total PG**		**Total PG**	**0.033**	**0.015**	**5.211**	**0.098**
500.3	C22H43O9P	**LPG(16:1)**	0.000	0.000	0.007	0.010
502.3	C22H45O9P	**LPG(16:0)**	0.001	0.000	0.131	0.007
524.3	C24H43O9P	**LPG(18:3)**	0.000	0.000	0.004	0.006
526.3	C24H45O9P	**LPG(18:2)**	0.000	0.000	0.023	0.007
528.3	C24H47O9P	**LPG(18:1)**	0.000	0.000	0.006	0.008
**Total LysoPG**		**Total LysoPG**	**0.001**	**0.001**	**0.172**	**0.023**
494.3	C24H48O7PN	**LPC(16:1)**	0.000	0.000	0.010	0.009
496.3	C24H50O7PN	**LPC(16:0)**	0.024	0.008	3.824	0.368
518.3	C26H48O7PN	**LPC(18:3)**	0.001	0.000	0.133	0.001
520.3	C26H50O7PN	**LPC(18:2)**	0.022	0.008	3.571	0.267
522.3	C26H52O7PN	**LPC(18:1)**	0.006	0.002	0.950	0.118
524.4	C26H54O7PN	**LPC(18:0)**	0.001	0.001	0.163	0.012
**Total LysoPC**		**Total LysoPC**	**0.054**	**0.020**	**8.651**	**0.749**
452.3	C21H42O7PN	**LPE(16:1)**	0.000	0.000	0.000	0.000
454.3	C21H44O7PN	**LPE(16:0)**	0.002	0.001	0.305	0.006
476.3	C23H42O7PN	**LPE(18:3)**	0.000	0.000	0.015	0.004
478.3	C23H44O7PN	**LPE(18:2)**	0.003	0.002	0.456	0.040
480.3	C23H46O7PN	**LPE(18:1)**	0.001	0.000	0.082	0.027
**Total LysoPE**		**Total LysoPE**	**0.006**	**0.003**	**0.858**	**0.069**
734.6	C40H80O8PN	**PC(32:0)**	0.014	0.005	2.320	0.253
754.5	C42H76O8PN	**PC(34:4)**	0.000	0.000	0.029	0.015
756.5	C42H78O8PN	**PC(34:3)**	0.003	0.002	0.519	0.026
758.6	C42H80O8PN	**PC(34:2)**	0.054	0.024	8.464	0.024
760.6	C42H82O8PN	**PC(34:1)**	0.026	0.010	4.126	0.193
778.5	C44H76O8PN	**PC(36:6)**	0.000	0.000	0.060	0.017
780.5	C44H78O8PN	**PC(36:5)**	0.004	0.002	0.665	0.038
782.6	C44H80O8PN	**PC(36:4)**	0.045	0.019	7.019	0.168
784.6	C44H82O8PN	**PC(36:3)**	0.035	0.012	5.559	0.618
786.6	C44H84O8PN	**PC(36:2)**	0.015	0.005	2.417	0.269
788.6	C44H86O8PN	**PC(36:1)**	0.002	0.001	0.317	0.024
806.6	C46H80O8PN	**PC(38:6)**	0.000	0.000	0.026	0.029
808.6	C46H82O8PN	**PC(38:5)**	0.000	0.000	0.011	0.011
810.6	C46H84O8PN	**PC(38:4)**	0.000	0.000	0.028	0.000
812.6	C46H86O8PN	**PC(38:3)**	0.001	0.000	0.187	0.042
814.6	C46H88O8PN	**PC(38:2)**	0.002	0.001	0.258	0.031
836.6	C48H86O8PN	**PC(40:5)**	0.000	0.000	0.000	0.000
838.6	C48H88O8PN	**PC(40:4)**	0.000	0.000	0.000	0.000
840.6	C48H90O8PN	**PC(40:3)**	0.000	0.000	0.006	0.009
842.7	C48H92O8PN	**PC(40:2)**	0.000	0.000	0.003	0.002
**Total PC**		**Total PC**	**0.202**	**0.081**	**32.017**	**1.508**

686.5	C37H68O8PN	**PE(32:3)**	0.000	0.000	0.002	0.003
688.5	C37H70O8PN	**PE(32:2)**	0.000	0.000	0.016	0.001
690.5	C37H72O8PN	**PE(32:1)**	0.000	0.000	0.022	0.012
692.5	C37H74O8PN	**PE(32:0)**	0.002	0.001	0.338	0.041
712.5	C39H70O8PN	**PE(34:4)**	0.000	0.000	0.006	0.004
714.5	C39H72O8PN	**PE(34:3)**	0.001	0.001	0.164	0.058
716.5	C39H74O8PN	**PE(34:2)**	0.012	0.008	1.850	0.403
718.5	C39H76O8PN	**PE(34:1)**	0.004	0.002	0.565	0.014
736.5	C41H70O8PN	**PE(36:6)**	0.000	0.000	0.008	0.004
738.5	C41H72O8PN	**PE(36:5)**	0.002	0.001	0.240	0.031
740.5	C41H74O8PN	**PE(36:4)**	0.010	0.005	1.480	0.166
742.5	C41H76O8PN	**PE(36:3)**	0.007	0.003	1.036	0.023
744.5	C41H78O8PN	**PE(36:2)**	0.001	0.001	0.211	0.030
746.6	C41H80O8PN	**PE(36:1)**	0.000	0.000	0.015	0.019
764.5	C43H74O8PN	**PE(38:6)**	0.000	0.000	0.000	0.000
766.5	C43H76O8PN	**PE(38:5)**	0.000	0.000	0.000	0.000
768.5	C43H78O8PN	**PE(38:4)**	0.000	0.000	0.008	0.004
770.6	C43H80O8PN	**PE(38:3)**	0.000	0.000	0.004	0.004
798.6	C45H84O8PN	**PE(40:3)**	0.000	0.000	0.001	0.002
800.6	C45H86O8PN	**PE(40:2)**	0.000	0.000	0.019	0.003
824.6	C47H86O8PN	**PE(42:4)**	0.000	0.000	0.000	0.000
826.6	C47H88O8PN	**PE(42:3)**	0.000	0.000	0.000	0.000
828.6	C47H90O8PN	**PE(42:2)**	0.000	0.000	0.011	0.004
**Total PE**		**Total PE**	**0.039**	**0.022**	**5.997**	**0.747**
822.5	C41H73O13P	**PI(32:3)**	0.000	0.000	0.000	0.000
824.5	C41H75O13P	**PI(32:2)**	0.000	0.000	0.024	0.009
826.5	C41H77O13P	**PI(32:1)**	0.000	0.000	0.026	0.005
828.5	C41H79O13P	**PI(32:0)**	0.001	0.000	0.097	0.023
848.5	C43H75O13P	**PI(34:4)**	0.000	0.000	0.002	0.003
850.5	C43H77O13P	**PI(34:3)**	0.002	0.001	0.241	0.018
852.5	C43H79O13P	**PI(34:2)**	0.020	0.009	3.179	0.029
854.5	C43H81O13P	**PI(34:1)**	0.005	0.002	0.860	0.105
872.5	C45H75O13P	**PI(36:6)**	0.000	0.000	0.005	0.004
874.5	C45H77O13P	**PI(36:5)**	0.000	0.000	0.049	0.005
876.5	C45H79O13P	**PI(36:4)**	0.003	0.001	0.465	0.055
878.5	C45H81O13P	**PI(36:3)**	0.003	0.001	0.442	0.089
880.6	C45H83O13P	**PI(36:2)**	0.002	0.001	0.242	0.011
882.6	C45H85O13P	**PI(36:1)**	0.000	0.000	0.010	0.005
**Total PI**		**Total PI**	**0.036**	**0.015**	**5.641**	**0.196**
756.5	C40H70O10PN	**PS(34:4)**	0.000	0.000	0.000	0.000
758.5	C40H72O10PN	**PS(34:3)**	0.000	0.000	0.009	0.002
760.5	C40H74O10PN	**PS(34:2)**	0.001	0.001	0.128	0.048
762.5	C40H76O10PN	**PS(34:1)**	0.000	0.000	0.005	0.007
780.5	C42H70O10PN	**PS(36:6)**	0.000	0.000	0.000	0.000
782.5	C42H72O10PN	**PS(36:5)**	0.000	0.000	0.000	0.000
784.5	C42H74O10PN	**PS(36:4)**	0.000	0.000	0.031	0.003
786.5	C42H76O10PN	**PS(36:3)**	0.000	0.000	0.029	0.000

788.5	C42H78O10PN	**PS(36:2)**	0.000	0.000	0.025	0.027
790.6	C42H80O10PN	**PS(36:1)**	0.000	0.000	0.000	0.000
808.5	C44H74O10PN	**PS(38:6)**	0.000	0.000	0.000	0.000
810.5	C44H76O10PN	**PS(38:5)**	0.000	0.000	0.000	0.000
812.5	C44H78O10PN	**PS(38:4)**	0.000	0.000	0.000	0.000
814.6	C44H80O10PN	**PS(38:3)**	0.000	0.000	0.005	0.006
816.6	C44H82O10PN	**PS(38:2)**	0.000	0.000	0.044	0.008
818.6	C44H84O10PN	**PS(38:1)**	0.000	0.000	0.000	0.000
840.6	C46H82O10PN	**PS(40:4)**	0.000	0.000	0.000	0.000
842.6	C46H84O10PN	**PS(40:3)**	0.000	0.000	0.006	0.003
844.6	C46H86O10PN	**PS(40:2)**	0.001	0.001	0.141	0.017
846.6	C46H88O10PN	**PS(40:1)**	0.000	0.000	0.004	0.006
868.6	C48H86O10PN	**PS(42:4)**	0.000	0.000	0.000	0.000
870.6	C48H88O10PN	**PS(42:3)**	0.000	0.000	0.011	0.002
872.6	C48H90O10PN	**PS(42:2)**	0.001	0.000	0.202	0.071
874.6	C48H92O10PN	**PS(42:1)**	0.000	0.000	0.023	0.004
898.6	C50H92O10PN	**PS(44:3)**	0.000	0.000	0.038	0.009
900.7	C50H94O10PN	**PS(44:2)**	0.000	0.000	0.060	0.008
**Total PS**		**Total PS**	**0.005**	**0.003**	**0.761**	**0.056**
666.5	C35H69O8P	**PA(32:0)**	0.001	0.001	0.094	0.043
682.4	C37H61O8P	**PA(34:6)**	0.000	0.000	0.000	0.000
684.4	C37H63O8P	**PA(34:5)**	0.000	0.000	0.004	0.006
686.4	C37H65O8P	**PA(34:4)**	0.000	0.000	0.000	0.000
688.5	C37H67O8P	**PA(34:3)**	0.002	0.002	0.263	0.253
690.5	C37H69O8P	**PA(34:2)**	0.034	0.037	4.475	3.802
692.5	C37H71O8P	**PA(34:1)**	0.004	0.003	0.646	0.202
710.4	C39H65O8P	**PA(36:6)**	0.000	0.000	0.003	0.004
712.5	C39H67O8P	**PA(36:5)**	0.002	0.002	0.247	0.168
714.5	C39H69O8P	**PA(36:4)**	0.016	0.015	2.294	1.304
716.5	C39H71O8P	**PA(36:3)**	0.008	0.005	1.141	0.268
718.5	C39H73O8P	**PA(36:2)**	0.004	0.003	0.614	0.265
**Total PA**		**Total PA**	**0.071**	**0.068**	**9.780**	**6.295**
**Total**		**Total**	**0.639**	**0.282**	**100.000**	**0.000**

Table S2 Analysis for monosaccaride consitutes in oatN dervied polysaccharide

	weight (ug)	mole%
**Arabinose (Ara)**	3.2	8.7
**Xylose (Xyl)**	2.5	6.7
**Mannose (Man)**	0.1	0.1
**Galactose (Gal)**	0.3	0.7
**Glucose (Glc)**	37.2	83.8
**SUM**	43.2	100
**% CHO**	21.60%	

Table S3 Analysis for residues in oatN dervied polysaccharide

Residue Abbr.	Residue	% Area
**t-Ara*f***	terminal arabinofuranosyl	9.6
**t-Xyl**	terminal xylopyranosyl	1.1
**2-Ara*f***	2-linked arabinofuranosyl	0.5
**t-Man**	terminal mannopyranosyl	1.1
**t-Glc**	terminal glucopyranosyl	2.7
**t-Gal**	terminal galactopyranosyl	0.3
**4 or 5-Ara*f***	4- or 5-linked arabinofuranosyl	0.8
**4-Xyl**	4-linked xylopyranosyl	1.3
**3-Glc**	3-linked glucopyranosyl	32.4
**2-Man**	2-linked mannopyranosyl	1.7
**3-Gal**	3-linked galactopyranosyl	0.2
**4-Man**	4-linked mannopyranosyl	0.6
**6-Man**	6-linked mannopyranosyl	0.2
**6-Glc**	6-linked glucopyranosyl	0.5
**4-Glc**	4-linked glucopyranosyl	42.1
**2,4-Xyl**	2,4-linked xylopyranosyl	0.3
**3,4-Man**	3,4-linked mannopyranosyl	0.2
**6-Gal**	6-linked galactopyranosyl	0.3
**3,4-Glc**	3,4-linked glucopyranosyl	0.1
**2,3-Glc**	2,3-linked glucopyranosyl	0.2
**2,4-Glc**	2,4-linked glucopyranosyl	0.2
**3,6-Glc**	3,6-linked glucopyranosyl	0.2
**3,6-Man**	3,6-linked mannopyranosyl	0.2
**2,6-Man**	2,6-linked mannopyranosyl	1.2
**4,6-Glc**	4,6-linked glucopyranosyl	0.5
**3,6-Gal**	3,6-linked galactopyranosyl	1.2

Table S4 Murine microglia cells pulled down by oatN& oatM (MS/MS)

Description	Gene Name	oatN Abundances	oatM Abundances
Actin, cytoplasmic 1	Actb	84394349	105821415
Sodium/potassium-transporting ATPase subunit alpha-3	Atp1a3	161626389	182068633
Tubulin beta-2A chain	Tubb2a	115629935	121820084
Tubulin beta-2B chain	Tubb2b	385744	2517809
Tubulin beta-4B chain	Tubb4b	26302466	19833884
Tubulin beta-4A chain	Tubb4a	10535568	9846378
ATP synthase subunit beta, mitochondrial	Atp5f1b	41371300	33643266
Tubulin beta-5 chain	Tubb5	6032577	6896881
Clathrin heavy chain 1	Cltc	38423852	56781623
Sodium/potassium-transporting ATPase subunit alpha-2	Atp1a2	20478391	17180218
Tubulin alpha-1A chain	Tuba1a	144402486	152410864
Sodium/potassium-transporting ATPase subunit alpha-1	Atp1a1	16946572	20795448
Excitatory amino acid transporter 2	Slc1a2	31265042	27623436
Tubulin alpha-4A chain	Tuba4a	17561433	14013838
Tubulin beta-3 chain	Tubb3	6532915	6679664
V-type proton ATPase 16 kDa proteolipid subunit	Atp6v0c	383346	392557
ATP synthase subunit alpha, mitochondrial	Atp5f1a	60219726	52606235
Actin, alpha cardiac muscle 1	Actc1	9674630	13225590
Isoform 2 of Syntaxin-binding protein 1	Stxbp1	26599607	26678984
Syntaxin-binding protein 1	Stxbp1	740853	729414
Spectrin beta chain, non-erythrocytic 1	Sptbn1	10498340	18120515
Tubulin beta-6 chain	Tubb6	303931	252863
ATP synthase F(0) complex subunit C1, mitochondrial	Atp5mc1	250303	133831
Spectrin alpha chain, non-erythrocytic 1	Sptan1	7698121	18312862
ADP/ATP translocase 1	Slc25a4	37856359	38332243
Neurofilament light polypeptide	Nefl	25707406	18678736
Hexokinase-1	Hk1	9516963	5508208
Glyceraldehyde-3-phosphate dehydrogenase	Gapdh	39274442	24075407
2',3'-cyclic-nucleotide 3'-phosphodiesterase	Cnp	35596711	34134842
Synaptophysin	Syp	6554917	6874024
Calcium-binding mitochondrial carrier protein Aralar1	Slc25a12	7398361	5810974
Vesicle-fusing ATPase	Nsf	12601753	14813494
ADP/ATP translocase 2	Slc25a5	8074521	7287625

AP-2 complex subunit beta	Ap2b1	4330923	6721677
Calcium/calmodulin-dependent protein kinase type II subunit alpha	Camk2a	15045897	12987995
Voltage-dependent anion-selective channel protein 1	Vdac1	37137151	39277660
Cytoplasmic dynein 1 heavy chain 1	Dync1h1	1951948	2680893
Isoform 3 of Dynamin-1	Dnm1	12078610	20630427
Alpha-internexin	Ina	12660064	9962798
Synaptotagmin-1	Syt1	9615340	11059876
Plasma membrane calcium-transporting ATPase 2	Atp2b2	7009460	9561212
Guanine nucleotide-binding protein G(o) subunit alpha	Gnao1	19482262	21633353
Cytochrome b-c1 complex subunit 2, mitochondrial	Uqcrc2	9498412	7840935
Isoform Alpha-2 of Guanine nucleotide-binding protein G(o) subunit alpha	Gnao1	71189	49801
Plasma membrane calcium-transporting ATPase 1	Atp2b1	2008318	3124359
Sodium/potassium-transporting ATPase subunit beta-1	Atp1b1	33126483	37728383
Guanine nucleotide-binding protein G(I)/G(S)/G(T) subunit beta-1	Gnb1	10214413	10643355
Isoform A1-I of V-type proton ATPase 116 kDa subunit a isoform 1	Atp6v0a1	6577972	7813179
V-type proton ATPase catalytic subunit A	Atp6v1a	4254142	6063723
Guanine nucleotide-binding protein G(I)/G(S)/G(T) subunit beta-2	Gnb2	10384806	11881027
Guanine nucleotide-binding protein G(i) subunit alpha-1	Gnai1	1132125	6613960
Synapsin-1	Syn1	11502472	14946767
Excitatory amino acid transporter 1	Slc1a3	8152380	8353494
AP-2 complex subunit alpha-1	Ap2a1	2834413	5286102
V-type proton ATPase subunit B, brain isoform	Atp6v1b2	4001323	8640029
NADH-ubiquinone oxidoreductase 75 kDa subunit, mitochondrial	Ndufs1	4452872	4314291
Guanine nucleotide-binding protein G(i) subunit alpha-2	Gnai2	5894171	884483
Cytochrome b-c1 complex subunit 1, mitochondrial	Uqcrc1	8022614	7450435
Contactin-1	Cntn1	4920349	6440290
Heat shock cognate 71 kDa protein	Hspa8	3234151	4847257
Serine/threonine-protein phosphatase 2B catalytic subunit alpha isoform	Ppp3ca	3373152	2912804
Phosphate carrier protein, mitochondrial	Slc25a3	11505112	8698996
AP-2 complex subunit mu	Ap2m1	1285307	2812923
Synaptic vesicle glycoprotein 2A	Sv2a	2330603	2688406
Isoform 4 of Myelin basic protein	Mbp	18567498	20812581
Synapsin-2	Syn2	5154521	3063708
Neural cell adhesion molecule 1	Ncam1	3756882	7698069
Isoform IIb of Synapsin-2	Syn2	185887	nd
MIC complex subunit Mic60	Immt	2542246	1753431

Myelin proteolipid protein	Plp1	27075304	26096946
Syntaxin-1B	Stx1b	9082801	8694240
Isocitrate dehydrogenase [NAD] subunit alpha, mitochondrial	Idh3a	5319177	4683208
Ras-related protein Rab-3A	Rab3a	5063074	10804296
Tenascin-R	Tnr	1128491	800543
Solute carrier family 12 member 5	Slc12a5	3569345	3915712
Sarcoplasmic/endoplasmic reticulum calcium ATPase 2	Atp2a2	3032811	4763071
AP-2 complex subunit alpha-2	Ap2a2	871287	2589785
NADH dehydrogenase [ubiquinone] 1 alpha subcomplex subunit 9, mitochonc	Ndufa9	1665455	1758962
Succinate dehydrogenase [ubiquinone] flavoprotein subunit, mitochondrial	Sdha	2356823	2158870
Isoform 2 of Ankyrin-2	Ank2	2193907	3464604
Neurofilament medium polypeptide	Nefm	6984675	6820079
Voltage-dependent anion-selective channel protein 2	Vdac2	12760180	12312103
Calcium/calmodulin-dependent protein kinase type II subunit beta	Camk2b	2425126	2085514
Electrogenic sodium bicarbonate cotransporter 1	Slc4a4	1582220	1815445
ATP synthase subunit gamma, mitochondrial	Atp5f1c	5234839	4538316
AP-1 complex subunit beta-1	Ap1b1	417435	546732
ATP synthase F(0) complex subunit B1, mitochondrial	Atp5pb	1976713	2279158
Ras-related protein Rab-3C	Rab3c	2711335	2493508
S-adenosylhomocysteine hydrolase-like protein 1	Ahcyl1	2687896	2041017
Histone H2A type 2-C	Hist2h2ac	20310	139331
Cytochrome b-c1 complex subunit 8	Uqcrq	698002	558141
Synaptic vesicle glycoprotein 2B	Sv2b	815907	987577
Sodium/potassium-transporting ATPase subunit beta-2	Atp1b2	8730124	7617246
Alpha-actinin-1	Actn1	1728925	3455518
Dihydropyrimidinase-related protein 2	Dpysl2	1548939	1164901
Mitochondrial 2-oxoglutarate/malate carrier protein	Slc25a11	3338974	2383841
Glial fibrillary acidic protein	Gfap	4126028	2535069
NADH dehydrogenase [ubiquinone] flavoprotein 1, mitochondrial	Ndufv1	2968451	3104428
4F2 cell-surface antigen heavy chain	Slc3a2	2231131	3765427
Protein kinase C gamma type	Prkcg	2609193	1995625
Heat shock protein HSP 90-beta	Hsp90ab1	1943894	986021
Guanine nucleotide-binding protein G(i) subunit alpha	Gnai3	nd	nd
Synaptogyrin-3	Syngr3	1469551	1581413
Heat shock 70 kDa protein 12A	Hspa12a	1022992	1341434
Cytochrome c1, heme protein, mitochondrial	Cyc1	3831568	5636917

ATP synthase subunit g, mitochondrial	Atp5mg	2070106	1914826
14-3-3 protein zeta/delta	Ywhaz	1674223	2094555
Cytochrome c oxidase subunit 4 isoform 1, mitochondrial	Cox4i1	3750879	5745413
Heterogeneous nuclear ribonucleoprotein L	Hnrnpl	81207	848826
Voltage-dependent anion-selective channel protein 3	Vdac3	6365102	5250046
ATP synthase subunit d, mitochondrial	Atp5pd	2788081	2624236
Adenylyl cyclase-associated protein 1	Cap1	384054	1478747
Synaptosomal-associated protein 25	Snap25	5542544	6493296
Microtubule-associated protein 1B	Map1b	1507427	392284
V-type proton ATPase subunit d 1	Atp6v0d1	2904975	2293820
Sideroflexin-3	Sfxn3	1258286	1005763
Matrin-3	Matr3	761707	1373491
NAD-dependent protein deacetylase sirtuin-2	Sirt2	2186855	2045713
Creatine kinase U-type, mitochondrial	Ckmt1	278364	705922
Microtubule-associated protein 6	Map6	2474178	2103003
Calcium/calmodulin-dependent protein kinase type II subunit delta	Camk2d	nd	nd
ADP-ribosylation factor 1	Arf1	1309919	1603038
ATP synthase subunit O, mitochondrial	Atp5po	4744343	4736175
Neurochondrin	Ncdn	624075	787043
Putative adenosylhomocysteinase 3	Ahcyl2	nd	47447
Neurofilament heavy polypeptide	Nefh	2655941	2004317
Mitochondrial glutamate carrier 1	Slc25a22	1623409	2522511
NADH dehydrogenase [ubiquinone] 1 alpha subcomplex subunit 10, mitochor	Ndufa10	1928303	1563074
Neuroplastin	Nptn	4291899	4673178
NADH dehydrogenase [ubiquinone] iron-sulfur protein 3, mitochondrial	Ndufs3	1703481	1531387
Plasma membrane calcium-transporting ATPase 4	Atp2b4	239886	397741
Inositol 1,4,5-trisphosphate receptor type 1	Itpr1	208367	966681
Dihydrolipoyllysine-residue acetyltransferase component of pyruvate dehydro	Dlat	1195805	1215454
Dynamin-3	Dnm3	nd	nd
Synaptotagmin-2	Syt2	413245	137014
Calcium/calmodulin-dependent protein kinase type II subunit gamma	Camk2g	104488	70130
Synaptogyrin-1	Syngr1	2576430	2253856
Alpha-centractin	Actr1a	949890	359215
Ras-related protein Rab-2A	Rab2a	1564145	2270105
Cytochrome c oxidase subunit 2	Mtco2	2150028	2352231
Isoform Beta-II of Protein kinase C beta type	Prkcb	483599	1070557

Beta-soluble NSF attachment protein	Napb	1174480	1930478
Protein NDRG2	Ndrg2	2052349	1528472
Serine/threonine-protein phosphatase 2A 65 kDa regulatory subunit A alpha i	Ppp2r1a	814626	936691
Beta-centractin	Actr1b	246121	nd
Heat shock protein HSP 90-alpha	Hsp90aa1	294969	1736316
Sodium- and chloride-dependent GABA transporter 3	Slc6a11	2611970	2211608
Isoform 2 of Tyrosine-protein phosphatase non-receptor type substrate 1	Sirpa	2385076	2875037
V-type proton ATPase subunit H	Atp6v1h	602777	977982
Glycerol-3-phosphate dehydrogenase, mitochondrial	Gpd2	1167912	1423696
14-3-3 protein gamma	Ywhag	266336	539036
Guanine nucleotide-binding protein G(q) subunit alpha	Gnaq	561933	641753
Elongation factor 1-alpha 1	Eef1a1	2520272	2233819
NADH dehydrogenase [ubiquinone] 1 beta subcomplex subunit 4	Ndufb4	59481	472229
Dynamin-1-like protein	Dnm1l	181066	758915
Vesicular glutamate transporter 1	Slc17a7	1318281	1565988
Sodium-dependent neutral amino acid transporter SLC6A17	Slc6a17	159884	705637
Guanine nucleotide-binding protein G(z) subunit alpha	Gnaz	432860	474073
Neurofascin	Nfasc	1223642	772120
60 kDa heat shock protein, mitochondrial	Hspd1	665900	1150058
Tyrosine-protein phosphatase non-receptor type substrate 1	Sirpa	nd	nd
V-type proton ATPase subunit E 1	Atp6v1e1	358517	590701
Ras-related protein Rap-1A	Rap1a	949127	1005211
Amphiphysin	Amph	257900	1335658
Syntaxin-1A	Stx1a	1503410	1227318
Sodium- and chloride-dependent GABA transporter 1	Slc6a1	2528299	2867319
Vesicle-associated membrane protein 2	Vamp2	2814290	3478064
Cytochrome b-c1 complex subunit Rieske, mitochondrial	Uqcrfs1	878749	954356
Leucine-rich glioma-inactivated protein 1	Lgi1	529821	590662
Dipeptidyl aminopeptidase-like protein 6	Dpp6	997143	1267241
Immunoglobulin superfamily member 8	Igsf8	902581	485229
Prohibitin-2	Phb2	1312705	1365769
Isoform 8 of Band 4.1-like protein 3	Epb41l3	892805	1066548
Alpha-enolase	Eno1	455690	nd
Microtubule-associated protein 1A	Map1a	290329	481848
Plectin	Plec	59225	338060
Heterogeneous nuclear ribonucleoproteins A2/B1	Hnrnpa2b1	661026	4172038

Mitochondrial carrier homolog 2	Mtch2	895675	1505218
ATP-dependent 6-phosphofructokinase, muscle type	Pfkm	706420	90850
ATP-sensitive inward rectifier potassium channel 10	Kcnj10	358765	564160
Pyruvate dehydrogenase E1 component subunit beta, mitochondrial	Pdhb	2029642	1151524
Myc box-dependent-interacting protein 1	Bin1	969981	1274017
Cell adhesion molecule 2	Cadm2	683872	757557
Neuronal membrane glycoprotein M6-a	Gpm6a	6984661	7844213
Phospholipid-transporting ATPase IA	Atp8a1	147765	170919
Sodium/calcium exchanger 2	Slc8a2	1150081	1307374
Alpha-adducin	Add1	1117509	1511897
Contactin-associated protein 1	Cntnap1	351282	162985
Septin-7	Septin7	901392	1385710
Phospholipid phosphatase 3	Plpp3	568064	434413
Neuronal membrane glycoprotein M6-b	Gpm6b	2178921	2176011
Cell adhesion molecule 3	Cadm3	946362	1345709
Endophilin-A1	Sh3gl2	1337490	1199133
Dynamin-2	Dnm2	391367	651934
Cytochrome c oxidase subunit 6B1	Cox6b1	3515260	4258121
Glyceraldehyde-3-phosphate dehydrogenase, testis-specific	Gapdhs	6468281	4276290
Keratin, type I cytoskeletal 10	Krt10	5428762	4153328
NADH dehydrogenase [ubiquinone] iron-sulfur protein 2, mitochondrial	Ndufs2	691515	588527
Mitochondrial import receptor subunit TOM70	Tomm70	428428	461925
Keratin, type II cytoskeletal 1	Krt1	4948854	4023100
ATP-dependent 6-phosphofructokinase, platelet type	Pfkp	245670	118977
Protein NipSnap homolog 2	Nipsnap2	445544	156628
Rho-related GTP-binding protein RhoB	Rhob	344483	517277
Synaptic vesicle membrane protein VAT-1 homolog	Vat1	73638	63059
TAR DNA-binding protein 43	Tardbp	261683	414952
CDGSH iron-sulfur domain-containing protein 1	Cisd1	1105276	747378
Glutaminase kidney isoform, mitochondrial	Gls	124395	122177
14-3-3 protein beta/alpha	Ywhab	nd	134778
Heterogeneous nuclear ribonucleoprotein H	Hnrnph1	534377	2209614
Ras-related C3 botulinum toxin substrate 1	Rac1	2019362	3234196
Hippocalcin-like protein 4	Hpcal4	370802	651477
Sideroflexin-1	Sfxn1	160196	355884
Ras-related protein Rab-3B	Rab3b	1074273	230350

Pyruvate carboxylase, mitochondrial	Pc	302799	142382
Sideroflexin-5	Sfxn5	550668	564813
Calcium-dependent secretion activator 1	Cadps	211701	554398
Vesicular inhibitory amino acid transporter	Slc32a1	613731	nd
D-beta-hydroxybutyrate dehydrogenase, mitochondrial	Bdh1	666559	2129649
Cytochrome c oxidase subunit 5A, mitochondrial	Cox5a	1191956	1268366
Isoform 3 of Reticulon-3	Rtn3	1632123	861417
DmX-like protein 2	Dmxl2	362330	699765
14-3-3 protein theta	Ywhaq	342648	380458
Importin subunit beta-1	Kpnb1	nd	474984
Pyruvate dehydrogenase E1 component subunit alpha, somatic form, mitochc	Pdha1	985909	532925
Thy-1 membrane glycoprotein	Thy1	6435239	7260032
NADH dehydrogenase [ubiquinone] 1 alpha subcomplex subunit 8	Ndufa8	956997	822839
Clathrin coat assembly protein AP180	Snap91	2035603	2911314
Histone H2A type 3	Hist3h2a	1308297	4245771
Trifunctional enzyme subunit alpha, mitochondrial	Hadha	390865	275321
Septin-5	Septin5	671633	413300
Isoform 3 of Neuroplastin	Nptn	75211	67300
3-hydroxyacyl-CoA dehydrogenase type-2	Hsd17b10	107768	nd
Dynamin-like 120 kDa protein, mitochondrial	Opa1	368613	293349
Lamin-B1	Lmnb1	nd	806355
Isoform 2 of Synaptoporin	Synpr	280514	nd
Glutamine synthetase	Glul	412293	301642
Long-chain-fatty-acid--CoA ligase 6	Acsl6	346139	704981
Reticulon-4	Rtn4	438736	905150
CaM kinase-like vesicle-associated protein	Camkv	196042	536865
Ras-related protein Ral-A	Rala	519955	567313
CD81 antigen	Cd81	2757174	2808306
DnaJ homolog subfamily C member 5	Dnajc5	650537	406005
Heterogeneous nuclear ribonucleoprotein U	Hnrnpu	314559	2398071
Sodium-driven chloride bicarbonate exchanger	Slc4a10	109869	121983
Glycogen phosphorylase, brain form	Pygb	76007	10
NADH dehydrogenase [ubiquinone] iron-sulfur protein 4, mitochondrial	Ndufs4	195173	nd
Ras-related protein Rab-1A	Rab1A	1004907	937499
Heterogeneous nuclear ribonucleoprotein K	Hnrnpk	70051	332583
Gap junction alpha-1 protein	Gja1	248914	192273

Drebrin	Dbn1	1974676	2537120
Solute carrier family 2, facilitated glucose transporter member 3	Slc2a3	412653	419249
Cell division control protein 42 homolog	Cdc42	744006	1185833
Succinate--CoA ligase [ADP-forming] subunit beta, mitochondrial	Sucla2	653425	308826
AP2-associated protein kinase 1	Aak1	nd	151587
Keratin, type II cytoskeletal 5	Krt5	1442685	1019951
Hemoglobin subunit beta-1	Hbb-b1	792958	479627
Vimentin	Vim	345289	722130
Dihydrolipoyl dehydrogenase, mitochondrial	Dld	160038	31373
Brain acid soluble protein 1	Basp1	1434664	2016398
Ras-related protein Rab-14	Rab14	269955	674687
Ubiquitin-60S ribosomal protein L40	Uba52	2712634	2637535
Prohibitin	Phb	213256	635142
Myelin-oligodendrocyte glycoprotein	Mog	1211990	1179420
Calnexin	Canx	560361	1713602
Heterogeneous nuclear ribonucleoprotein A3	Hnrnpa3	509024	1347604
GTPase KRas	Kras	419231	10
NADH dehydrogenase [ubiquinone] 1 beta subcomplex subunit 10	Ndufb10	874955	780676
Isoform 1 of Cell division control protein 42 homolog	Cdc42	378331	1185833
Neurotrimin	Ntm	731039	1515818
Disks large homolog 1	Dlg1	616772	964101
NADH dehydrogenase [ubiquinone] flavoprotein 2, mitochondrial	Ndufv2	1068034	1026079
ATP synthase subunit a	Mtatp6	nd	nd
Catechol O-methyltransferase domain-containing protein 1	Comtd1	9627	7392
Succinate dehydrogenase [ubiquinone] iron-sulfur subunit, mitochondrial	Sdhb	682770	912155
NADH dehydrogenase [ubiquinone] 1 alpha subcomplex subunit 6	Ndufa6	763658	589734
Calcineurin subunit B type 1	Ppp3r1	83809	76324
Visinin-like protein 1	Vsnl1	751064	1022117
Amine oxidase [flavin-containing] B	Maob	315047	26206
Tropomodulin-2	Tmod2	175034	215299
Elongation factor Tu, mitochondrial	Tufm	392274	707570
Synaptojanin-1	Synj1	212806	69599
Guanine nucleotide-binding protein subunit alpha-14	Gna14	22457	nd
F-actin-capping protein subunit beta	Capzb	289348	160758
Protein kinase C and casein kinase substrate in neurons protein 1	Pacsin1	nd	nd
Splicing factor, proline- and glutamine-rich	Sfpq	99955	306301

Microsomal glutathione S-transferase 3	Mgst3	494378	684304
Voltage-gated potassium channel subunit beta-2	Kcnab2	211692	186538
Mitochondrial glutamate carrier 2	Slc25a18	37541	50529
Myosin-10	Myh10	135418	178631
Trifunctional enzyme subunit beta, mitochondrial	Hadhb	53793	196065
Ras-related protein Rab-10	Rab10	266352	1205073
Large neutral amino acids transporter small subunit 1	Slc7a5	201888	231202
Histone H2A.V	H2afv	19158	30354
Ras-related protein Rab-1B	Rab1b	237911	936792
Limbic system-associated membrane protein	Lsamp	739287	1479769
Keratin, type I cytoskeletal 13	Krt13	276080	nd
Histone H4	H4c1	1937595	3451822
NADH dehydrogenase [ubiquinone] iron-sulfur protein 7, mitochondrial	Ndufs7	303540	283347
Reticulon-1	Rtn1	547095	1327467
Protein kinase C epsilon type	Prkce	nd	nd
Succinate--CoA ligase [ADP/GDP-forming] subunit alpha, mitochondrial	Suclg1	813112	330888
Transforming protein RhoA	Rhoa	240717	124610
Glutamate decarboxylase 1	Gad1	201131	nd
Guanine nucleotide-binding protein G(s) subunit alpha isoforms short	Gnas	355044	329559
Hemoglobin subunit alpha	Hba	648248	270570
Ras-related protein Rab-35	Rab35	625423	124438
Keratin, type II cytoskeletal 2 epidermal	Krt2	416774	598352
Neuromodulin	Gap43	219763	631617
T-complex protein 1 subunit zeta	Cct6a	15538	52622
NADH dehydrogenase [ubiquinone] iron-sulfur protein 8, mitochondrial	Ndufs8	676322	563413
Dolichyl-diphosphooligosaccharide--protein glycosyltransferase subunit 2	Rpn2	23606	128786
Glutamate decarboxylase 2	Gad2	501419	578752
MIC complex subunit Mic19	Chchd3	214195	226410
Glutamate receptor 2	Gria2	87026	187078
Rabphilin-3A	Rph3a	521850	788152
Isoform 3 of Catenin alpha-2	Ctnna2	84002	106210
Isoform 1 of Endophilin-B2	Sh3glb2	28714	155215
Adenylyl cyclase-associated protein 2	Cap2	145957	210458
Basigin	Bsg	226614	819460
Ras-related protein Rab-6A	Rab6a	3795720	267828
Dual specificity mitogen-activated protein kinase kinase 1	Map2k1	113874	187733

Disks large homolog 4	Dlg4	133441	330288
Solute carrier family 2, facilitated glucose transporter member 1	Slc2a1	388511	555337
Saccharopine dehydrogenase-like oxidoreductase	Sccpdh	99699	179945
Ras-related protein Rab-11A	Rab11a	435770	9662
Tetratricopeptide repeat protein 7B	Ttc7b	nd	nd
Ras-related protein Rab-6B	Rab6b	48320	nd
Ras-related protein Rap-2b	Rap2b	nd	232877
Isocitrate dehydrogenase [NAD] subunit gamma 1, mitochondrial	Idh3g	1037854	463882
Septin-11	Septin11	98770	10
Disintegrin and metalloproteinase domain-containing protein 22	Adam22	434049	213352
NADH dehydrogenase [ubiquinone] iron-sulfur protein 6, mitochondrial	Ndufs6	550638	359944
MIC complex subunit Mic26	Apoo	538310	633161
Glutamate receptor 3	Gria3	198112	34262
MIC complex subunit MIC13	Micos13	45869	nd
Vesicle-associated membrane protein 1	Vamp1	441080	10
Cofilin-1	Cfl1	625235	726523
NADH dehydrogenase [ubiquinone] 1 alpha subcomplex subunit 11	Ndufa11	787377	728630
Claudin-11	Cldn11	7521	nd
Cullin-associated NEDD8-dissociated protein 1	Cand1	75192	329361
EH domain-containing protein 3	Ehd3	26882	12232
Protein NDRG1	Ndrg1	600480	365086
ATP synthase subunit delta, mitochondrial	Atp5f1d	776342	1007825
Alpha-soluble NSF attachment protein	Napa	114133	133813
CD166 antigen	Alcam	113387	352869
14-3-3 protein epsilon	Ywhae	nd	nd
AP-2 complex subunit sigma	Ap2s1	473347	620132
T-complex protein 1 subunit epsilon	Cct5	27454	34292
Isoform 2E of Voltage-dependent calcium channel subunit alpha-2/delta-1	Cacna2d1	73169	271927
NADH dehydrogenase [ubiquinone] 1 beta subcomplex subunit 7	Ndufb7	356509	10
WD repeat-containing protein 7	Wdr7	nd	447531
Guanine nucleotide-binding protein subunit beta-5	Gnb5	182707	187995
Leukocyte surface antigen CD47	Cd47	570790	626010
G protein-regulated inducer of neurite outgrowth 1	Gprin1	77607	91423
Cytochrome c oxidase subunit NDUFA4	Ndufa4	793635	922348
Guanine nucleotide-binding protein subunit alpha-13	Gna13	65855	71051
V-type proton ATPase subunit C 1	Atp6v1c1	182138	192626

Secretory carrier-associated membrane protein 1	Scamp1	130536	525619
Contactin-associated protein-like 2	Cntnap2	18881	nd
Synaptophysin-like protein 1	Sypl1	14102	6022
Disintegrin and metalloproteinase domain-containing protein 23	Adam23	175083	164265
NADH dehydrogenase [ubiquinone] 1 alpha subcomplex subunit 12	Ndufa12	197849	585509
Sodium-dependent dopamine transporter	Slc6a3	nd	nd
Ras/Rap GTPase-activating protein SynGAP	Syngap1	6877	114256
Monoglyceride lipase	Mgll	474817	742199
Monocarboxylate transporter 1	Slc16a1	nd	nd
Secretory carrier-associated membrane protein 5	Scamp5	869047	1039932
Cytochrome c oxidase subunit 8A, mitochondrial	Cox8a	nd	nd
40S ribosomal protein S3	Rps3	573805	778342
Beta-adducin	Add2	nd	351943
Atlastin-1	Atl1	43001	120598
Stress-70 protein, mitochondrial	Hspa9	263252	nd
Tumor protein p63-regulated gene 1-like protein	Tprg1l	nd	nd
Tyrosine-protein kinase Fyn	Fyn	62714	6322
C-terminal-binding protein 1	Ctbp1	138877	124718
Protein tweety homolog 1	Ttyh1	nd	176582
EF-hand domain-containing protein D2	Efhd2	138167	132008
Keratin, type II cytoskeletal 79	Krt79	407680	474258
Protein lifeguard 1	Grina	nd	nd
NADH dehydrogenase [ubiquinone] 1 alpha subcomplex subunit 2	Ndufa2	505204	689733
Cytochrome b-c1 complex subunit 9	Uqcr10	625258	603095
Long-chain-fatty-acid--CoA ligase ACSBG1	Acsbg1	120651	254267
U5 small nuclear ribonucleoprotein 200 kDa helicase	Snrnp200	nd	nd
ATP-dependent RNA helicase DDX3Y	Ddx3y	11870	28689
Catenin beta-1	Ctnnb1	177155	531838
NHP2-like protein 1	Snu13	145066	10
NADH dehydrogenase [ubiquinone] 1 beta subcomplex subunit 9	Ndufb9	282749	240670
Myelin-associated glycoprotein	Mag	nd	458983
NADH dehydrogenase [ubiquinone] 1 alpha subcomplex subunit 5	Ndufa5	261028	114847
T-complex protein 1 subunit beta	Cct2	nd	42012
Hepatocyte cell adhesion molecule	Hepacam	382643	10
Neutral cholesterol ester hydrolase 1	Nceh1	67654	83049
RNA-binding protein FUS	Fus	158994	nd

Mitochondrial pyruvate carrier 1	Mpc1	240759	71002
Zinc transporter 3	Slc30a3	nd	154554
Septin-8	Septin8	29741	10
Phosphatidylinositol 4-kinase alpha	Pi4ka	5189	7512
Oligodendrocyte-myelin glycoprotein	Omg	175837	163629
Probable ribonuclease ZC3H12C	Zc3h12c	30686	10
Phospholipid phosphatase-related protein type 2	Plppr2	4272	4465
IQ motif and SEC7 domain-containing protein 1	Iqsec1	55449	10
Neuronal-specific septin-3	Septin3	175192	434686
Unconventional myosin-Va	Myo5a	81710	470462
Cysteine and glycine-rich protein 1	Csrp1	345095	10
cAMP-dependent protein kinase type II-beta regulatory subunit	Prkar2b	nd	465302
Voltage-dependent calcium channel subunit alpha-2/delta-2	Cacna2d2	nd	nd
Ras-related protein Rab-5C	Rab5c	397121	10
Putative tyrosine-protein phosphatase auxilin	Dnajc6	nd	nd
Sodium channel subunit beta-2	Scn2b	192249	312358
1-phosphatidylinositol 4,5-bisphosphate phosphodiesterase beta-1	Plcb1	9536	13209
Mitochondrial pyruvate carrier 2	Mpc2	70376	nd
Rap1 GTPase-activating protein 1	Rap1gap	7359	8640
Histone H3.3	H3-3a	396218	262802
Protein NipSnap homolog 1	Nipsnap1	496405	332158
Histone H2B type 1-B	Hist1h2bb	316912	1726136
Adaptin ear-binding coat-associated protein 1	Necap1	nd	nd
Mitogen-activated protein kinase 1	Mapk1	nd	nd
Ras-related protein Rab-7a	Rab7a	326044	395952
Poly(rC)-binding protein 1	Pcbp1	190356	nd
Vesicle-associated membrane protein-associated protein A	Vapa	132551	357083
NADH-ubiquinone oxidoreductase chain 5	Mtnd5	331335	517069
Cytochrome c oxidase subunit 6A1, mitochondrial	Cox6a1	9018	nd
Intercellular adhesion molecule 5	Icam5	110630	376655
Peroxiredoxin-5, mitochondrial	Prdx5	nd	nd
Triosephosphate isomerase	Tpi1	nd	nd
Lamin-B2	Lmnb2	nd	nd
Syntaxin-binding protein 6	Stxbp6	nd	nd
ATP synthase subunit f, mitochondrial	Atp5mf	1107107	1086255
Myeloid-associated differentiation marker	Myadm	257776	287205

Quinone oxidoreductase-like protein 2	Cryzl2	nd	nd
Surfeit locus protein 4	Surf4	nd	nd
Sphingosine 1-phosphate receptor 1	S1pr1	nd	nd
Septin-4	Septin4	182842	227819
Nck-associated protein 1	Nckap1	20934	106178
DnaJ homolog subfamily A member 2	Dnaja2	nd	53141
FAST kinase domain-containing protein 3, mitochondrial	Fastkd3	nd	nd
Wiskott-Aldrich syndrome protein family member 1	Wasf1	nd	61937
Probable cationic amino acid transporter	Slc7a14	32619	32822
Junction plakoglobin	Jup	nd	nd
Multifunctional protein ADE2	Paics	nd	nd
Peripherin	Prph	471775	471775
T-complex protein 1 subunit eta	Cct7	nd	27998
Microtubule-associated protein RP/EB family member 3	Mapre3	58878	10
Protein MAL2	Mal2	237959	nd
CD9 antigen	Cd9	201780	127062
Citrate synthase, mitochondrial	Cs	5517	10454
Peroxiredoxin-4	Prdx4	nd	nd
Transmembrane protein 163	Tmem163	76760	102095
Lon protease homolog, mitochondrial	Lonp1	nd	nd
Heterogeneous nuclear ribonucleoprotein A1	Hnrnpa1	nd	456257
Transmembrane protein 65	Tmem65	55313	55313
Zinc transporter SLC39A7	Slc39a7	nd	2815
Probable G-protein coupled receptor 158	Gpr158	nd	nd
Lymphocyte antigen 6H	Ly6h	507119	628754
Ribosome-releasing factor 2, mitochondrial	Gfm2	612804	699965
Glutathione S-transferase LANCL1	Lancl1	nd	nd
Coatomer subunit beta'	Copb2	2628	5083
Hyaluronidase-1	Hyal1	nd	nd
Tetraspanin-7	Tspan7	63977	219206
Protein stum homolog	Stum	nd	nd
Band 4.1-like protein 2	Epb41l2	nd	307957
Mdm2-binding protein	Mtbp	nd	nd
60S ribosomal protein L10a	Rpl10a	nd	nd
Sphingomyelin phosphodiesterase 3	Smpd3	15750	nd
NADH dehydrogenase [ubiquinone] 1 alpha subcomplex subunit 13	Ndufa13	263207	243732

Flotillin-1	Flot1	nd	nd
Microtubule-actin cross-linking factor 1	Macf1	nd	nd
Dual specificity mitogen-activated protein kinase kinase 7	Map2k7	nd	nd
Four and a half LIM domains protein 1	Fhl1	109690	10
Vesicle-associated membrane protein-associated protein B	Vapb	158785	223104
Histone H1.4	H1-4	153243	121257
Cytoplasmic dynein 1 light intermediate chain 1	Dync1li1	24419	27316
Glycerophosphocholine cholinephosphodiesterase ENPP6	Enpp6	19738	88783
General vesicular transport factor p115	Uso1	38961	119715
Cell cycle exit and neuronal differentiation protein 1	Cend1	nd	nd
Rho guanine nucleotide exchange factor 38	Arhgef38	691818	10
Prenylcysteine oxidase	Pcyox1	nd	110016
Cytosol aminopeptidase	Lap3	216603	10
Cytochrome b-c1 complex subunit 6, mitochondrial	Uqcrh	nd	nd
Non-POU domain-containing octamer-binding protein	Nono	37863	69900
Peptidyl-prolyl cis-trans isomerase FKBP8	Fkbp8	152553	127399
Gamma-aminobutyric acid receptor subunit alpha-1	Gabra1	11533	175677
Synaptotagmin-12	Syt12	nd	nd
CDP-diacylglycerol--inositol 3-phosphatidyltransferase	Cdipt	87338	141878
NADH-ubiquinone oxidoreductase chain 4	Mtnd4	24393	25431
ATP synthase protein 8	Mtatp8	313045	10
Leucine-rich repeat transmembrane neuronal protein 3	Lrrtm3	42523	10
Signal-induced proliferation-associated 1-like protein 2	Sipa1l2	231777	10
Mitochondrial amidoxime reducing component 2	Marc2	nd	nd
Cell cycle control protein 50A	Tmem30a	nd	53189
Cadherin-22	Cdh22	57937	10
Regulator of G-protein signaling 7	Rgs7	nd	nd
Solute carrier family 25 member 51	Slc25a51	nd	nd
Succinate dehydrogenase [ubiquinone] cytochrome b small subunit, mitochon	Sdhd	37961	nd
Ganglioside-induced differentiation-associated protein 1	Gdap1	197625	191767
Protein PRRC2A	Prrc2a	nd	nd
IQ motif and SEC7 domain-containing protein 3	Iqsec3	nd	nd
Polypyrimidine tract-binding protein 2	Ptbp2	nd	nd
Acylglycerol kinase, mitochondrial	Agk	nd	nd
Tectonin beta-propeller repeat-containing protein 1	Tecpr1	21480	nd
ATP-dependent RNA helicase A	Dhx9	nd	413742

Heterogeneous nuclear ribonucleoprotein D0	Hnrnpd	147758	412560
V-type proton ATPase subunit D	Atp6v1d	192951	292880
SLIT-ROBO Rho GTPase-activating protein 2	Srgap2	300548	10
ATPase Asna1	Asna1	nd	nd
Protein bassoon	Bsn	47729	365974
Calcium uniporter protein, mitochondrial	Mcu	nd	43399
F-actin-capping protein subunit alpha-1	Capza1	208938	10
Sodium/calcium exchanger 1	Slc8a1	nd	333555
Ubiquitin-conjugating enzyme E2 D3	Ube2d3	97375	181778
Pre-mRNA-processing-splicing factor 8	Prpf8	14715	24636
PH and SEC7 domain-containing protein 3	Psd3	233638	271306
Short/branched chain specific acyl-CoA dehydrogenase, mitochondrial	Acadsb	nd	nd
Catenin alpha-1	Ctnna1	nd	nd
Laminin subunit alpha-3	Lama3	291985	10
AFG3-like protein 2	Afg3l2	7305	10
Choline transporter-like protein 1	Slc44a1	nd	nd
Calcium/calmodulin-dependent 3',5'-cyclic nucleotide phosphodiesterase 1B	Pde1b	nd	nd
Misshapen-like kinase 1	Mink1	18666	nd
Glutathione hydrolase 7	Ggt7	37430	nd
Protein-arginine deiminase type-2	Padi2	22686	10
RasGAP-activating-like protein 1	Rasal1	6034	19394
Heterogeneous nuclear ribonucleoprotein U-like protein 2	Hnrnpul2	131169	1077008
Serine/threonine-protein phosphatase PP1-gamma catalytic subunit	Ppp1cc	31704	nd
ATPase family AAA domain-containing protein 3	Atad3	14629	10
Proline-rich transmembrane protein 2	Prrt2	nd	nd
Ryanodine receptor 2	Ryr2	nd	nd
Sodium-coupled neutral amino acid transporter 3	Slc38a3	112672	103894
AP-3 complex subunit mu-1	Ap3m1	nd	nd
Potassium voltage-gated channel subfamily A member 1	Kcna1	nd	nd
Flotillin-2	Flot2	nd	nd
Casein kinase II subunit alpha	Csnk2a1	nd	283722
Sodium/potassium-transporting ATPase subunit beta-3	Atp1b3	130630	126356
BTB/POZ domain-containing protein 17	Btbd17	nd	nd
Aquaporin-4	Aqp4	nd	nd
cAMP-dependent protein kinase catalytic subunit alpha	Prkaca	nd	201580
ADP-ribosylation factor-like protein 8B	Arl8b	125037	176817

Inactive hydroxysteroid dehydrogenase-like protein 1	Hsdl1	32918	nd
OCIA domain-containing protein 2	Ociad2	19612	75995
Heterogeneous nuclear ribonucleoprotein A0	Hnrnpa0	178046	289479
Connector enhancer of kinase suppressor of ras 2	Cnksr2	8595	10
Amine oxidase [flavin-containing] A	Maoa	nd	nd
AP-3 complex subunit beta-2	Ap3b2	65114	108785
Solute carrier family 12 member 2	Slc12a2	nd	80793
Cytochrome c oxidase subunit 6C	Cox6c	821492	1065216
Gephyrin	Gphn	nd	nd
NADH dehydrogenase [ubiquinone] 1 alpha subcomplex subunit 7	Ndufa7	386141	267527
Heterogeneous nuclear ribonucleoprotein Q	Syncrip	127136	177414
Isoform 4 of Synaptotagmin-7	Syt7	nd	42266
Sorting and assembly machinery component 50 homolog	Samm50	227662	326496
ATP-dependent 6-phosphofructokinase, liver type	Pfkl	83347	nd
Phosphofurin acidic cluster sorting protein 1	Pacs1	24103	18210
Neutral amino acid transporter A	Slc1a4	nd	51538
Phospholemman	Fxyd1	nd	nd
Protein NDRG4	Ndrg4	80361	66605
cAMP and cAMP-inhibited cGMP 3',5'-cyclic phosphodiesterase 10A	Pde10a	nd	nd
Renin receptor	Atp6ap2	nd	nd
Mitochondrial proton/calcium exchanger protein	Letm1	nd	92535
Cytochrome c oxidase subunit 5B, mitochondrial	Cox5b	nd	nd
Rho GTPase-activating protein 1	Arhgap1	28978	nd
GON-4-like protein	Gon4l	112878	10
Na(+)/H(+) exchange regulatory cofactor NHE-RF1	Slc9a3r1	nd	nd
Proline-rich protein 5	Prr5	139010	10
Programmed cell death protein 6	Pdcd6	nd	24443
Proline-rich transmembrane protein 3	Prrt3	53837	nd
Isoform GlyT-1A of Sodium- and chloride-dependent glycine transporter 1	Slc6a9	nd	9231
Serine incorporator 1	Serinc1	nd	nd
Peptidyl-prolyl cis-trans isomerase A	Ppia	nd	251994
LanC-like protein 2	Lancl2	nd	nd
RNA-binding motif protein, X chromosome	Rbmx	222162	409362
NADH-cytochrome b5 reductase 3	Cyb5r3	463175	672668
Rho-related GTP-binding protein RhoG	Rhog	nd	nd
Neurabin-2	Ppp1r9b	nd	nd

Amyloid-beta A4 precursor protein-binding family B member 2	Apbb2	nd	nd
Peroxiredoxin-like 2A	Prxl2a	140195	234647
Ectonucleoside triphosphate diphosphohydrolase 2	Entpd2	11718	10019
Guanine nucleotide-binding protein G(olf) subunit alpha	Gnal	nd	369479
Lysophosphatidylserine lipase ABHD12	Abhd12	nd	nd
Protein NDRG3	Ndrg3	nd	nd
Nucleoside diphosphate kinase A	Nme1	nd	nd
Cytochrome b-c1 complex subunit 7	Uqcrb	231023	505035
Cytoplasmic FMR1-interacting protein 2	Cyfip2	nd	222765
Cytochrome c, somatic	Cycs	nd	nd
Complement component 1 Q subcomponent-binding protein, mitochondrial	C1qbp	nd	nd
Coiled-coil domain-containing protein 150	Ccdc150	132166	10
Cell adhesion molecule 4	Cadm4	106989	105694
Serine/threonine-protein kinase MARK2	Mark2	nd	4800
DnaJ homolog subfamily A member 3, mitochondrial	Dnaja3	nd	nd
A-kinase anchor protein 5	Akap5	nd	81992
Mitofusin-2	Mfn2	nd	nd
Ras-related protein Rab-33B	Rab33b	265425	10
Lysosome-associated membrane glycoprotein 5	Lamp5	19921	15358
CB1 cannabinoid receptor-interacting protein 1	Cnrip1	nd	nd
Metabotropic glutamate receptor 3	Grm3	nd	175908
PHD finger protein 24	Phf24	nd	nd
Profilin-2	Pfn2	nd	230946
SH3-containing GRB2-like protein 3-interacting protein 1	Sgip1	24952	10
NADH-cytochrome b5 reductase 1	Cyb5r1	nd	nd
Dual specificity mitogen-activated protein kinase kinase 4	Map2k4	24568	45763
Creatine kinase B-type	Ckb	93856	85848
Acetylcholine receptor subunit delta	Chrnd	nd	nd
Transient receptor potential cation channel subfamily M member 1	Trpm1	1245662	10
Talin-2	Tln2	15367	15282
Keratin, type I cytoskeletal 42	Krt42	144614	109958
Isoform 1 of Glycerol kinase	Gk	nd	nd
ATP synthase membrane subunit DAPIT, mitochondrial	Atp5md	956234	1030567
Cleft lip and palate transmembrane protein 1 homolog	Clptm1	nd	nd
Cytochrome c oxidase subunit 1	Mtco1	nd	nd
Conserved oligomeric Golgi complex subunit 1	Cog1	nd	nd

GTP-binding protein Di-Ras2	Diras2	732542	636602
Arf-GAP with SH3 domain, ANK repeat and PH domain-containing protein 2	Asap2	816505	919669
Serine/arginine-rich splicing factor 3	Srsf3	nd	1139210
Serine beta-lactamase-like protein LACTB, mitochondrial	Lactb	nd	nd
60S ribosomal protein L12	Rpl12	108931	134522
Myristoylated alanine-rich C-kinase substrate	Marcks	108406	263744
Aspartate aminotransferase, cytoplasmic	Got1	119457	10
Alpha-synuclein	Snca	243904	169019
ATP synthase subunit e, mitochondrial	Atp5me	564170	447625
V-type proton ATPase 21 kDa proteolipid subunit	Atp6v0b	nd	nd
Mitochondrial import receptor subunit TOM40 homolog	Tomm40	56700	nd
Neuron-specific calcium-binding protein hippocalcin	Hpca	21861632	nd
Coatomer subunit gamma-2	Copg2	3717	nd
Stomatin-like protein 2, mitochondrial	Stoml2	3701	5064
Synaptopodin	Synpo	94028	91616
Arf-GAP with GTPase, ANK repeat and PH domain-containing protein 2	Agap2	nd	nd
Ganglioside-induced differentiation-associated protein 1-like 1	Gdap1l1	nd	nd
Heterogeneous nuclear ribonucleoprotein M	Hnrnpm	254152	326837
Gamma-adducin	Add3	nd	nd
Mitochondrial carrier homolog 1	Mtch1	169972	254223
Serine/threonine-protein kinase MRCK beta	Cdc42bpb	nd	nd
NADH dehydrogenase [ubiquinone] iron-sulfur protein 5	Ndufs5	nd	104986
NADH dehydrogenase [ubiquinone] 1 beta subcomplex subunit 3	Ndufb3	178153	10
G-protein coupled receptor 37-like 1	Gpr37l1	24836	nd
Inactive dipeptidyl peptidase 10	Dpp10	123230	nd
Serine/threonine-protein kinase SIK3	Sik3	148823	10
Exportin-2	Cse1l	nd	38622
7-methylguanosine phosphate-specific 5'-nucleotidase	Nt5c3b	nd	nd
Vomeronasal type-1 receptor 53	Vmn1r53	37925	nd
Ras-related protein Rab-5A	Rab5a	132154	207529
Protein SON	Son	10033	nd
2-oxoglutarate dehydrogenase, mitochondrial	Ogdh	35336	nd
Neuronal growth regulator 1	Negr1	266270	527598
Dehydrodolichyl diphosphate synthase complex subunit Dhdds	Dhdds	56784	nd
Adipocyte plasma membrane-associated protein	Apmap	25374	38395
Major prion protein	Prnp	162187	184755

NADH dehydrogenase [ubiquinone] flavoprotein 3, mitochondrial	Ndufv3	101020	nd
Carbonic anhydrase 2	Ca2	143194	nd
Heat shock protein 75 kDa, mitochondrial	Trap1	nd	657291
Microtubule-associated protein 2	Map2	nd	nd
Protein lin-7 homolog C	Lin7c	101357	111702
E3 ubiquitin-protein ligase PDZRN3	Pdzrn3	213948	nd
Regulating synaptic membrane exocytosis protein 1	Rims1	nd	nd
Cytochrome b-c1 complex subunit 10	Uqcr11	83663	89845
Dynactin subunit 1	Dctn1	20517	72510
Prelamin-A/C	Lmna	nd	nd
Neural cell adhesion molecule L1	L1cam	6922	147699
cGMP-dependent 3',5'-cyclic phosphodiesterase	Pde2a	16090	117833
Activator of 90 kDa heat shock protein ATPase homolog 1	Ahsa1	57415	112731
Transmembrane protein 14C	Tmem14c	nd	nd
PI-PLC X domain-containing protein 3	Plcxd3	nd	nd
ATP-dependent DNA helicase DDX11	Ddx11	nd	nd
Fatty acid synthase	Fasn	nd	nd
Electron transfer flavoprotein subunit alpha, mitochondrial	Etfa	33452	10
Ras-related protein Rap-1b	Rap1b	nd	185959
Band 4.1-like protein 3	Epb41l3	nd	nd
Drebrin	Dbn1	nd	2537120
Isoform 1 of Cell division control protein 42 homolog	Cdc42	nd	112084
Isoform 3 of Reticulon-3	Rtn3	nd	677390
Ras-related protein Rab-11B	Rab11b	nd	717885
Disks large homolog 2	Dlg2	nd	3976
Guanine nucleotide-binding protein subunit alpha-11	Gna11	nd	62730
Isoform 4 of Reticulon-3	Rtn3	nd	66508
Histone H3.2	Hist1h3b	nd	1025776
Heterogeneous nuclear ribonucleoprotein H2	Hnrnph2	nd	nd
Serine/threonine-protein kinase DCLK1	Dclk1	nd	666718
Transcriptional activator protein Pur-beta	Purb	nd	96196
Succinate--CoA ligase [ADP-forming] subunit beta, mitochondrial	Sucla2	nd	308826
Solute carrier family 2, facilitated glucose transporter member 3	Slc2a3	nd	419249
Protein RUFY3	Rufy3	nd	69305
GTPase HRas	Hras	nd	659658
Probable ATP-dependent RNA helicase DDX5	Ddx5	nd	274379

cAMP-dependent protein kinase catalytic subunit beta	Prkacb	nd	148600
Transitional endoplasmic reticulum ATPase	Vcp	nd	166154
Adhesion G protein-coupled receptor L3	Adgrl3	nd	10230
F-actin-capping protein subunit alpha-2	Capza2	nd	552532
Heterogeneous nuclear ribonucleoproteins C1/C2	Hnrnpc	nd	544766
Vacuolar protein sorting-associated protein 35	Vps35	nd	6833
Brain-specific angiogenesis inhibitor 1-associated protein 2	Baiap2	nd	1085078
Keratin, type I cytoskeletal 16	Krt16	nd	267692
MAP kinase-activating death domain protein	Madd	nd	nd
Serine/threonine-protein phosphatase 2A 56 kDa regulatory subunit gamma is	Ppp2r5c	nd	82628
Endoplasmic reticulum chaperone BiP	Hspa5	nd	nd
N-terminal EF-hand calcium-binding protein 2	Necab2	nd	nd
Neural cell adhesion molecule 2	Ncam2	nd	108643
ADP-ribosylation factor GTPase-activating protein 1	Arfgap1	nd	nd
Enoyl-CoA hydratase, mitochondrial	Echs1	nd	nd
TOM1-like protein 2	Tom1l2	nd	111925
Ileal sodium/bile acid cotransporter	Slc10a2	nd	nd
V-type proton ATPase subunit G 2	Atp6v1g2	nd	55050
Metabotropic glutamate receptor 2	Grm2	nd	nd
Cytoplasmic FMR1-interacting protein 1	Cyfip1	nd	nd
CLIP-associating protein 2	Clasp2	nd	38562
Phosphatase and actin regulator 1	Phactr1	nd	5559
Syntaphilin	Snph	nd	100690
Uncharacterized protein KIAA0513	Kiaa0513	nd	39563
Dolichyl-diphosphooligosaccharide--protein glycosyltransferase subunit 1	Rpn1	nd	183939
Transcriptional activator protein Pur-alpha	Pura	nd	120273
Dihydropyrimidinase-related protein 1	Crmp1	nd	81642
Rho guanine nucleotide exchange factor 2	Arhgef2	nd	68606
Pyrroline-5-carboxylate reductase 3	Pycr3	nd	3836
Ras-related protein Rab-15	Rab15	nd	4258936
Ubiquitin-conjugating enzyme E2 N	Ube2n	nd	nd
40S ribosomal protein S3a	Rps3a	nd	25809
Small nuclear ribonucleoprotein Sm D1	Snrpd1	nd	14323
Serine/threonine-protein phosphatase 2A 56 kDa regulatory subunit epsilon is	Ppp2r5e	nd	nd
Tight junction protein ZO-2	Tjp2	nd	nd
Microtubule-associated protein RP/EB family member 2	Mapre2	nd	99096

Dystrophin	Dmd	nd	17133
Aly/REF export factor 2	Alyref2	nd	6621
Neurexin-1	Nrxn1	nd	131582
Heterogeneous nuclear ribonucleoprotein D-like	Hnrnpdl	nd	nd
Cytoskeleton-associated protein 5	Ckap5	nd	nd
60S ribosomal protein L18a	Rpl18a	nd	nd
Apoptosis-inducing factor 1, mitochondrial	Aifm1	nd	nd
Filamin-C	Flnc	nd	1926120
Regulator of microtubule dynamics protein 3	Rmdn3	nd	nd
Adenylate cyclase type 5	Adcy5	nd	174123
Long-chain-fatty-acid--CoA ligase 3	Acsl3	nd	nd
Pituitary adenylate cyclase-activating polypeptide type I receptor	Adcyap1r1	nd	11141
Chondroitin sulfate proteoglycan 5	Cspg5	nd	69776
COP9 signalosome complex subunit 7a	Cops7a	nd	nd
Probable dolichyl pyrophosphate Glc1Man9GlcNAc2 alpha-1,3-glucosyltransf	Alg8	nd	nd
Actin-binding LIM protein 3	Ablim3	nd	nd
MAGUK p55 subfamily member 2	Mpp2	nd	68516
Endoplasmin	Hsp90b1	nd	nd
RUN domain-containing protein 3A	Rundc3a	nd	nd
Serine/arginine-rich splicing factor 10	Srsf10	nd	nd
Band 4.1-like protein 1	Epb41l1	nd	45096
Oxidation resistance protein 1	Oxr1	nd	nd
Translocon-associated protein subunit delta	Ssr4	nd	nd
Stonin-2	Ston2	nd	nd
Autophagy-related protein 2 homolog A	Atg2a	nd	104300
BDNF/NT-3 growth factors receptor	Ntrk2	nd	nd
Acetyl-CoA acetyltransferase, mitochondrial	Acat1	nd	nd
Endonuclease domain-containing 1 protein	Endod1	nd	110288
Receptor expression-enhancing protein 2	Reep2	nd	nd
Ras-related protein Rab-18	Rab18	nd	nd
G protein-regulated inducer of neurite outgrowth 3	Gprin3	nd	nd
Phenylalanine--tRNA ligase beta subunit	Farsb	nd	nd
Diacylglycerol kinase epsilon	Dgke	nd	nd
Serine racemase	Srr	nd	nd
UBX domain-containing protein 6	Ubxn6	nd	nd
Kinesin-like protein KIF21A	Kif21a	nd	2460

Trace amine-associated receptor 5	Taar5	nd	5725
Catenin delta-2	Ctnnd2	nd	12376
26S proteasome regulatory subunit 6B	Psmc4	nd	nd
Formin-like protein 2	Fmnl2	nd	252197
Importin-9	Ipo9	nd	nd
Sperm-associated antigen 4 protein	Spag4	nd	450351
NADH dehydrogenase [ubiquinone] 1 alpha subcomplex assembly factor 2	Ndufaf2	nd	nd
Gamma-aminobutyric acid receptor subunit gamma-2	Gabrg2	nd	29892
Syntaxin-12	Stx12	nd	190319
NADPH--cytochrome P450 reductase	Por	nd	nd
Vacuolar protein sorting-associated protein 33A	Vps33a	nd	nd
3-ketoacyl-CoA thiolase A, peroxisomal	Acaa1a	nd	nd
40S ribosomal protein S10	Rps10	nd	50265
Cyclin-G-associated kinase	Gak	nd	nd
Serine/threonine-protein kinase 32C	Stk32c	nd	3172
Vasopressin V1b receptor	Avpr1b	nd	nd
3-hydroxy-3-methylglutaryl-coenzyme A reductase	Hmgcr	nd	nd
Eukaryotic translation initiation factor 3 subunit F	Eif3f	nd	nd
Protein rogdi homolog	Rogdi	nd	108297
Heat shock 70 kDa protein 12B	Hspa12b	nd	74929
NEDD8-conjugating enzyme Ubc12	Ube2m	nd	nd
Mitogen-activated protein kinase 3	Mapk3	nd	125738
Rac GTPase-activating protein 1	Racgap1	nd	862967
60S ribosomal protein L4	Rpl4	nd	nd
Phospholipid transfer protein C2CD2L	C2cd2l	nd	72046
Peroxisomal multifunctional enzyme type 2	Hsd17b4	nd	nd
Phosphoinositide 3-kinase regulatory subunit 4	Pik3r4	nd	nd
Protein SCAI	Scai	nd	nd
NADH dehydrogenase [ubiquinone] 1 beta subcomplex subunit 6	Ndufb6	nd	97504
FERM, ARHGEF and pleckstrin domain-containing protein 1	Farp1	nd	114120
60S ribosomal protein L23	Rpl23	nd	nd
DNA-directed RNA polymerase I subunit RPA1	Polr1a	nd	nd
Isoform 3 of Myelin expression factor 2	Myef2	nd	125789
60S ribosomal protein L22	Rpl22	nd	nd
SH3 and multiple ankyrin repeat domains protein 1	Shank1	nd	nd
Vesicle-associated membrane protein-associated protein B	Vapb	nd	223104

Actin-related protein 3B	Actr3b	nd	nd
Protein lifeguard 2	Faim2	nd	nd
40S ribosomal protein S24	Rps24	nd	26062
Protein Mpv17	Mpv17	nd	nd
Protein Red	Ik	nd	611010
Protein piccolo	Pclo	nd	nd
Casein kinase II subunit beta	Csnk2b	nd	nd
Myelin-associated oligodendrocyte basic protein	Mobp	nd	136096
60S ribosomal protein L10-like	Rpl10l	nd	nd
Protein unc-13 homolog A	Unc13a	nd	10086
ATPase WRNIP1	Wrnip1	nd	124858
Lamina-associated polypeptide 2, isoforms beta/delta/epsilon/gamma	Tmpo	nd	nd
ATPase family AAA domain-containing protein 1	Atad1	nd	30249
Serine/arginine-rich splicing factor 7	Srsf7	nd	261511
Integrin alpha-L	Itgal	nd	209032
Pyruvate kinase PKM	Pkm	nd	nd
Protein spire homolog 2	Spire2	nd	239082
Protein disulfide-isomerase A6	Pdia6	nd	nd
Myosin regulatory light polypeptide 9	Myl9	nd	153374
60S ribosomal protein L7a	Rpl7a	nd	98114
Rho guanine nucleotide exchange factor 7	Arhgef7	nd	nd
Seizure 6-like protein 2	Sez6l2	nd	nd
PRA1 family protein 2	Praf2	nd	57811
Peroxiredoxin-1	Prdx1	nd	36546
Fatty-acid amide hydrolase 1	Faah	nd	nd
D-3-phosphoglycerate dehydrogenase	Phgdh	nd	1142633
Erlin-2	Erlin2	nd	nd
60S ribosomal protein L7	Rpl7	nd	36899
FXYD domain-containing ion transport regulator 6	Fxyd6	nd	219018
Metallo-beta-lactamase domain-containing protein 2	Mblac2	nd	3393
1-acyl-sn-glycerol-3-phosphate acyltransferase alpha	Agpat1	nd	nd
Cell adhesion molecule 1	Cadm1	nd	nd
Lysophosphatidic acid receptor 1	Lpar1	nd	nd
Vacuolar protein sorting-associated protein 13A	Vps13a	nd	nd
Breast carcinoma-amplified sequence 1 homolog	Bcas1	nd	127398
Sorbin and SH3 domain-containing protein 2	Sorbs2	nd	nd

AP-5 complex subunit zeta-1	Ap5z1	nd	nd
NAD(P) transhydrogenase, mitochondrial	Nnt	nd	126269
Abl interactor 1	Abi1	nd	nd
Tyrosine-protein phosphatase non-receptor type 9	Ptpn9	nd	nd
Receptor-type tyrosine-protein phosphatase S	Ptprs	nd	nd
Lysophospholipid acyltransferase 7	Mboat7	nd	nd
Protein LYRIC	Mtdh	nd	nd
Catenin alpha-3	Ctnna3	nd	nd
Pyruvate dehydrogenase protein X component, mitochondrial	Pdhx	nd	nd
60S ribosomal protein L30	Rpl30	nd	nd
Membrane protein MLC1	Mlc1	nd	nd
60S acidic ribosomal protein P0	Rplp0	nd	33274
Myotubularin-related protein 5	Sbf1	nd	nd
Coxsackievirus and adenovirus receptor homolog	Cxadr	nd	nd
AP-3 complex subunit delta-1	Ap3d1	nd	57275
Paraspeckle component 1	Pspc1	nd	nd
Serine/arginine-rich splicing factor 1	Srsf1	nd	155260
Dynactin subunit 4	Dctn4	nd	83999
STE20/SPS1-related proline-alanine-rich protein kinase	Stk39	nd	76144
Dynein light chain 2, cytoplasmic	Dynll2	nd	149434
Splicing factor 3B subunit 1	Sf3b1	nd	nd
Receptor-type tyrosine-protein phosphatase zeta	Ptprz1	nd	nd
ATP synthase subunit ATP5MPL, mitochondrial	Atp5mpl	nd	285923
Lysophospholipase D GDPD1	Gdpd1	nd	nd
Leucine-rich repeat-containing protein 57	Lrrc57	nd	nd
Calmodulin-2	Calm2	nd	209482
Isoform L of Receptor-type tyrosine-protein phosphatase delta	Ptprd	nd	6650
Mitochondrial fission 1 protein	Fis1	nd	nd
Neuroligin-3	Nlgn3	nd	nd
Dolichyl-diphosphooligosaccharide--protein glycosyltransferase 48 kDa subur	Ddost	nd	nd
Rab3 GTPase-activating protein non-catalytic subunit	Rab3gap2	nd	nd
Very-long-chain (3R)-3-hydroxyacyl-CoA dehydratase 3	Hacd3	nd	331015
Dedicator of cytokinesis protein 3	Dock3	nd	nd
TRPM8 channel-associated factor 1	Tcaf1	nd	225287
PRA1 family protein 3	Arl6ip5	nd	85987
Cyclin-Y	Ccny	nd	nd

Paralemmin-1	Palm	nd	92497
WD repeat and FYVE domain-containing protein 1	Wdfy1	nd	3787
Core histone macro-H2A.1	H2afy	nd	nd
Cadherin-2	Cdh2	nd	120975
ARF GTPase-activating protein GIT1	Git1	nd	18041
Sodium channel protein type 2 subunit alpha	Scn2a	nd	nd
OX-2 membrane glycoprotein	Cd200	nd	nd
Stabilin-1	Stab1	nd	3288
ATP-binding cassette sub-family A member 7	Abca7	nd	nd
Neurofibromin	Nf1	nd	104962
Mediator of RNA polymerase II transcription subunit 23	Med23	nd	8873
Succinate dehydrogenase cytochrome b560 subunit, mitochondrial	Sdhc	nd	nd
Neuroligin-2	Nlgn2	nd	nd
Large neutral amino acids transporter small subunit 4	Slc43a2	nd	nd
Probable ubiquitin carboxyl-terminal hydrolase FAF-X	Usp9x	nd	nd
Isoform 2 of DIS3-like exonuclease 2	Dis3l2	nd	nd
40S ribosomal protein S7	Rps7	nd	15955
Opalin	Opalin	nd	7567
Olfactory receptor 490	Olfr490	nd	nd
Armadillo repeat protein deleted in velo-cardio-facial syndrome homolog	Arvcf	nd	46577
		nd	
Isoform 1 of Unconventional myosin-XVIIIa	Myo18a	nd	nd
		nd	
CUGBP Elav-like family member 1	Celf1	nd	nd
Ataxin-2	Atxn2	nd	3645
Vesicle-trafficking protein SEC22b	Sec22b	nd	nd
Voltage-gated potassium channel subunit beta-1	Kcnab1	nd	nd

*nd = not determined
